# Photoelectrochemical alcohols oxidation over polymeric carbon nitride photoanodes with simultaneous H_2_ production†

**DOI:** 10.1039/d2ta03660f

**Published:** 2022-07-25

**Authors:** Neeta Karjule, Ravindra S. Phatake, Shmuel Barzilai, Biswajit Mondal, Adi Azoulay, Alexander I. Shames, Michael Volokh, Josep Albero, Hermenegildo García, Menny Shalom

**Affiliations:** Department of Chemistry and Ilse Katz Institute for Nanoscale Science and Technology, Ben-Gurion University of the Negev Beer-Sheva 8410501 Israel mennysh@bgu.ac.il; Department of Chemistry, Nuclear Research Centre-Negev P.O. Box 9001 Beer-Sheva 84910 Israel; Department of Physics, Ben-Gurion University of the Negev Beer-Sheva 8410501 Israel; Instituto Universitario de Tecnología Química (ITQ), Consejo Superior de Investigaciones, Científicas (CSIC), Universitat Politècnica de València (UPV) Avda. de Los Narajos s/n Valencia 46022 Spain

## Abstract

The photoelectrochemical oxidation of organic molecules into valuable chemicals is a promising technology, but its development is hampered by the poor stability of photoanodic materials in aqueous solutions, low faradaic efficiency, low product selectivity, and a narrow working pH range. Here, we demonstrate the synthesis of value-added aldehydes and carboxylic acids with clean hydrogen (H_2_) production in water using a photoelectrochemical cell based solely on polymeric carbon nitride (CN) as the photoanode. Isotope labeling measurements and DFT calculations reveal a preferential adsorption of benzyl alcohol and molecular oxygen to the CN layer, enabling fast proton abstraction and oxygen reduction, which leads to the synthesis of an aldehyde at the first step. Further oxidation affords the corresponding acid. The CN photoanode exhibits excellent stability (>40 h) and activity for the oxidation of a wide range of substituted benzyl alcohols with high yield, selectivity (up to 99%), and faradaic efficiency (>90%).

## Abstract 2

We report a photoelectrochemical (PEC) approach for the selective oxidation of alcohols to value-added products with simultaneous hydrogen (H_2_) generation using polymeric carbon nitride (CN) film as a photoanode.

## Graphical abstract

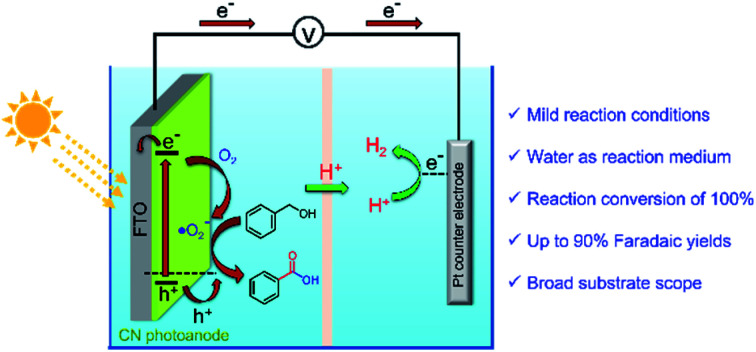

## 1. Introduction

The photoelectrochemical (PEC) generation of solar fuel—typically hydrogen (H_2_)—is a promising technology for energy management. However, it is often coupled with oxygen (O_2_) production, a low-value product. Hence, some research efforts have been dedicated towards combining H_2_ production with the transformation of various chemicals into value-added products, which should occur on the surface of the photoanode.^1–5^ In particular, aldehydes and carboxylic acids are valuable industrial and medicinal compounds on their own and may also be used for further chemical syntheses.^6,7^ Their synthesis usually involves the selective oxidation of an alcohol using environmentally unfriendly oxidants, such as permanganates, persulfates, dichromats, and strong inorganic acids.^8,9^ Organic synthesis *via* PEC could be a greener route than traditional methods for the selective oxidation of alcohols.^10,11^ To date, only a few key reports have demonstrated the utility of photoanodic oxidation for the synthesis of valuable chemicals in parallel with H_2_ formation at the cathode.^2,12^

Using a semiconductor film at a solid/liquid junction, where the active material both absorbs solar radiation and catalyzes the photoelectrochemical reaction, is a simple and scalable technique.^13^ Recently, BiVO_4_, WO_3_, and several nanocomposites-based photoelectrodes have been successfully used for oxidation, C–H functionalization, and cross-coupling reactions.^14–16^ Despite the progress in this field, the production of valuable chemicals using PEC is still impeded by low faradaic efficiency, low stability in aqueous solutions, narrow pH ranges (owing to the instability of the involved photoanode materials), low product selectivity, and moderate control over the oxidation process (in case of a sequential oxidation).^17,18^ To improve the activity and stability of the photoanode, two approaches are frequently used: (i) a miscible mixture of a non-aqueous solvent and water is used as the electrolyte solution,^4,16^ and (ii) a cocatalyst is deposited.^19,20^ However, the cocatalyst enhances the oxidative reaction rate at the photoanode, and it also increases O_2_ production, resulting in low product formation and low faradaic efficiency. In addition, in some cases, the density of reaction sites for the adsorption and activation of reactants on the cocatalysts is limited.^21^ Changing the solvent composition may augment chemical production and selectivity but decrease in the rate of the H_2_ evolution reaction (HER) is inevitable. To overcome these challenges, new catalytic materials for the photoanode should be introduced.

Polymeric carbon nitride (CN) is a promising photoanodic semiconductor material for water-splitting PEC because it is composed of abundant and benign elements and has appropriate properties such as suitable band edges for water splitting, an adjustable electronic structure, and UV-visible light absorption capability up to 460 nm.^22^ In addition, other characteristic material features of CN photoanodes—such as morphology, porosity, specific surface area, and crystallinity also significantly impact the PEC water-splitting performance.^23^ However, O_2_ evolution over CN materials is less common due to the challenges posed by the valence band (VB) position and the sluggish oxygen evolution reaction (OER) reaction kinetics. In our previous findings,^24^ cocatalysts were deposited into porous CN photoanodes to overcome the OER kinetic barrier and increase the consumption rate of photogenerated charges, particularly the holes. This, in turn, increased the photoanode's stability *versus* (*vs.*) photo-corrosion.

Despite very significant progress,^25^ low-cost CN-based photoanodes in water-splitting PEC are still an order of magnitude less performant than state-of-the-art photoanodes (based on heterojunctions that contain BiVO_4_, WO_3_, Fe_2_O_3_, TiO_2_, *etc.*) because of sluggish oxygen production.^26,27^ In a water-splitting process using CN, hole (h^+^) extraction and O_2_ production are considered more difficult steps because water oxidation is slow since it involves four electron–hole (e^−^–h^+^) pairs.^28^ This limitation of CN becomes a key advantage for PEC alcohol oxidation. In contrast to water oxidation, the oxidation of organic substrates is typically easier, therefore requiring lower photovoltage.^29^ Under illumination, CN photoanodes can directly oxidize an organic substrate in an aqueous electrolyte, while suppressing the competing water oxidation reaction. Additionally, CN is stable in a wide pH range with a high affinity for organic molecules on its surface and a suitable VB position for alcohol oxidation;^24,30^ hence, a CN-based photoanode is an ideal candidate for such PECs.

Herein, we report the use of a CN-based photoanode in PEC for the selective oxidation of benzyl alcohols (BnOH) to benzaldehydes (BZD) and benzoic acids (BZA). The optimized CN photoanode demonstrates good stability and high efficiency: it affords complete conversion of reactant (100%) with excellent faradaic yield (>99%) for the oxidation of BnOH to benzoic acid at the photoanode (in aqueous media at room temperature) together with good H_2_ production at the cathode. The PEC oxidation reaction mechanism was elucidated with the help of transient absorption spectroscopy (TAS), electron paramagnetic resonance (EPR) measurements, isotope-labeling experiments, and density functional theory (DFT) calculations. The scope of suitable substrate molecules was successfully enlarged by the oxidation of several other aryl alcohols to their corresponding carboxylic acids under the same conditions. Finally, we demonstrated the compatibility of the reported PEC system with heteroatom-containing substrates by preparing heterocyclic acids commonly used in pharmaceutical applications.

## 2. Results and discussion

### 2.1 PEC oxidation of benzyl alcohol on the CN photoanode

**Fig. 1:**
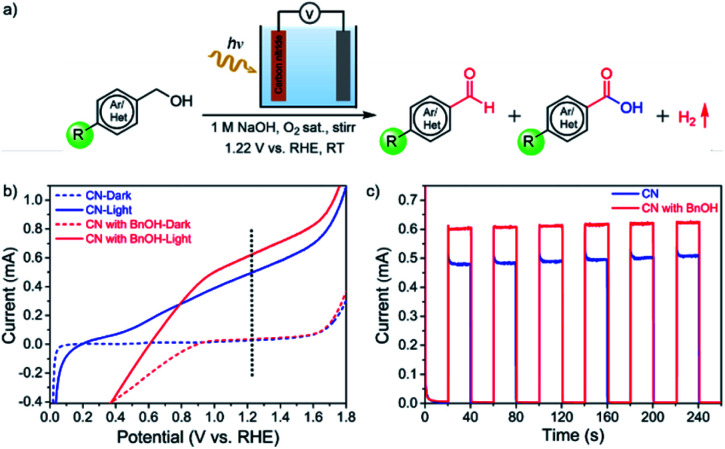
PEC oxidation: reaction conditions and characterization of photoanodes. (a) PEC oxidation scheme of primary alcohols using a CN film on a transparent conductive fluorine-doped tin oxide (FTO) electrode as the photoanode in the presence of O_2_ as the oxidant and a Pt cathode. (b) Linear sweep voltammetry (LSV) curves of CN without (blue) (under argon) and with BnOH (red) (in the presence of O_2_) under illumination (continuous lines) or in the dark (dashed lines) at a scan rate of 20 mV s^−1^. (c) Chronoamperometry (current *vs.* time) of CN electrode in 1 M NaOH aqueous solution (aqueous solution, pH = 13.9) without (blue) and with (red) BnOH (substrate concentration: 10 mM BnOH, 1 M NaOH aqueous solution, in the presence of O_2_).

CN electrodes were prepared by a two-step method consisting of doctor-blading followed by thermal treatment (see the experimental section for the detailed procedure). The UV-vis absorption spectra, surface morphology, X-ray diffraction (XRD) patterns, and X-ray photoelectron spectroscopy (XPS) of the films are consistent with our previous reports (Fig. S1–S4†).^24,28^ Here, we used CN electrodes modified with reduced graphene oxide (rGO) for their porous structure, good electron (e^−^) mobility, and high electrochemically active surface area. In our previous work,^24,28^ without the addition of rGO, a CN photoanode showed very low photocurrent density and was unstable. Upon rGO integration, the photocurrent density and stability increased, mainly because of the conductivity and e^−^ diffusion improvement due to the presence of rGO (Fig. S5†). Therefore, the metal-free CN electrodes modified with rGO were chosen for PEC BnOH oxidations. As illustrated in Fig. 1a, we propose to use CN photoanodes for the PEC oxidation of BnOH and its derivatives to aldehydes or carboxylic acids with molecular O_2_ as the oxidant. The BnOH oxidation reaction was performed under 1-sun illumination (AM 1.5 G, 100 mW cm^−2^) using a CN photoanode, 1 M NaOH aqueous solution (pH = 13.9) saturated with O_2_ as an electrolyte, a Pt-foil counter electrode (cathode), and an Ag/AgCl reference electrode.

Fig. 1b shows the linear sweep voltammograms (LSV) under dark and illumination conditions. The CN photoanode has a low onset potential, with a measured photocurrent of 0.50 ± 0.03 mA at 1.22 V *vs.* a reversible hydrogen electrode (RHE). In the presence of BnOH and saturated with O_2_, the photocurrent increases to 0.60 ± 0.02 mA, suggesting that BnOH is more easily oxidized at the photoanode than water (Fig. 1b). We note that the current decay below 0.9 V is due to concurrent oxygen reduction reaction (ORR). Periodic on/off illumination of CN without and with BnOH-containing electrolytes (saturated O_2_ solution) at a fixed potential of 1.22 V *vs.* RHE affords a stable photocurrent: 0.62 ± 0.02 mA under illumination in the presence of BnOH (Fig. 1c). Under chopped light, the CN photoresponse of the electrode is consistent with the LSVs (Fig. S6a†). From this point onwards, all reported organic oxidation reactions were conducted under an applied voltage of 1.22 V (*vs.* RHE). Because of the lack of a cocatalyst, at this voltage bias, the production of molecular O_2_ from the oxygen evolution reaction (OER) is negligible.

### 2.2 Optimization studies

**Fig. 2:**
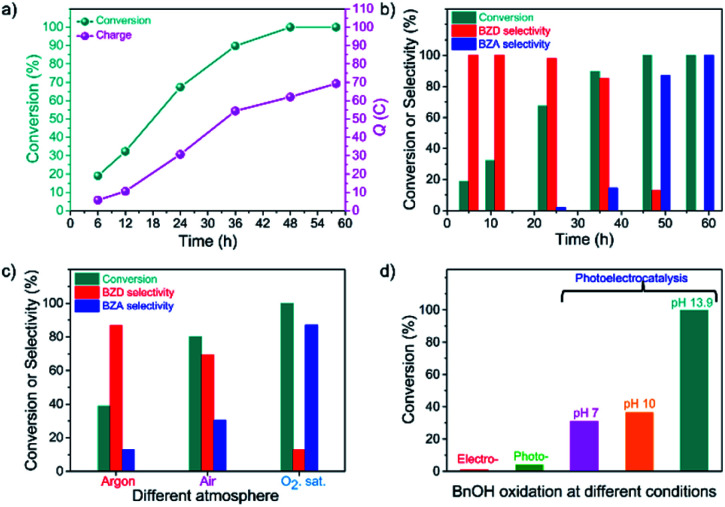
PEC oxidation of BnOH over CN photoanodes. (a) Conversion and charge passed (*Q*) *vs.* reaction time. (b) Conversion and corresponding selectivity of BZD or BZA *vs.* reaction time. (c) Conversion and corresponding selectivity of BZD or BZA under different reaction atmospheres (argon, air, and O_2_) at the same reaction time (substrate concentration: 10 mM BnOH, 1 M NaOH aqueous solution). (d) Comparison between the conversion rate of BnOH oxidation under different conditions: electrocatalysis (no illumination), photocatalysis (no applied voltage), and photoelectrocatalysis at different pH values (pH = 7, 10, and 13.9).

The oxidation of BnOH over the CN photoanode at different reaction times (or charge passed, *Q*) was carried out at 1.22 V *vs.* RHE under light irradiation and in the presence of O_2_ (Fig. S6b†). Fig. 2a shows the conversion of BnOH and charge passed *vs.* reaction time. As the reaction proceeds, the concentration of BnOH decreases while the concentration of generated BZD increases. The selectivity for BZD after 12 h and 24 h is 100% and ∼98%, respectively (Table S1†). After 36 h, the BnOH oxidation reaction conversion reaches 90%, and the selectivity towards BZD is >85% (Fig. 2b). Longer reaction times lead to further oxidation of BZD to BZA until full conversion of the starting BnOH. After 48 h, the major product is BZA (87.1% selectivity). A complete selectivity towards BZA is obtained after a reaction time of 58 h with an excellent faradaic yield of >99% (Fig. 2b). The PEC process enables the control of product selectivity with time because BnOH is initially oxidized to BZD (up to a selectivity of 85.0%) and then further oxidized to BZA (selectivity of 100%, indicating the complete PEC oxidation of BnOH to BZA).

To explore the reaction mechanism and optimize the reaction conditions, we conducted the BnOH oxidation under air and argon environments (identical reaction times, Table S2†).^12^ LSVs under O_2_, air, and Ar are provided in Fig. S7a:† the observed photocurrent varies depending on the atmosphere, leading to dissimilar conversion and selectivity between BZD and BZA formation (Fig. 2c). In the absence of O_2_—under Ar—the conversion rate of BnOH is low (39%), whereas it increases to 80% in air because of the presence of O_2_. However, in the absence of O_2_, the initial photocurrent is lower than in the presence of O_2_, and a slow decrease is observed after a few hours (Fig. S7b†). In the Ar atmosphere, conversion of BnOH to BZA over CN significantly decreases since electrons cannot activate O_2_ to produce ˙O_2_^−^ radicals. A dramatic increase in photocurrent is achieved in a solution saturated with O_2_; a reaction conversion of almost 100% is achieved, with 13% and 87% selectivity towards BZD and BZA, respectively, revealing that O_2_ has a vital role in the oxidation reaction.^31^

Different experimental methods were used to distinguish the respective contributions of electrocatalysis and photocatalysis (Fig. 2d).^32^ The BnOH oxidation performance is summarized in Table S3.† In electrocatalytic mode (without illumination; with a voltage bias of 1.22 V *vs.* RHE), BnOH oxidation was negligible. Under photocatalytic conditions (under illumination; without a voltage bias), barely any BnOH was consumed (4%). These results indicate that in the electrocatalytic route, molecular O_2_ (*i.e.*, without activation) cannot directly participate in the BnOH oxidation reaction and that in the photocatalytic route, BnOH undergoes sluggish oxidation with poor reaction conversion. In contrast, high conversion of BnOH (100%) is obtained *via* the PEC process, demonstrating that the combination of photoexcitation and voltage bias is necessary for the reaction to occur at an acceptable rate.

We investigated the effect of pH on the BnOH PEC oxidation, unveiling that the oxidation reaction slows down at lower pHs (Fig. 2d).^33^ The slow oxidation kinetics at low pH allows the obtention of BZD from BnOH with high selectivity (>99%). At high pH, in contrast, a higher photocurrent with good long-term stability allows further oxidation to BZA (Table S3†). Resembling water oxidation,^24^ in BnOH oxidation, higher photocurrent and higher rates are observed at higher pH. The conversion of alcohols to acids is achieved only at pH 13 mainly because the generation rate of reactive intermediates as ˙O_2_^−^ is much higher (due to increased stability) in an alkaline medium, which ultimately increases the reaction rate.^34,35^

### 2.3 Isotope-labeling experiments

**Fig. 3:**
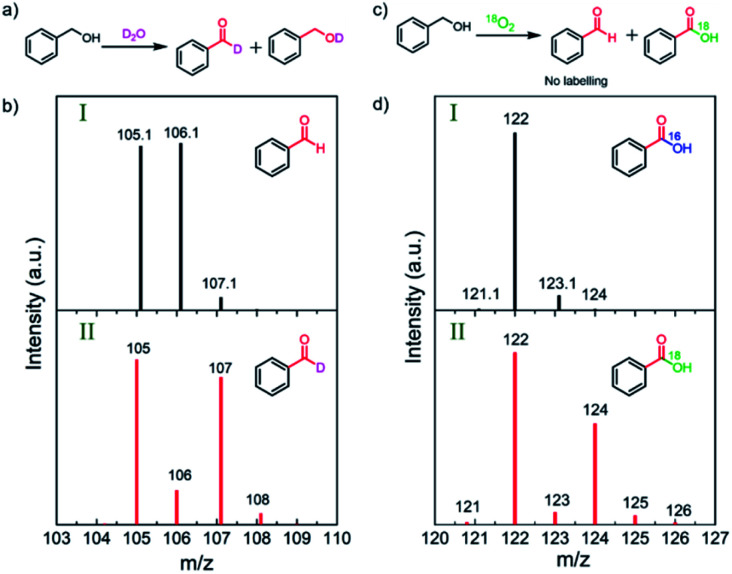
D_2_O and ^18^O_2_ labeling experiments for BnOH oxidation. (a) PEC oxidation of BnOH using a D_2_O solution containing 1 M NaOH. (b) Mass spectra of BZD produced in (I) H_2_O and (II) D_2_O showing labeling in BZD. (c) PEC oxidation reaction of BnOH in ^18^O-labeled oxygen gas (^18^O_2_). (d) Mass spectra of BZD produced in (I) ^16^O_2_ and (II) ^18^O_2_ showing labeling in benzoic acid (^18^OH group of the carboxylic acid). The analyzed reaction conditions are illuminated CN photoanode at 1.22 V *vs.* RHE for 48 h; the substrate concentration is 10 mM BnOH in 1 M NaOH aqueous solution.

To uncover the source of the oxygen atoms involved in the oxidation process, systematic D_2_O and ^18^O_2_ isotope-labeling experiments were conducted (Fig. 3).^31,36,37^ D_2_O labeling experiments were carried out by replacing H_2_O with D_2_O in the 1 M NaOH electrolyte solution. The GC-MS results show that deuterium incorporates into BnOH and BZD by hydrogen–deuterium exchange (Fig. 3a–b and Fig. S8–S9†).

To further clarify the exact role of molecular O_2_ in the BnOH oxidation, the PEC oxidation of BnOH under an isotope-labeled ^18^O atmosphere was performed. In the first oxidation process, no ^18^O-labeled BZD was detected after photoelectrocatalysis, ruling out the inclusion of O atoms from O_2_ or ˙O_2_^−^ radicals into BZD (Fig. S10†).^38^ However, ^18^O_2_ labeling experiments reveal that the second oxygen in BZA originates from O_2_ and not from water (Fig. 3c–d, marked in green in the chemical structure).

### 2.4 Mechanistic investigations

**Fig. 4:**
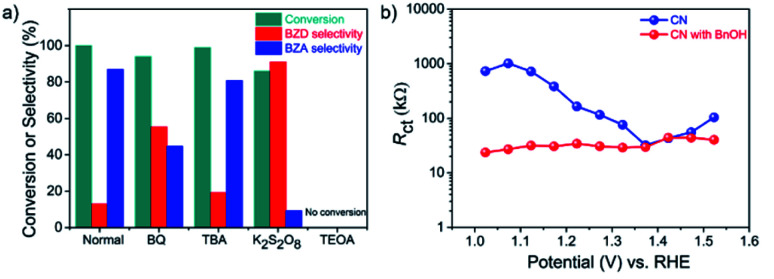
Mechanistic and electrochemical investigations. (a) BnOH oxidation reaction over a CN photoanode in the presence of various radical scavengers for 48 h (“Normal” was conducted without scavenger); BQ as a ˙O_2_^−^ radical scavenger, 0.5 mM; TBA as an ˙OH radical scavenger, 1 mM; K_2_S_2_O_8_ as an e^−^ scavenger, 1 mM; TEOA as a h^+^ scavenger, (10% *v*/*v*). (b) Charge transfer resistance (*R*_ct_) of CN photoanode at various applied potentials without and with BnOH (in the presence of O_2_).

A series of control experiments were conducted by adding different scavengers to the system to further elucidate the reaction mechanism (Fig. 4a).^39,40^ In the presence of 1,4-benzoquinone (BQ), an ˙O_2_^−^ radical scavenger, the conversion of BnOH remained similar. However, the yield of BZA was reduced significantly, indicating that the oxidation step of BZD to BZA involves ˙O_2_^−^ radicals, in line with the ^18^O_2_ labeling measurements. The addition of *tert*-butyl alcohol (TBA), a hydroxyl-radical (˙OH) scavenger, had little influence, disclosing that ˙OH has a negligible effect on the reaction and, hence, on the BZA yield (Table S4†).

In contrast, the photocurrent density and the yield of BZA were significantly decreased by the addition of potassium persulfate (K_2_S_2_O_8_), an e^−^ scavenger, confirming the importance of O_2_ activation to form ˙O_2_^−^ radicals by photogenerated e^−^ in BnOH oxidation. Finally, the addition of triethanolamine (TEOA), a h^+^ scavenger, completely suppressed the BnOH conversion, indicating that photogenerated h^+^ play a key role in the oxidation of BnOH. Summarizing this section, h^+^ and ˙O_2_^−^ radicals are the main active species for the PEC BnOH oxidation reaction under visible light illumination. Electrochemical impedance spectroscopy (EIS) measurements reveal that the presence of BnOH lowers the charge transfer resistance (*R*_ct_) of CN (Fig. S11 and S12†).^41^ EIS studies at applied potentials of 1.02–1.52 V *vs.* RHE indicate better h^+^ extraction properties from the electrode to the solution in the presence of BnOH over a wide potential range (Fig. 4b).

In order to gain information of the BnOH oxidation mechanism, transient absorption (TA) measurements have been performed in a CN dispersion in acetonitrile (355 nm laser excitation) under N_2_ and O_2_ atmosphere (Fig. S13 and S14,† respectively).^42^ Under an O_2_ saturated atmosphere (Fig. S14†), the CN TA spectrum shows a continuous band along the measured spectrum, with a relative maximum intensity around 660 nm. In our results, the slow component in the CN TA under O_2_ atmosphere can be attributed to long lived photogenerated h^+^ in the CN valence band (VB).

**Fig. 5:**
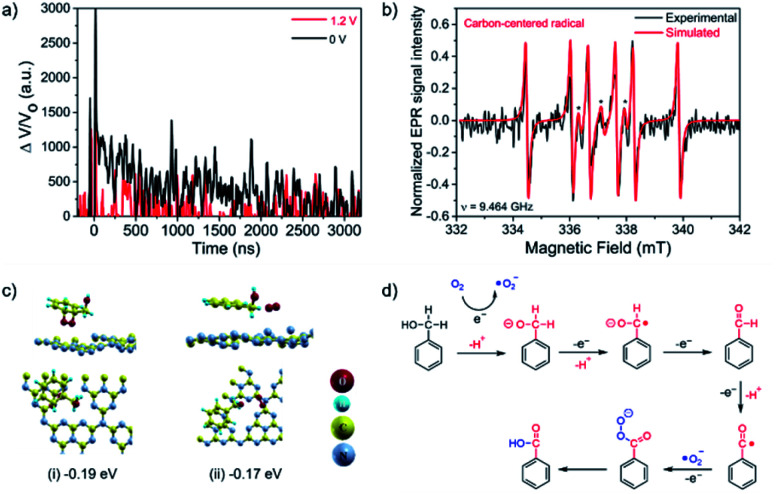
Mechanistic studies: experiments and calculations. (a) TA kinetics of CN photoelectrode in O_2_-saturated LiClO_4_ solution (0.1 M in acetonitrile) and 64 mM BnOH upon no applied bias voltage (black) and 1.2 V *vs.* NHE (red). Pt wire and Ag/AgCl electrode were used as counter and reference electrodes, respectively. Laser excitation 355 nm. Monitored wavelength of 660 nm. (b) EPR detection of carbon-centered radicals formed during BnOH oxidation using DMPO as the spin-trapping agent (black asterisks indicate characteristic DMPO peaks). (c) DFT calculations showing the favorable configuration of BnOH and O_2_ over CN; The adsorption energy beneath each illustration was computed relative to the energy sum of the relaxed CN and the isolated relaxed moiety. (d) Proposed mechanism of the PEC oxidation of BnOH over a CN photoanode.

In order to further investigate the PEC reaction mechanism, TA experiments have been also carried out using CN electrodes under 355 nm laser excitation in the presence of BnOH after O_2_ purging. The TA kinetics of the CN electrode were measured at 660 nm with either no applied bias voltage or 1.2 V *vs.* NHE (Fig. 5a).^43^ Under no bias, the TA decay follows the expected bimodal behavior. The calculated lifetimes after experimental data fitting to eqn (1) were 120.6 ns and 2.03 μs for the fast and slow components, respectively. However, under applied bias voltage 1.2 V *vs.* NHE, the TA signal was completely quenched. These results indicate that light-induced h^+^ in the CN VB could not have enough oxidation potential to oxidize BnOH, while addition of the applied bias voltage would promote the prompt h^+^ transfer to BnOH.^44^ These results are in good agreement with our control experiments, where photocatalytic and electrocatalytic BnOH oxidation showed negligible reaction conversion.

Overall, the combination of the photogenerated e^−^ in the CN conduction band (CB) and the applied oxidation potential of 1.22 V *vs.* RHE are the driving forces of the outstanding performance of the CN photoanodes for the PEC BnOH oxidation under these conditions. Electron paramagnetic resonance (EPR) spectroscopy was performed under an O_2_ atmosphere by adding the radical spin trapping agent DMPO (5,5-dimethyl-1-pyrroline *N*-oxide) to the electrolyte, confirming the existence of a radical intermediate (Fig. 5b).

In the EPR results, we didn't detect ˙O_2_^−^ radicals; it could be that reactive oxygen species, such as ˙O_2_^−^ radicals, have short lifetimes in an aqueous solution with high pH.^34^ The six new peaks in the fitted results are attributed to carbon-centered radicals, implying that these intermediates are formed during the reaction.^45^ Typically, carbon-cantered radicals, which can be produced by reactive ˙O_2_^−^ radicals or h^+^, are regarded as the crucial intermediates for PEC BnOH oxidation.^31^

The first essential step in most heterogeneous catalytic reactions is the adsorption of reactants on the catalyst surface.^46,47^ Therefore, DFT calculations were preformed to compute the adsorption energies of the initial moieties ((a) molecular O_2_, (b) BnOH, and (c) BnOH adjacent to O_2_) and intermediate reaction products ((d) BZD, (e) BZA) over CN (Fig. 5c and S15†). From an energetic viewpoint, the adsorption energy of O_2_ is about −0.22 eV with a preferred location above the meso pore (Fig. S15a†). It is worth mentioning that for a dissociated molecule, the adsorption energy was relatively high, exceeding −1.5 eV. The same site is also preferred by the BnOH, with an adsorption energy of −0.12 eV (Fig. S15b†). When both moieties are located above this site, each moiety changes its preferred location, and the obtained integral adsorption energy is −0.17 eV. Thus, the adsorbed O_2_ molecule (O^ads^_2_) can effectively bind to the CN photoanode surface, even in the vicinity of a BnOH molecule.

From an energetic viewpoint, O^ads^_2_ reacts with the BnOH on graphitic carbon nitride^48^ with a low adsorption energy of −0.17 eV (Fig. 5c). In this configuration, and under both illumination and applied potential, the CN photoanode, which generates e^−^ and h^+^ charge carriers, can efficiently catalyze the reduction of O^ads^_2_ into ˙O_2_^−^ radicals using the available e^−^. Hence, the effective reaction rate for the BnOH oxidation into BZA depends to a large extent on the rate of adsorption and activation of BnOH and O_2_ molecules on the CN surface (Fig. S16†).

We propose a mechanism for the PEC oxidation of BnOH over CN photoanodes (Fig. 5d). Under the combined illumination and applied potential, the CN photoanode generates e^−^ and h^+^ charge carriers.^49^ The photogenerated e^−^ (at the CB) reduces O_2_ molecules to ˙O_2_^−^ radicals while the h^+^ (at the VB) oxidizes the BnOH molecules to the corresponding carbon-centered radicals (Fig. S17†).^50^ Simultaneously, ˙O_2_^−^ radicals abstract protons from the BnOH molecules to form carbon-centered radicals, which are then oxidized to BZD. The ˙O_2_^−^ radicals play the important role of promoting the rapid consumption of photogenerated e^−^ in the reaction, thus accelerating the separation of photogenerated e^−^ and h^+^, which is beneficial for the BnOH oxidation.^51,52^ Subsequently, the BZD molecules are oxidized by h^+^ at the electrode to form carbon-centered radicals, which themselves react with ˙O_2_^−^ to form BZA. During the oxidation process, most e^−^ transfer to the Pt counter electrode (the cathode), thanks to the applied bias, where they reduce the adsorbed hydrogen (H_ads_) formed by water dissociation into H_2_.^53^ It is important to note that the uptake of e^−^ by O_2_ at the photoanode enhances the stability of the CN electrodes because it removes e^−^ that are far from the conductive substrate. Thus, less recombination occurs and the photogenerated h^+^ lifetime is extended.

**Fig. 6:**
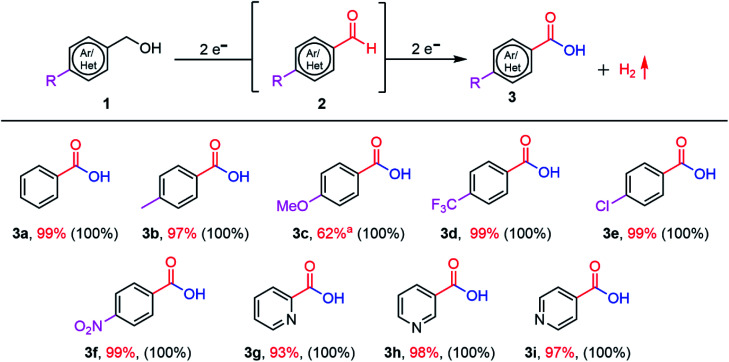
Scope of the substituted BnOH used as substrates. Reaction conditions: 10 mM of alcohol (0.18 mmol) in 1 M NaOH aqueous solution (pH 13.9) saturated with O_2_ as an electrolyte, CN photoanode as the working electrode. PEC was conducted in constant potential mode (1.22 V *vs.* RHE) under 1-sun illumination. The reaction conversion (in parentheses) was determined by GC-MS and ^1^H NMR. The amount of oxidation product (3) was determined by ^1^H NMR using dibromomethane as an internal standard. ^a^The oxidation of 4-methoxybenzyl alcohol yields 38% 4-methoxybenzaldehyde.

After finding the optimal reaction conditions and understanding the underlying oxidation mechanism, we extended the scope of demonstrated PEC oxidation substrates to BnOH substituted with –CH_3_, –OCH_3_, –Cl, –CF_3_, and –NO_2_ groups (Fig. 6 and Table S5†). The reaction conversion and amount of oxidation product (3) were determined by GC-MS and ^1^H NMR measurements. BnOH derivatives substituted with electron-donating groups (–CH_3_, –OCH_3_) and electron-withdrawing groups (–CF_3_, –Cl, and –NO_2_) are efficiently oxidized into the corresponding carboxylic acids (100% conversion)

The observed photocurrent varies depending on the substrate identity; hence, the PEC time to achieve complete conversion itself varies (a total charge of around 69–70 C passed is necessary to complete the 4e^−^ oxidation reactions).

In the case of BnOH substituted with –OCH_3_ (4-methoxybenzyl alcohol), the reactant is fully consumed (100% conversion), but the corresponding carboxylic acid is obtained in low yield (62%). –OCH_3_ is a strong electron-donating group; therefore, the resonant structures of the 4-methoxybenzyl alcohol radicals are more stabilized in the *para* orientation, which does not promote effective aldehyde-to-carboxylic acids conversion.^54^ Heteroatom-containing substrates (pyridine derivatives **3g–3i**) can also be successfully converted to the corresponding carboxylic acids in high yield (≥93%). Overall, all the substrates show 100% conversion and faradaic yields ≥92% (except 4-methoxybenzyl alcohol: faradaic yield of 81.3%). The cathodic reaction, that is, HER, was measured during BnOH oxidation in a two-compartment cell (Fig. S18†).^12^ The concentration of generated H_2_ increases steadily with time. After 10 h of PEC operation time, 35.6 μmol H_2_ is produced (faradaic yield (%) of 75 ± 8). The non-optimal faradaic yield stems from the reaction of e^−^ with O_2_.

A detailed characterization of the electrodes after 48 h of PEC operation reveals changes in the morphology and the thickness of the CN film (stability measurement, Fig. S19–S20†). XRD and Fourier-transform infrared (FTIR) measurements of CN after a long reaction time indicate structural changes in the CN films (Fig. S21†). FTIR shows that the characteristic peaks of CN slowly disappear with increasing PEC operation time (the strong band at 880–1640 cm^−1^, attributed to the typical stretching vibration of C–N and C–NH heterocycles and the band near 808 cm^−1^, related to the *s*-triazine). The XRD measurements suggest that the CN film on FTO has a lower crystallinity the characteristic peaks of CN are much weaker than before PEC operation. The intensity of the diffraction reflection at ∼27.7° (002 plane of CN) decreases, which indicates that some CN detaches from the FTO surface during operation. Complementary XPS measurements suggest partial oxidation of the CN framework during the PEC oxidation reaction of BnOH (Fig. S22†). In the C 1 s spectrum, the peak corresponding to C–N=C decreases, revealing an increase in the C-to-N atomic ratio (from 0.87 to 2.13) (Table S6†). Hence, the main reason behind the slow decrease in photocurrent lies in the leaching of CN from the FTO surface during operation; we intend to direct future research efforts towards stabilizing the CN film under the working conditions.

## 3. Conclusions

In conclusion, the utilization of CN-based photoanode in PEC has been successfully demonstrated with an organic synthesis producing value-added aldehydes and carboxylic acids while H_2_ is produced at the cathode. Under visible light, excited e^−^ from the semiconductor CN surface activate O_2_ molecules into ˙O_2_^−^ radicals, whereas photogenerated h^+^ oxidize BnOH to BZD or BZA. This setup allows the successful oxidation of multiple substituted aryl alcohols into their corresponding carboxylic acids with excellent yields, up to 99%. The faradaic yields of the oxidation reactions were very high for all substrates (>90%). In the proposed mechanism (backed by TAS, EPR, and isotope-labeling experiments), after a proton is abstracted from BnOH (and then BDZ), oxidation by ˙O_2_^−^ radicals forms carbon-centered radicals as the reaction intermediates towards the BZA product. DFT calculations verified that CN has an excellent ability to adsorb BnOH and O_2_, whereas the rapid reduction of O_2_ by photoexcited e^−^ is responsible for the excellent charge separation. This study shows the compelling potential of utilizing metal-free polymeric CN materials for photoelectrochemical organic transformations. These promising results may lead to safer, more selective and cost-effective chemical reactions.

## 4. Experimental

### 4.1 Materials and reagents

All chemicals were purchased from commercial sources and used as received. Melamine (99%), potassium chloride (KCl, ReagentPlus®, ≥99%), 4-methoxybenzyl alcohol (98%), 4-(trifluoromethyl)benzyl alcohol (98%), 4-chlorobenzyl alcohol (99%), 4-methylbenzyl alcohol (98%), 4-nitrobenzyl alcohol (99%), 4-pyridinemethanol (99.5%), 3-pyridinemethanol (98%), 2-pyridinemethanol (98%), oxygen-^18^O_2_ (99 at% ^18^O, 99% (CP)), deuterium oxide (D_2_O, 99.9 at% D), 1,4-benzoquinone (BQ, ≥98%), *tert*-butyl alcohol (TBA, ≥99.5%), and 5,5-dimethyl-1-pyrroline *N*-oxide (DMPO, ≥97%) for electron paramagnetic resonance (EPR) spectroscopy were purchased from Sigma-Aldrich. Ethylene glycol (EMSURE® Reag. Ph. Eur, Reag. USP) and potassium persulfate (K_2_S_2_O_8_, ≥99%) were purchased from Merck. Dichloromethane (CH_2_Cl_2_, 99.5% amylene-stabilized), ethanol (≥99.9%), acetone (99.5%), and hydrochloric acid (32 wt%, AR grade) were purchased from Bio-Lab Ltd, Israel. Sodium hydroxide (NaOH, 99%), sodium sulphate anhydrous (Na_2_SO_4_, 99%), and potassium hydroxide pellets (KOH, 85%) were purchased from Loba Chemie, India. Benzyl alcohol (BnOH, ≥99.5%) was purchased from Acros Organics. Dibromomethane (CH_2_Br_2_, 99%) was purchased from TCI chemicals. Chloroform-D (CDCl_3_, >99.8%) was purchased from Fluorochem, UK. Dimethyl sulphoxide-*d*_6_ (DMSO-*d*_6_, 99.9%) was obtained from Cambridge Isotope Laboratories. Graphene oxide (GO, 0.4 wt%, >95%) aqueous suspension was purchased from University Wafer Inc., USA (C89/GOSD18004/D). TEOA (99%) was purchased from Glentham Life Sciences, UK. Fluorine-doped tin oxide (FTO) coated glass (TEC15, 12–14 Ω sq^−1^) was purchased from Xop Glass Company, Spain. De-ionized water (DI) was purified using a Millipore Direct-Q3 water purification system (18.2 MΩ cm resistivity).

### 4.2 Materials characterization

UV-vis absorption spectra were measured on a Cary 100 spectrophotometer using the transmittance mode for films over a transparent substrate to measure *T* and a diffuse reflectance accessory (ATR) to estimate the reflectance, *R*. Fourier-transform infrared spectroscopy (FTIR) was performed on a Thermo Scientific Nicolet iS5 FTIR spectrometer (equipped with a Si attenuated total reflection (ATR) accessory). Scanning electron microscopy (SEM) images of the CN electrodes were obtained using an FEI Verios 460L high-resolution SEM equipped with a FEG source and operated at an accelerating voltage, *U*_0_ = 3.5 kV (after sputtering with Pt, ∼10 nm using a Quorum Q150T ES system). X-ray diffraction (XRD) patterns were obtained using a PANalytical's Empyrean diffractometer equipped with a position-sensitive detector X'Celerator. Data were collected with a scanning time of ∼7 min for a 5–60° 2*θ* range using Cu *K*α radiation (*λ* = 1.54178 Å, 40 kV, 30 mA). X-ray photoelectron spectroscopy (XPS) data were obtained from an X-ray photoelectron spectrometer ESCALAB 250 ultrahigh vacuum (1 × 10^−9^ bar) device with an Al *K*α X-ray source and a monochromator. The X-ray beam size was 500 μm, survey spectra were recorded with a pass energy (PE) of 150 eV, and high energy resolution spectra were recorded with a PE of 20 eV. To correct for charging effects, we calibrated all spectra relative to the carbon C 1 s peak positioned at 284.8 eV. The XPS results were processed using the Thermo Scientific™ Avantage software. Continuous-wave X-band (9.4 GHz) EPR measurements were carried out at room temperature (*T* ∼295 K) using a Bruker EMX-220 spectrometer equipped with an Agilent 53150A frequency counter. For the EPR measurements, 2 mL of the sample was collected from the reaction mixture and mixed with DMPO (0.04 mmol) as a radical spin trapping agent.

### 4.3 Transient absorption spectroscopy (TAS)

Laser flash photolysis (LFP) measurements were performed using a Q-switched Nd:YAG laser (Quantel Brilliant, 355 nm, 20 mJ per pulse, 5 ns fwhm) coupled to a mLFP-111 Luzchem miniaturized equipment. This transient absorption spectrometer includes a ceramic xenon light source, a 125 mm monochromator, a Tektronix 9 bit digitizer TDS-3000 series with 300 MHz bandwidth, a compact photomultiplier and a power supply, a cell holder and fiber optic connectors, a fiber optic sensor for laser-sensing pretrigger signal, computer interfaces, and a software package developed in the LabVIEW environment from National Instruments. The LFP equipment supplies 5 V trigger pulses with a programmable frequency and delay. The TA experiments of CN dispersions in acetonitrile were recorded using 10 mm × 10 mm quartz cells with a capacity of 4 mL, while the TA experiments of CN photoelectrodes were recorded using a home-made quartz cell suitable for photoelectrochemistry experiments. A Pt wire (1 mm thick) and Ag/AgCl (saturated KCl) electrodes were used as counter and references electrodes respectively. LiClO_4_ (0.1 M in acetonitrile) was used as the electrolyte. In addition, BnOH (64 mM) was also added to the electrolyte solution and the whole system was O_2_ purged. The absorbance value of the CN samples in acetonitrile dispersions was around 0.6 at the laser excitation wavelength (355 nm). The lifetimes were calculated after experimental data fitting to eqn (1).

1 *F*(*t*) = *a*__1__ e^−*τ*_1_*t*^ + *a*__2__ e^*τ*_2_*t*^

### 4.4 DFT methodology

Based on previous experimental characterization, we considered a supercell with one layer of graphitic carbon nitride (CN) arranged in a tri-*s*-triazine geometry and containing 24 carbon atoms, 32 nitrogen atoms, and a top vacuum layer of ≈28.6 Å. To define the preferred location of the BnOH moiety over the CN surface and to compute its adsorption energies, all the atoms in this supercell were allowed to relax until the residual forces of all the atoms were less than 10^−3^ Ry/B^3^ and the change in energy was less than 5 × 10^−5^ Ry. For the final relaxed structures, a self-consistent convergence criterion of 10^−6^ Ry was imposed. The adsorption energy (*E*_ads_) of each configuration was computed according to eqn (2).

2 *E*_ads_ = *E*^sys^_rlx_ − (*E*^CN^_rlx_ + *E*^BnOH^_rlx_)

where *E*^sys^_rlx_ is the total energy of the system after relaxation, *E*^CN^_rlx_ is the relaxed energy of the CN surface, *E*^BnOH^_rlx_ is the relaxed energy of an isolated BnOH moiety.

The preferred adsorbed configuration of BnOH molecules over the CN surface was used for further calculations to explore the preferred location and configuration of molecular oxygen (O_2_) over a CN surface covered with adsorbed BnOH. The calculations were carried out using the Quantum-Espresso package^55^ and performed on a periodically repeated supercell. The exchange–correlation potential was treated within the Perdew–Burke–Ernzerhof (PBE) generalized gradient approximation (GGA)^56^ and a *k*-mesh of 2 × 2 × 1 was constructed according to the Monkhorst and Pack scheme.^57^ The ion core was described by plane wave (PAW) pseudopotentials^58^ and the valence electrons (1s electron for the H atoms, 2s and 2p electrons for C and N atoms, and 2s and 2p electrons for O_2_) were treated explicitly with a 350 Ry cut-off for the charge density and kinetic 50 Ry for the wave function. To ensure that the results of the calculations are directly comparable, identical conditions and convergence criteria were employed for all systems.

### 4.5 Carbon nitride electrode preparation^24,28^

A melamine–graphene oxide supramolecular paste was formed by blending melamine powder (2.0 g) in ethylene glycol (1.4 mL) with a graphene-oxide aqueous suspension (1.0 mL of a 0.8 wt% GO suspension prepared by concentrating a commercial solution of GO at 0.4 wt%). The formed paste was doctor-bladed (with 2 scotch tape layers) onto FTO, subsequently dried at 90 °C on a hot plate, and finally transferred into a closed (by aluminum foil) 25 mm-diameter glass test tube. (Note: FTO substrates were cleaned by sonication in an aqueous 1% w/v Alconox solution, ethanol, and acetone, and finally dried before usage).

### 4.6 Synthesis of CN

In a 25 mm-diameter glass test tube, 0.2 g of melamine powder were deposited at the bottom of the tube, after which two electrodes (electrode size (length × width): 1.7 cm × 2 cm) were placed in its middle. The test tube was then purged very carefully with N_2_ (g) for 5 min and then covered with aluminum foil. Subsequently, the sample was heated up to 550 °C at a ramp rate of 5 °C min^−1^ and kept at 550 °C for 4 h under a constant N_2_ flow before it was naturally cooled down to room temperature.

### 4.7 Photoelectrochemical (PEC) measurements

All electrochemical measurements were performed using a three-electrode system and an Autolab potentiostat (Metrohm, PGSTAT302N). Pt foil (0.75 cm^2^) and Ag/AgCl (saturated KCl) were used as the counter and reference electrodes, respectively. The electrolyte was a 1 M NaOH aqueous solution (pH = 13.9). All the potentials were converted to reversible hydrogen electrode (RHE) values using eqn (3).

3 *V*_RHE_ = *V*_Ag/AgCl_ + 0.059 × pH + 0.197 (V)

Photocurrents were measured at 1.22 V *vs.* RHE under the illumination of a solar simulator (Newport 300 W Xe arc lamp, equipped with an AM 1.5G and water filters) at a power density of 100 mW cm^−2^, which was calibrated using a thermopile power meter (Model 919-P, Newport), that is, 1-sun conditions. To report photocurrents, a standard deviation (*σ*) from three independent experiments (electrode preparation in separate batches and different PEC measurements with new electrolytes) was calculated, and the values are presented as the average photocurrent ± *σ*. Nyquist plots of the samples were measured in the frequency range from 100 kHz to 10 mHz at an applied voltage of 1.22 V *vs.* RHE. Mott–Schottky measurements were performed in 1.0 M Na_2_SO_4_ aqueous solution (pH = 7) using the same potentiostat at 2000 Hz. Here, the voltage difference between the conduction band (CB) and the flat band potential is set to be 0.2 eV.

### 4.8 PEC oxidation of aryl alcohols

Bulk photoelectrolysis experiments were performed in a 30 mL undivided PEC cell. All the reactions were performed in aqueous solutions. A carbon nitride (CN) electrode (1.7 cm × 2 cm) was used as the working electrode and Pt foil (0.75 cm^2^) and Ag/AgCl (saturated KCl) were used as the counter and reference electrodes, respectively. Photoelectrolysis reactions were performed at a constant potential mode of 1.22 V *vs.* RHE. A PTFE-coated magnetic stir bar (1 cm) was used to stir the reaction mixture at 500 rpm during the reactions. All reactions were performed with a substrate concentration (alcohols) of 10 mM in 18 mL of 1 M NaOH aqueous solution (0.18 mmol). Before each experiment, O_2_ gas (or any other appropriate gas for test reactions) was bubbled into the solution for 10 min at a flow rate of 15 mL min^−1^, then filled for 3 min assuming the concentration of O_2_ reached saturation in the headspace. The area under the chronoamperogram corresponds to the charge passed during the PEC oxidation reaction. The characterization of the products and the reaction yields are given in the product isolation and yield determination section.

### 4.9 GC-MS and NMR

Typically, the reaction mixture was dispersed in CH_2_Cl_2_. GC data were obtained using an Agilent 6850 GC equipped with an Agilent 5973 MSD, working under standard conditions and an Agilent HP5-MS (30 × 0.25 × 0.25) column. The column oven temperature was ramped to 80 °C and held for 2 min, then raised to 300 °C and held for 8 min (heating rate: 30 °C min^−1^). Ultrapure helium (≥99.99%) was adopted as the carrier gas at a flow rate of 1.0 mL min^−1^. The temperatures of the injection port, MS transfer line, and ion source were set at 230 °C, 250 °C, and 300 °C, respectively. A sample volume of 1 μL with a split ratio of 100 : 1 was used. Selected ion monitoring mode (SIM) was adopted for the analysis. GC-MS spectra of the products are provided below (GC-MS data section). Similarly, the samples were extracted, concentrated in a rotary evaporator, and then dispersed in CDCl_3_ or DMSO-*d*_6_ for further NMR detection. The ^1^H and ^13^C NMR spectra were recorded using a Bruker DPX 400 instrument; the chemical shifts, given in ppm, are referenced to the residual solvent peak. Alcohol conversion and product selectivity were calculated according to eqn (4) and (5).4
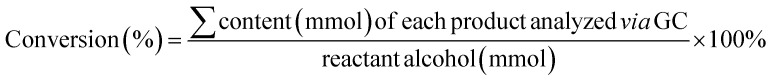
5
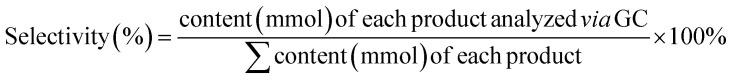


### 4.10 Hydrogen (H_2_) measurements

The amount of photogenerated H_2_ in the reactor headspace was analyzed using an Agilent 7820 GC system equipped with a thermal conductivity detector (TCD). Samples of gases were intermittently withdrawn every 2 h with an A-2 Luer lock gas syringe series purchased from VICI® precision sampling (Pressure-lok® precision analytical syringe). Two-compartment cells were thoroughly sealed with rubber septa and parafilm to prevent any gas leakage. Before the experiment, O_2_ gas was bubbled into the anode solution for 30 min; the solution was then filled with O_2_ for 3 min, assuming the O_2_ concentration reached saturation in the headspace. Meanwhile, Ar (g) was purged into the cathode solution for 30 min to remove any dissolved O_2_. The electrode was held at 1.22 V (*vs.* RHE) under illumination. Faradaic efficiency was calculated using eqn (6).6
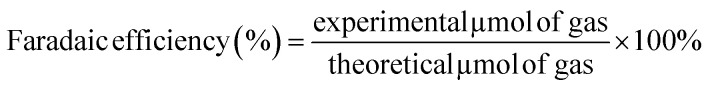


The theoretical amount of gas was calculated from Faraday's law, eqn (7).7
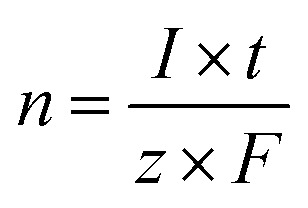
where *n* is the amount of gas (measured in mol), *I* is the current (A), *t* is time (s), *z* is the number of transferred electrons (for H_2_, *z* = 2), and *F* is the Faraday constant (96 485 C mol^−1^).

## Author contributions

N. K. performed most of the experiments, analyzed the data, and wrote the initial draft of the manuscript. R. P. measured and analyzed the GC-MS and NMR data. S. B. performed the DFT calculations. B. M. assisted with the PEC measurements. A. A. and M. V. performed material characterization. A. S. measured and analyzed EPR data. M. V. took part in analysis and writing. J. A. and H. G. performed TAS measurements. M. S. supervised the study and co-wrote the paper. All the authors discussed the results and reviewed the manuscript.

## Conflicts of interest

There are no conflicts to declare.

## Supplementary Material

TA-010-D2TA03660F-s001
