# Supplementary material for: Photoelectrochemical alcohols oxidation over polymeric carbon nitride photoanodes with simultaneous H2 production
Source: J Mater Chem A Mater. 2022 Jul 25;10(31):16585–94. doi: 10.1039/d2ta03660f (PMC9365238; doi:10.1039/d2ta03660f)
Supplement: TA-010-D2TA03660F-s001 [file TA-010-D2TA03660F-s001.pdf]

Electronic Supplementary Material (ESI) for Journal of Materials Chemistry A.
This journal is © The Royal Society of Chemistry 2022

# Electronic Supplementary Information

## Photoelectrochemical alcohols oxidation over polymeric carbon nitride photoanodes with simultaneous $H_2$ production

*Neeta Karjule,*[a] *Ravindra S. Phatake,*[a] *Shmuel Barzilai,*[b] *Biswajit Mondal,*[a] *Adi Azoulay,*[a] *Alexander I. Shames,*[c] *Michael Volokh,*[a] *Josep Albero,*[d] *Hermenegildo García,*[d] *Menny Shalom**[a]

[a]Department of Chemistry and Ilse Katz Institute for Nanoscale Science and Technology, Ben-Gurion University of the Negev, Beer-Sheva 8410501, Israel

[b]Department of Chemistry, Nuclear Research Centre-Negev, P.O. Box 9001, Beer-Sheva 84910, Israel

[c]Department of Physics, Ben-Gurion University of the Negev, Beer-Sheva 8410501, Israel

[d]Instituto Universitario de Tecnología Química (ITQ), Consejo Superior de Investigaciones, Científicas (CSIC), Universitat Politècnica de València (UPV), Avda. de los Narajos s/n, 46022, Valencia (Spain)

E-mail: mennysh@bgu.ac.il

## Supplementary methods

### Product isolation and yield determination for compounds 3a–3f

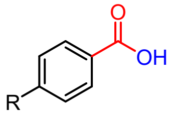


The reaction mixture was transferred into a beaker, and the electrodes were rinsed with water. The product solution was acidified with 2 M aqueous HCl until it reached pH 2. Then, the products were extracted with dichloromethane ($CH_2Cl_2$). The organic layer was dried by adding $Na_2SO_4$ and then filtered through cotton wool to remove the $Na_2SO_4$. Next, the reaction mixture present in the $CH_2Cl_2$ layer was analyzed by gas chromatography–mass spectrometry (GC-MS) to confirm the reaction conversion. Subsequently, the $CH_2Cl_2$ solvent was removed under vacuum, and the product yield was determined by $^1$H nuclear magnetic resonance spectroscopy ($^1$H NMR) using dibromomethane as an internal standard. The GC-MS and NMR spectra of the products are shown below (GC-MS and NMR data section).

### Product isolation and yield determination for compounds 3g–3i

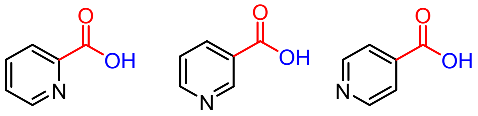


The reaction mixture was transferred to a beaker, and the electrodes were rinsed with water. Then, the reaction mixture was acidified with 2 M aqueous HCl until it reached pH 3–4. A solid residue of NaCl (that forms after acidification with HCl) was obtained after the aqueous layer was evaporated under vacuum. To the solid residue, ethanol (30 mL) was added. After filtration to remove solid residue of NaCl, the ethanol was removed under vacuum, and the product yield was determined by $^1$H NMR.

## Supporting Information Figures

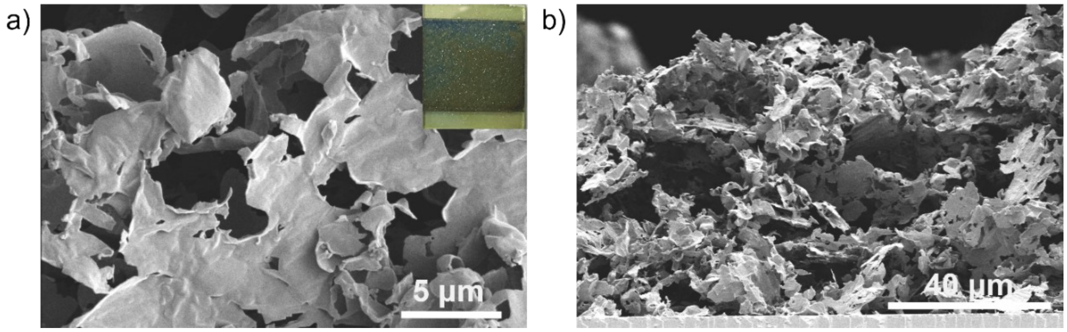


**Fig. S1. Scanning electron microscope (SEM) characterization of CN electrode.** a) Top view (inset: digital image of the CN electrode) and b) cross section (thickness of CN layer above FTO ~70 μm).

The SEM characterization shows a thick and porous structure with a good coverage of the CN film on the conductive FTO substrate.

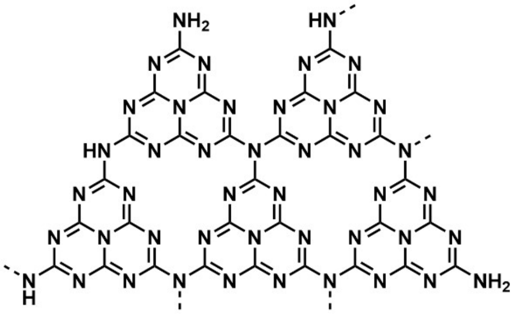

**Fig. S2. Proposed structure of CN showing a melon-type chemical structure**; the formed CN displays a chemical structure like that of melon, *i.e.*, in the form of linear polymers of heptazine linked by –N(H) groups forming sheets and terminal $NH_2$ groups.[1]

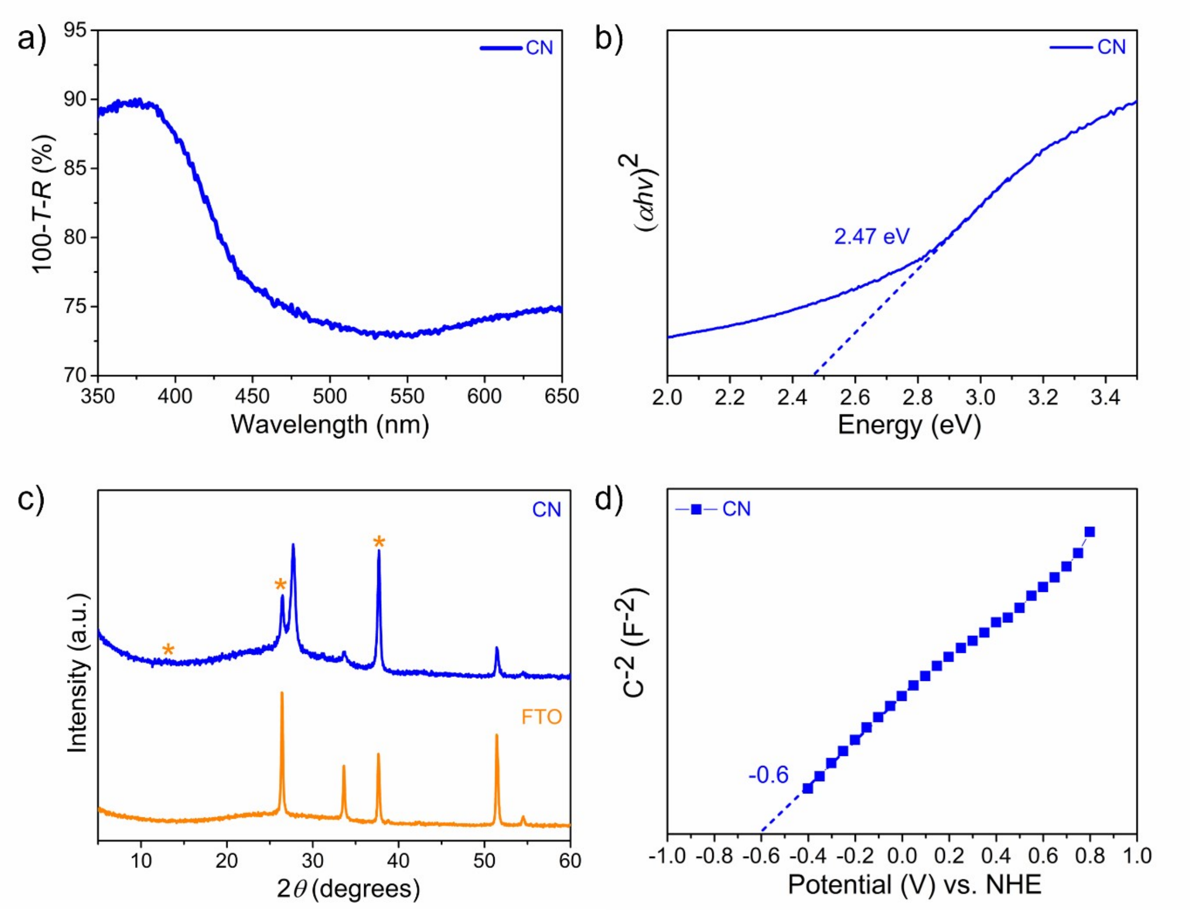


**Fig. S3. Characterization of a CN film on FTO.** a) UV–vis absorbance (in %), b) Tauc plot analysis assuming a direct band gap semiconductor, c) XRD patterns (the orange asterisks denote reflection signals from the FTO), and d) Mott-Schottky plot (1 M aqueous $Na_2SO_4$, pH 7).

The UV–vis absorption spectrum of CN shows that the estimated optical band gap ($E_g$) is 2.47 eV (Fig. S3a–b). Typically, the XRD patterns of CN show two significant diffraction reflections at ~13° and ~27.7°, ascribed to the (100) and (002) planes of CN. Herein, XRD measurements suggest that the crystallinity of the CN film on FTO is low: the characteristic peaks of CN are weak relative to the strong signals from the FTO (marked using orange asterisks, Fig. S3c). The flat-band potential, estimated from electrochemical Mott-Schottky analysis of a CN electrode, is –0.6 V *vs.* NHE and suggests an n-type behavior (Fig. S3d). According to their $E_g$, the valence band (VB) and conduction band (CB) positions of CN are 1.67 V and –0.8 V *vs*. NHE, respectively.

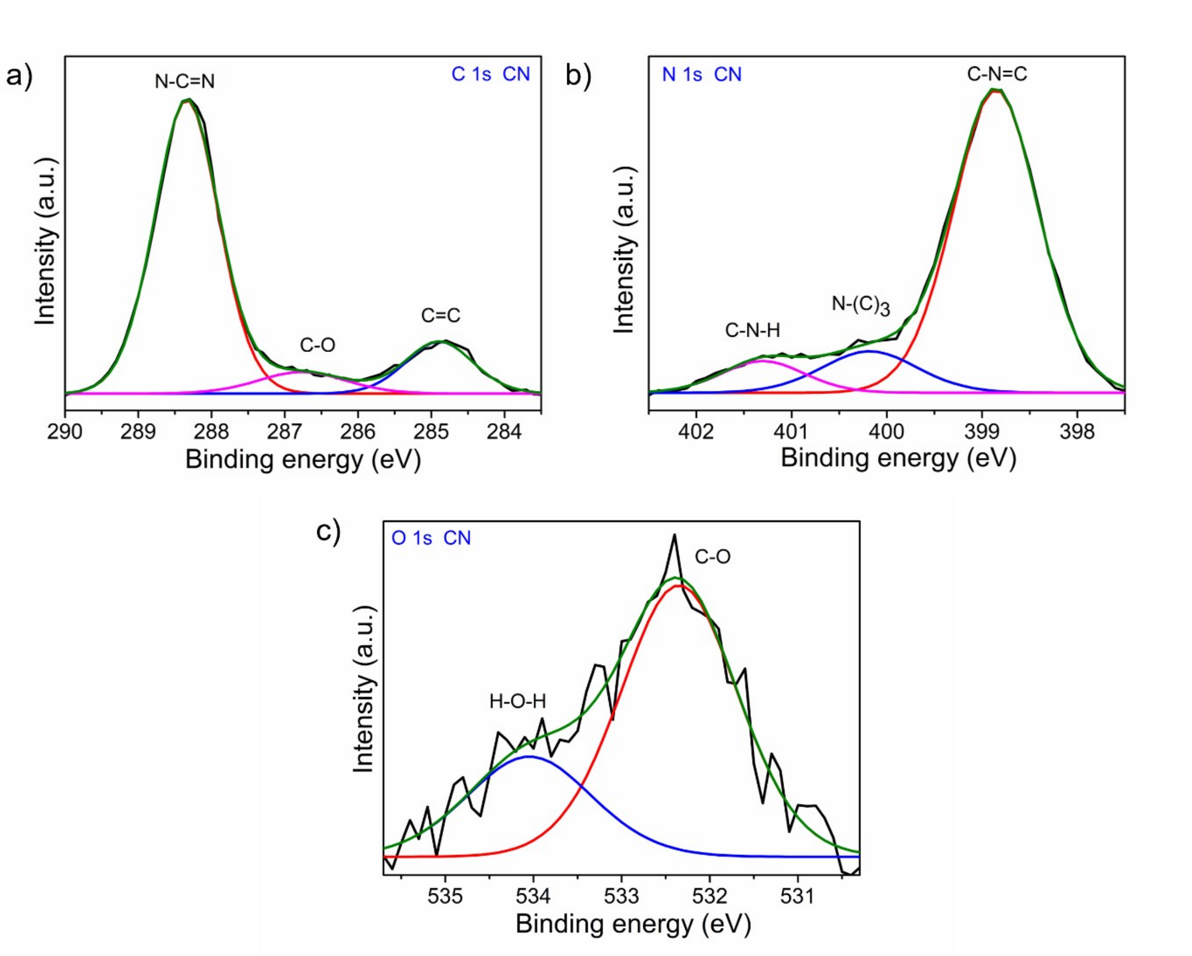


**Fig. S4. High-resolution XPS spectra of CN electrodes.** A) C 1s, b) N 1s, c) O 1s. Experimental spectra are plotted in black; green lines represent the fitted spectra; magenta, blue, and red are the individual deconvoluted fitted peaks.

The chemical states of CN films were investigated by XPS (Fig. S4). In the C 1s spectrum, the binding energies at 284.9 eV and 288.3 eV correspond to C–C bonds and C–N=C moieties, respectively. The peak at 286.7 eV, which was observed for CN, belongs to C–O (surface-adsorbed atmospheric $O_2$). The N 1s spectrum displays three peaks centered at 398.9, 400.2, and 401.3 eV, are assigned to C–N=C bonds, tertiary nitrogen N−(C)$_3$ groups, and C–N–H bonds, respectively. The peaks at 532.3 eV and 534.1 eV in the O 1s spectrum are related to C–O and adsorbed $H_2O$, respectively.

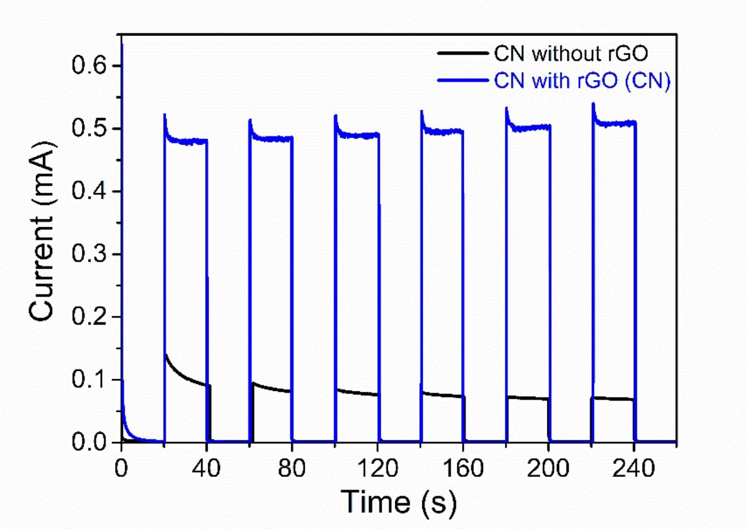


**Fig. S5.** Chronoamperometry (current *vs.* time) of CN electrode without (black) and with (blue) rGO in 1 M NaOH aqueous solution (aqueous solution, pH = 13.9). Geometric area of photoelectrode $A = 1.7$ cm × 2.0 cm = 3.4 $cm^2$.

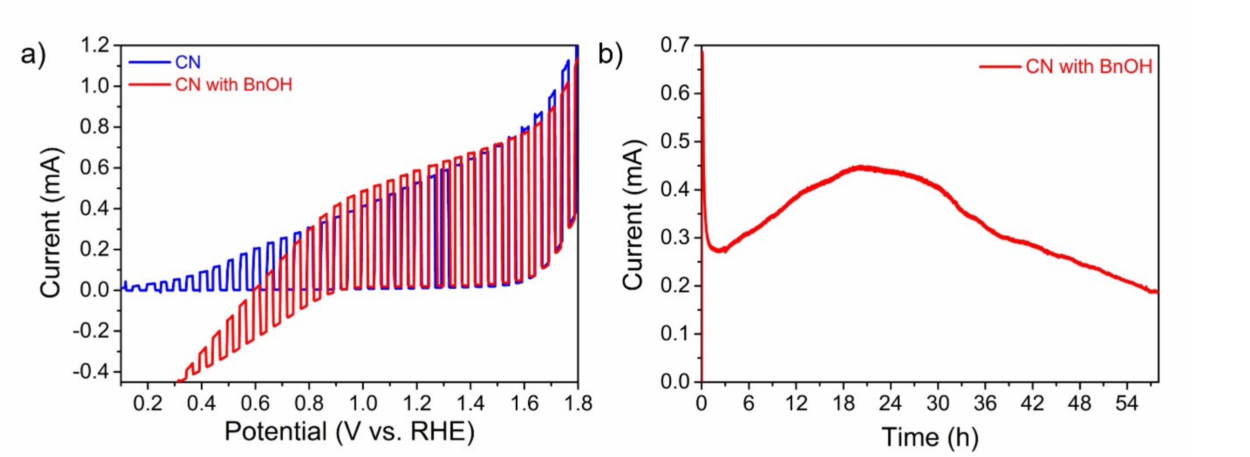


**Fig. S6. Linear sweep voltammetry (LSV) curves and chronoamperometric characterization of CN electrodes.** a) LSV curves of CN without (blue) and with (red) BnOH in the presence of $O_2$ under on/off illumination; scan rate of 5 mV $s^{-1}$, and b) chronoamperometry (photocurrent *vs.* time) for CN during the oxidation of BnOH at 1.22 V *vs.* RHE under simulated 1-sun illumination in the presence of $O_2$ (substrate concentration: 10 mM of BnOH in 1 M NaOH aqueous solution).

**Table S1.** BnOH oxidation using CN photoanode at different reaction times

| Time (h) | Charge (C) | Conversion (%) | BZD (%) | BZD (mmol) | BZA (%) | BZA (mmol) | BZD sel. (%) | BZA sel. (%) | Faradaic yield (%) |
|---|---|---|---|---|---|---|---|---|---|
| 6 | 5.8 | 19 | 19 | 0.034 | — | — | 100 | — | >99 |
| 12 | 10.6 | 32.3 | 32.3 | 0.058 | — | — | 100 | — | >99 |
| 24 | 30.7 | 67.4 | 65.9 | 0.12 | 1.5 | 0.003 | 98 | 2.2 | 78 |
| 36 | 54.4 | 89.8 | 76.6 | 0.14 | 13.2 | 0.024 | 85.4 | 14.6 | 66 |
| 48 | 62 | 100 | 12.9 | 0.023 | 87.1 | 0.16 | 12.9 | 87.1 | >99 |
| 58 | 69.3 | 100 | — | — | 99 | 0.18 | — | 100 | >99 |

Substrate concentration: 10 mM of BnOH (0.18 mmol) in 18 mL of 1 M NaOH aqueous solution in the presence of $O_2$. The reaction conversions and the amounts of oxidation product were determined by GC-MS. sel. stands for selectivity.

**Table S2.** BnOH oxidation using CN photoanode under different atmospheres

| Conditions | Photocurrent (mA) | Charge (C) | Conversion (%) | BZD (%) | BZD (mmol) | BZA (%) | BZA (mmol) | BZD sel. (%) | BZA sel. (%) | Faradaic yield (%) |
|---|---|---|---|---|---|---|---|---|---|---|
| Argon | ~0.15–0.1 | 25.2 | 39.3 | 34.2 | 0.062 | 5.1 | 0.009 | 87 | 13 | 61.2 |
| Air | ~0.35–0.23 | 49 | 80 | 55.6 | 0.1 | 24.5 | 0.044 | 69.4 | 30.6 | 74.2 |
| $O_2$ (PEC) | ~0.45–0.3 | 62 | 100 | 12.9 | 0.023 | 87.1 | 0.16 | 12.9 | 87.1 | >99 |

Substrate concentration: 10 mM of BnOH (0.18 mmol) in 18 mL of 1 M NaOH aqueous solution. Reaction time is 48 h. The reaction conversions and the amounts of oxidation product were determined by GC-MS. sel. stands for selectivity.

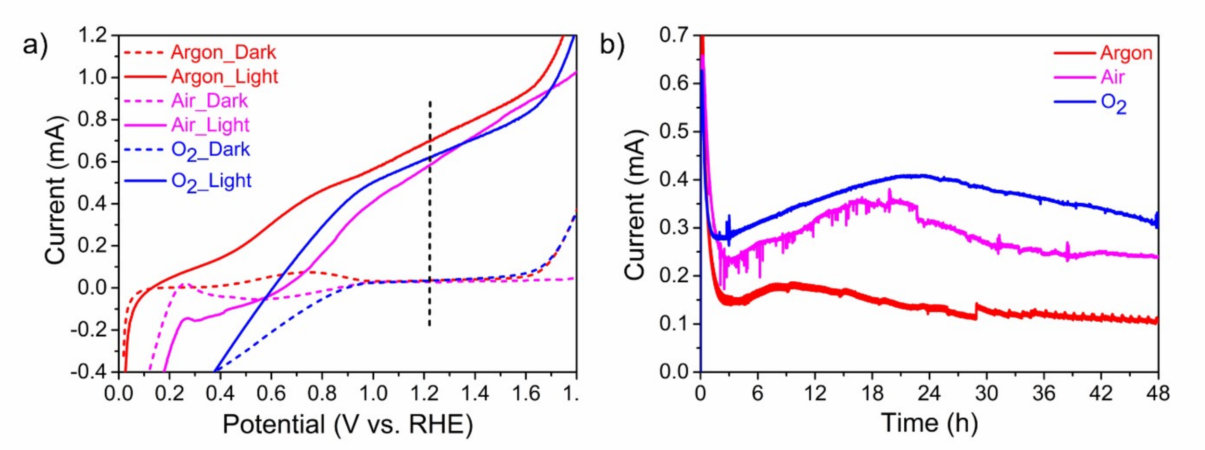


**Fig. S7. Linear sweep voltammetry (LSV) curves and chronoamperometric characterization of CN electrodes.** a) LSV curves of CN with BnOH in the presence of argon, air, and $O_2$ under illumination (complete curves) and in the dark (dashed), and b) chronoamperometry (photocurrent *vs.* time) for CN during the oxidation of BnOH at 1.22 V *vs.* RHE under simulated 1-sun illumination in the presence of argon, air, and $O_2$ for 48 h (substrate concentration: 10 mM of BnOH in 1 M NaOH aqueous solution).

**Table S3.** PEC BnOH oxidation using carbon nitride electrodes at different conditions

| Conditions | Photocurrent (mA) | Charge (C) | Conversion (%) | BZD (%) | BZD (mmol) | BZA (%) | BZA (mmol) | Faradaic yield (%) |
|---|---|---|---|---|---|---|---|---|
| $O_2$ (PEC)[a] | ~0.45–0.3 | 62 | 100 | 12.9 | 0.023 | 87.1 | 0.16 | >99 |
| Electrocatalysis[a] | — | 0.16 | 1 | 1 | 0.002 | — | — | — |
| Photocatalysis[a] | — | — | 4 | 4 | 0.007 | — | — | — |
| pH 7[b] | ~0.1–0.03 | 11.5 | 31.2 | 31.2 | 0.056 | — | — | 94.3 |
| pH 10[a] | ~0.15–0.1 | 25.2 | 36.4 | 33.6 | 0.061 | 2.8 | 0.005 | 54.1 |

Substrate concentration: 10 mM of BnOH (0.18 mmol) in 18 mL of 1 M NaOH solution.
[a]Reaction time is 48 h. For the pH 7 and the pH 10 solutions, 0.1 M phosphate buffer and 0.5 M borate buffer solutions were used, respectively.
[b]The reaction was stopped after 38 h in pH 7 because of a continuous drop in the photocurrent. The reaction conversions and the amount of oxidation products were determined by GC-MS.

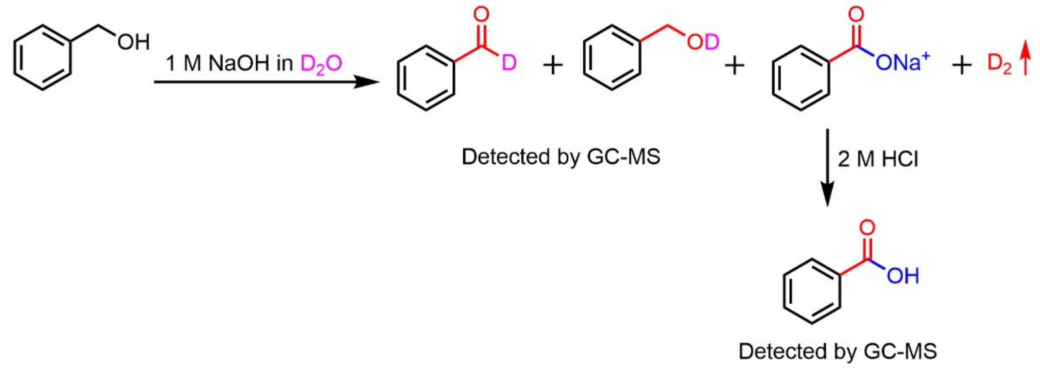


**Fig. S8.** PEC oxidation reaction of BnOH using an aqueous solution containing 1 M NaOH prepared in $D_2O$ ($H_2O$ is replaced by $D_2O$).

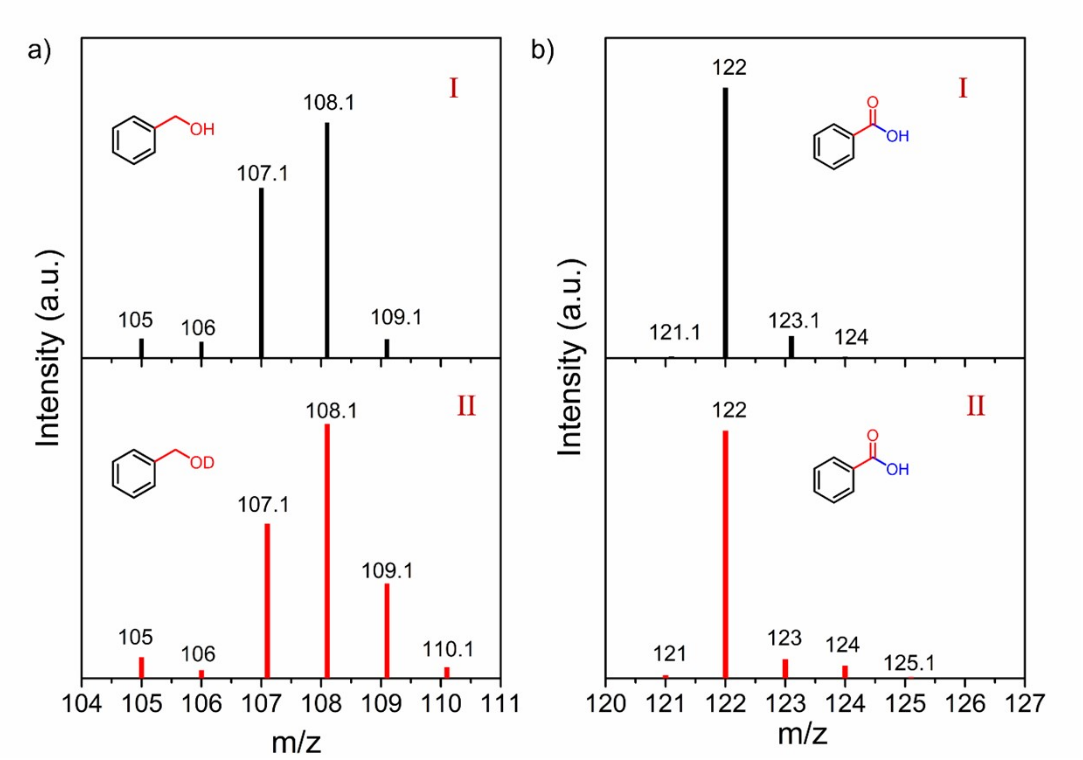


**Fig. S9. Labeling experiments in $D_2O$.** a) Mass spectra of BnOH in a $H_2O$ (I) and $D_2O$ (II) solution containing 1 M NaOH, and b) Mass spectra of BZA produced in a $H_2O$ (I) and $D_2O$ (II) solution containing 1 M NaOH.

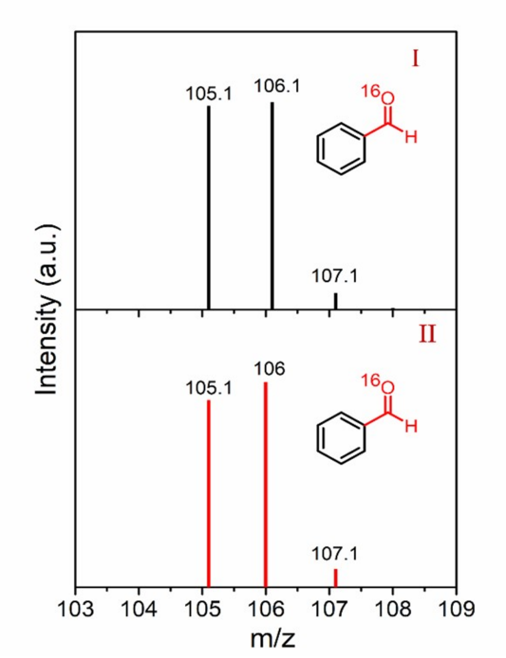


**Fig. S10. Labeling experiments with $^{18}O_2$ (labeled oxygen gas).** Mass spectra of benzaldehyde produced in $^{16}O_2$ (I) and $^{18}O_2$, (II) showing the absence of labeling in benzaldehyde.

**Table S4.** PEC BnOH oxidation reaction over CN photoanode in the presence of different radical scavengers

| Conditions | Photocurrent density (mA) | Charge (C) | Conversion (%) | BZD (%) | BZD (mmol) | BZA (%) | BZA (mmol) | BZD sel. (%) | BZA sel. (%) | Faradaic yield (%) |
|---|---|---|---|---|---|---|---|---|---|---|
| $O_2$ (PEC) | ~0.45–0.35 | 62 | 100 | 12.9 | 0.023 | 87.1 | 0.16 | 12.9 | 87.1 | >99 |
| BQ | ~0.4–0.3 | 60 | 94 | 52 | 0.093 | 42 | 0.076 | 55.3 | 44.7 | 79 |
| TBA | ~0.38–0.2 | 59 | 99 | 19 | 0.034 | 80 | 0.144 | 19.2 | 80.8 | >99 |
| $K_2S_2O_8$ | ~0.2–0.05 | 30 | 86 | 78 | 0.140 | 8 | 0.014 | 91 | 9.3 | >99 |
| TEOA | ~0.25–0.12 | 42.8 | — | — | — | — | — | — | — | — |

Substrate concentration: 10 mM of alcohol (0.18 mmol) in 1 M NaOH aqueous solution (pH 13.9). Reaction time is 48 h. BQ as a $\bullet O_2^-$ radical scavenger, 0.5 mM; TBA as a •OH radical scavenger, 1 mM; $K_2S_2O_8$ as an $e^-$ scavenger, 1 mM; TEOA as a $h^+$ scavenger, 10% v/v). The reaction conversions and the amounts of oxidation product were determined by GC-MS.

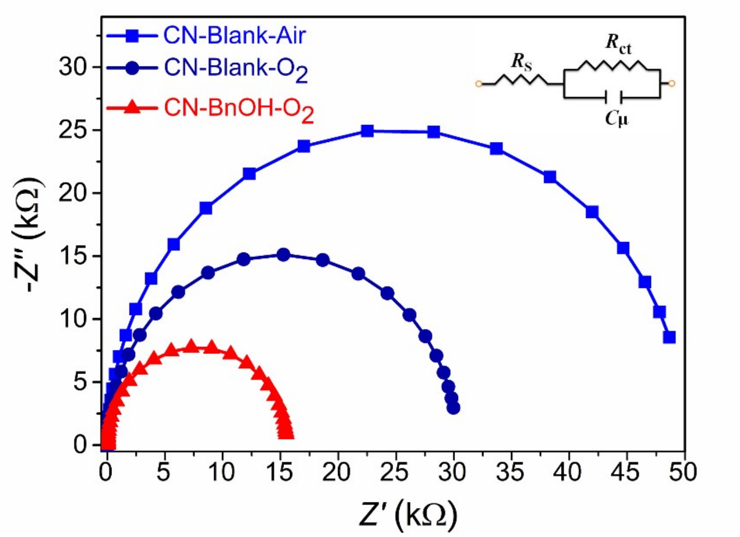


**Fig. S11.** Nyquist plots of CN at 1.22 V *vs.* RHE showing the plot for a CN electrode in air and $O_2$, and CN with BnOH in $O_2$ (inset: the electrochemical equivalent circuit used for fitting).

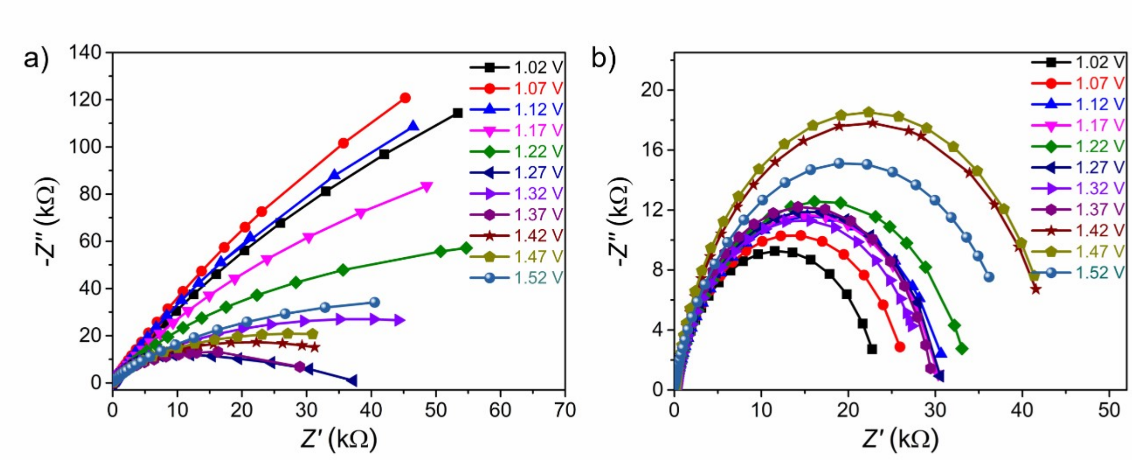


**Fig. S12. Nyquist plots of CN electrodes.** a) CN in aqueous solution (1 M NaOH, pH 13.9), and b) CN in an aqueous solution (1 M NaOH, pH 13.9) containing BnOH. Both (a) and (b) were obtained in the presence of $O_2$ and at various applied voltage biases. The charge transfer resistance ($R_{ct}$) was extracted by fitting the semicircles of the Nyquist plots using the equivalent circuit shown in the inset of Fig. S11.

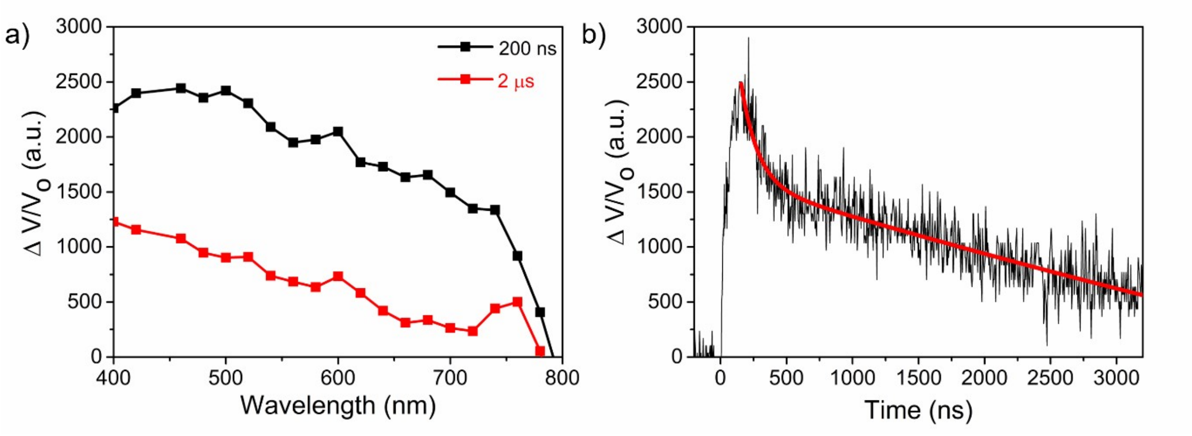


**Fig. S13.** TA of CN dispersion in acetonitrile under $N_2$ atmosphere (monitored at 500 nm) at a) different acquisition times, and b) kinetics. Laser excitation: 355 nm.

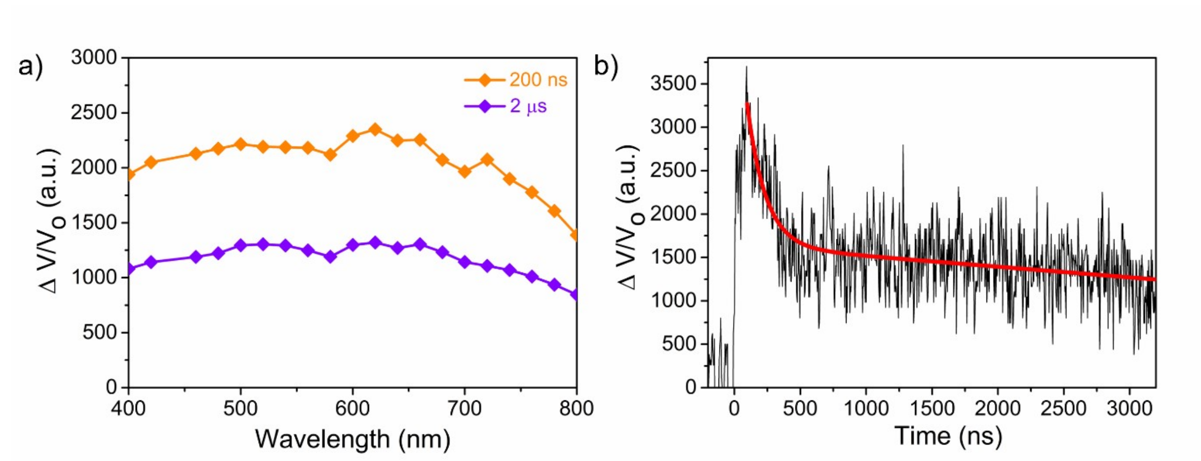


**Fig. S14.** TA of CN dispersion in acetonitrile under $O_2$-saturated atmosphere (monitored at 660 nm) at a) different acquisition times and, b) kinetics. Laser excitation: 355 nm.

Transient absorption (TA) measurements have been performed in a CN dispersion in acetonitrile upon 355 nm laser excitation.[2] First, the TA spectrum of CN was acquired at different time delays after the laser pulse under $N_2$ atmosphere (Fig. S13). This spectrum shows a continuous absorption decreasing in intensity from the visible to the NIR. CN TA decay was monitored at 500 nm, and the experimental data show a bimodal behavior that can be fitted to two consecutive first-order kinetics (Equation 1, $F(t) = a_1 e^{-\tau_1 t} + a_2 e^{\tau_2 t}$). The fast and slow lifetime components were calculated from the data fit to Equation 1, resulting in 127.9 ns and 15.4 μs, respectively. Then, the CN dispersion was $O_2$ saturated, and the TA spectrum and kinetics measured (Fig. S14). The CN TA spectrum under $O_2$-saturated atmosphere shows a continuous band along the measured

spectrum, exhibiting a relative maximum intensity around 660 nm. The CN TA kinetics at 500 and 660 nm were coincident, indicating that they correspond to a single species. In this case, the TA kinetics also corresponds to a bimodal behavior, and, after experimental data fitting to Equation 1, the lifetime of the fast component was 168.5 ns, very similar than that obtained under $N_2$ atmosphere. However, the lifetime of the slowest component was 18.9 ms—3 orders of magnitude longer than that in $N_2$. $O_2$ is considered an efficient $e^-$ acceptor, and therefore, the photogenerated $e^-$ in CN must be efficiently quenched.[3] As consequence, the obtained CN TA under $O_2$ atmosphere can be attributed to photogenerated $h^+$ in the CN valence band (VB). Moreover, from the TA kinetics under $N_2$ and $O_2$ atmosphere, it can be determined that the detected transient species in the ns time scale are not contributing to the photo-induced $e^-$ transfer from CN to $O_2$, since both decays show similar lifetimes. However, the slow component in the TA of CN in the presence of $O_2$ can be assigned to long lived photogenerated $h^+$. These observations are in good agreement with the previous control experiments in which the optimal BnOH conversion is obtained under $O_2$-saturated atmosphere, and long-lived $h^+$ in the photoanode can play a key role in the reaction mechanism.

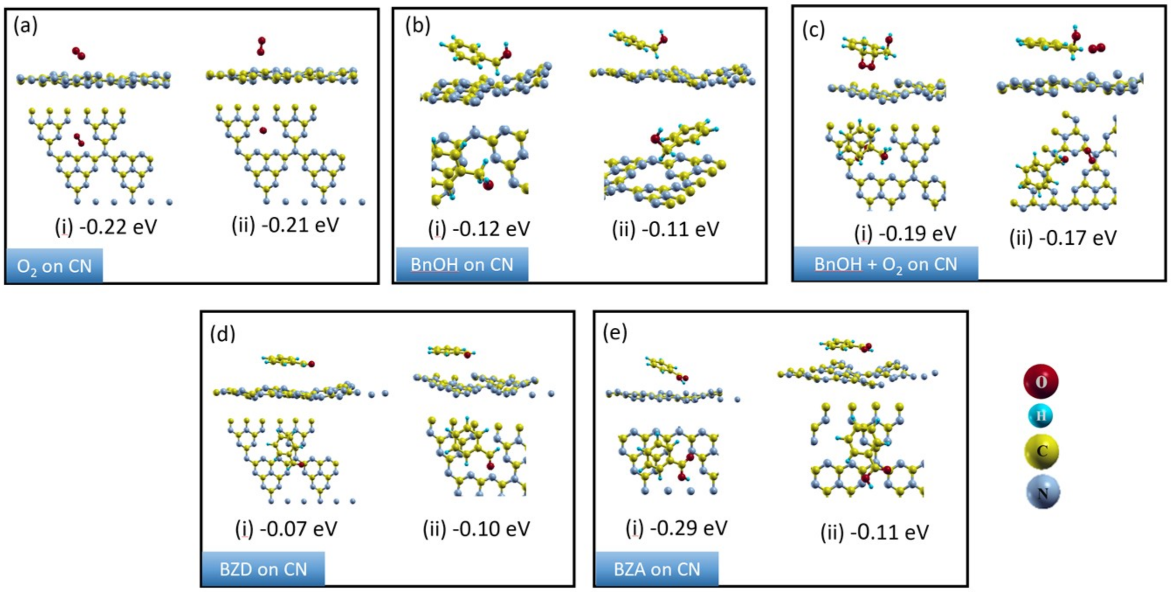


**Fig. S15.** The favorable configuration of a) molecular $O_2$, b) BnOH, c) BnOH + $O_2$, d) BZD, and e) BZA over CN. The adsorption energy beneath each illustration was computed relative to the energy sum of the relaxed CN and the isolated relaxed moiety.

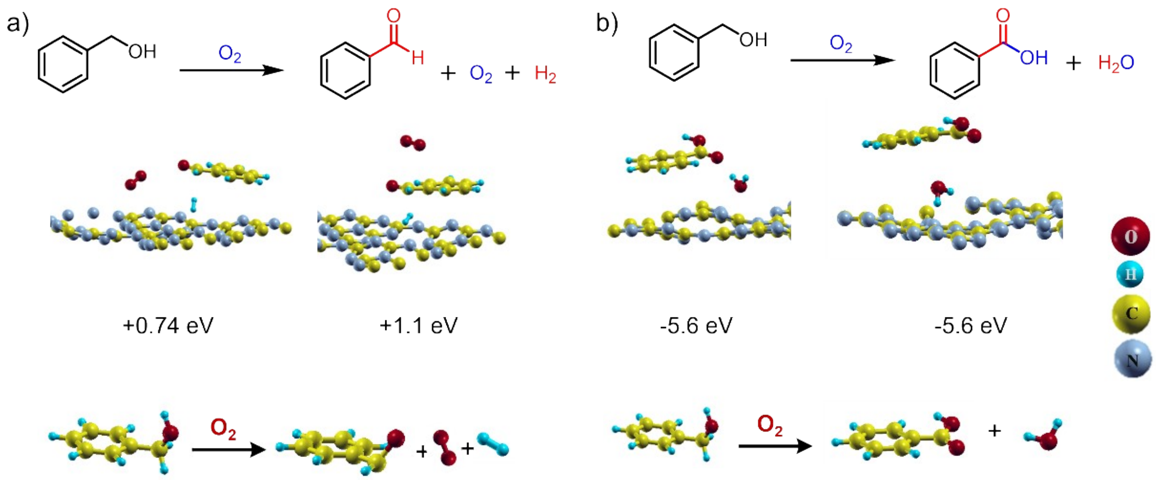


**Fig. S16.** The favorable configuration of two types of BnOH fragments with $O_2$ + CN: a) BZD + $H_2$ + $O_2$ and b) BZA + $H_2O$. The energy beneath each illustration is similar to the dissociation enthalpy of the benzyl alcohol into BZD and molecular hydrogen and similar to the reaction enthalpy for the oxidation of the benzyl alcohol into BZA and the hydrogen into $H_2O$.

The $\bullet O_2^-$ radicals may react with BnOH on the CN surface to provide BZD. As can be seen from the calculation in Fig. S16a, the enthalpy to form the BZD from $O_2$ and BnOH shows that the reaction is not favorable. Thus, the formation of BZD requires an energy input. The PEC oxidation process can be the energy source that produces the $\bullet O_2^-$ radicals and therefore helps the BZD formation. Indeed, as observed experimentally, in the vicinity of adsorbed $O_2$, BnOH dissociates into BZD and molecular hydrogen ($H_2$). When this reaction takes place, BZD can be easily desorbed from the CN photoanode surface thanks to its low adsorption energy (see Fig. S15d), which is similar to that of BnOH (Fig. S15b); this easy desorption keeps the electrode available for the production of another BZD.

From the thermodynamic viewpoint, these BZD moieties can react with the adsorbed oxygen to produce BZA. Indeed, as observed experimentally, the production of BZA moieties follows the production of BZD. This reaction has a thermodynamic drive (Fig. S16b) and may occur more easily. Similarly, the adsorbed energy of the BZA is relatively small and therefore can be easily desorbed from the CN photoanode, making it available to produce another BZA (Fig. S15e).

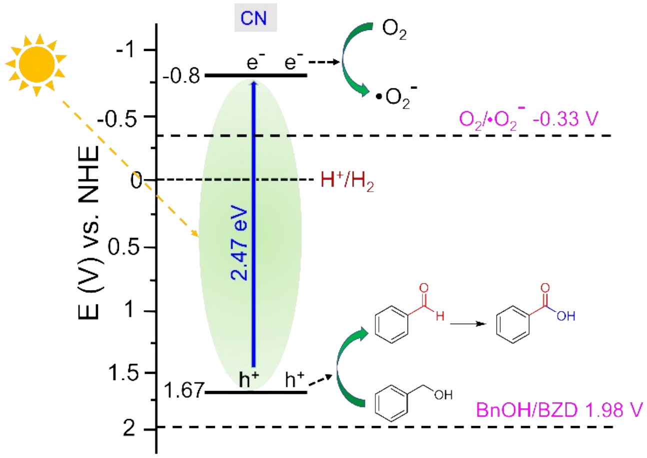


**Fig. S17.** Energy level diagram for the PEC oxidation of benzyl alcohol (BnOH) to benzaldehyde (BZD) and further to benzoic acid (BZA) over CN under visible-light irradiation. The valence band (VB) energy level of the CN electrode was determined from the optical $E_g$ and the Mott-Schottky analysis in Fig. S3.

**Table S5**. Scope of substrates for the formation of carboxylic acids.[a]

| Substrate | Charge (C) | Photocurrent (mA) | Time (h) | Conversion (%)[b] | Yield (%)[c] | Product (mmol) | Faradaic yield (%) |
|---|---|---|---|---|---|---|---|
| Benzyl alcohol | 69.3 | ~0.45–0.3 | 58 | 100 | 99 | 0.18 | 99.3 |
| 4-methylbenzyl alcohol | 69.6 | ~0.45–0.3 | 62 | 100 | 97 | 0.17 | 97.0 |
| 4-methoxybenzyl alcohol[d] | 69.2 | ~0.4–0.3 | 69 | 100 | 62 | 0.11 | 81.3 |
| 4-(trifluoromethyl)benzyl alcohol | 70 | ~0.45–0.3 | 60 | 100 | 99 | 0.18 | 98.3 |
| 4-chlorobenzyl alcohol | 69.7 | ~0.45–0.35 | 56 | 100 | 99 | 0.18 | 98.7 |
| 4-nitrobenzyl alcohol | 69.2 | ~0.45–0.3 | 60 | 100 | 99 | 0.18 | 99.4 |
| 2-pyridine methanol | 70.2 | ~0.35–0.25 | 72 | 100 | 93 | 0.17 | 92.1 |
| 3-pyridine methanol | 70 | ~0.4–0.25 | 70 | 100 | 98 | 0.18 | 97.3 |
| 4-pyridine methanol | 70.1 | ~0.4–0.25 | 70 | 100 | 97 | 0.17 | 96.0 |

[a]Conditions: 10 mM alcohol (0.18 mmol) in 18 mL of 1 M NaOH aqueous solution (pH 13.9). Photoelectrolysis was conducted in constant potential mode (1.22 V *vs*. RHE) under 1-sun illumination.

[b]The reaction conversions (in parentheses) were determined by gas chromatography (GC-MS) and $^{1}H$ NMR.

[c]The amounts of oxidation product (**3**) were determined by $^{1}H$ NMR by using dibromomethane as an internal standard.

[d]The oxidation of 4-methoxybenzyl alcohol yielded 38% 4-methoxybenzaldehyde.

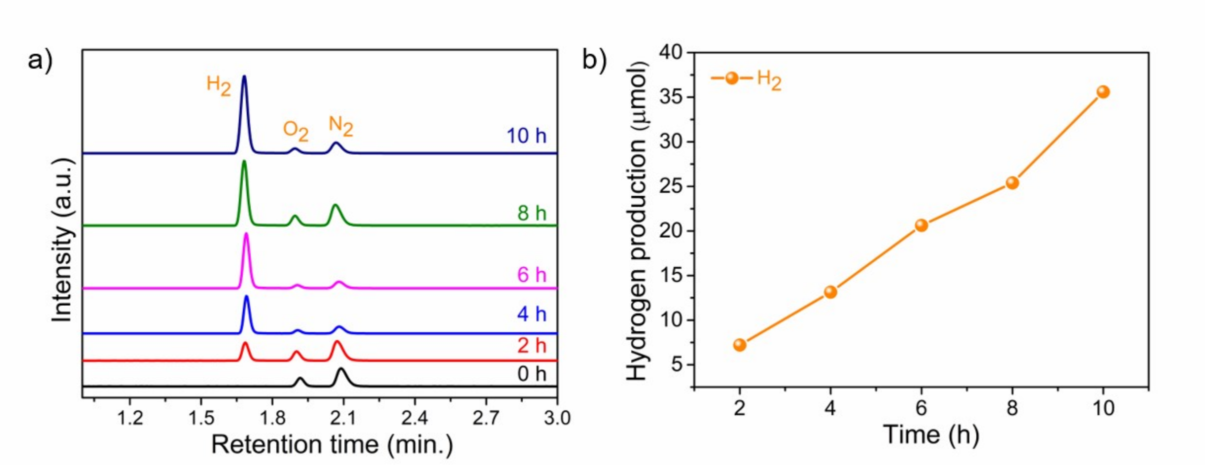


**Fig. S18. HER quantification.** a) Gas chromatographs for $H_2$ production at different reaction times during BnOH oxidation. The electrodes are biased at 1.22 V *vs.* RHE in a two-compartment cell. Substrate concentration: 10 mM of BnOH in 1 M NaOH aqueous solution, and b) Total $H_2$ production as a function of time during the BnOH oxidation reaction over a CN photoanode.

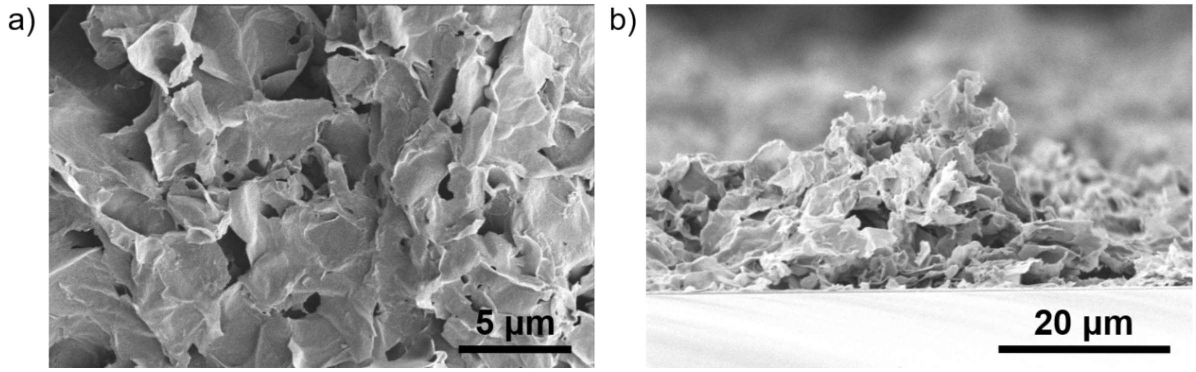


**Fig. S19. SEM characterization of CN electrodes after PEC oxidation of BnOH (24 h).** a) Top view, and b) cross section (thickness of CN layer above FTO ~18 μm).

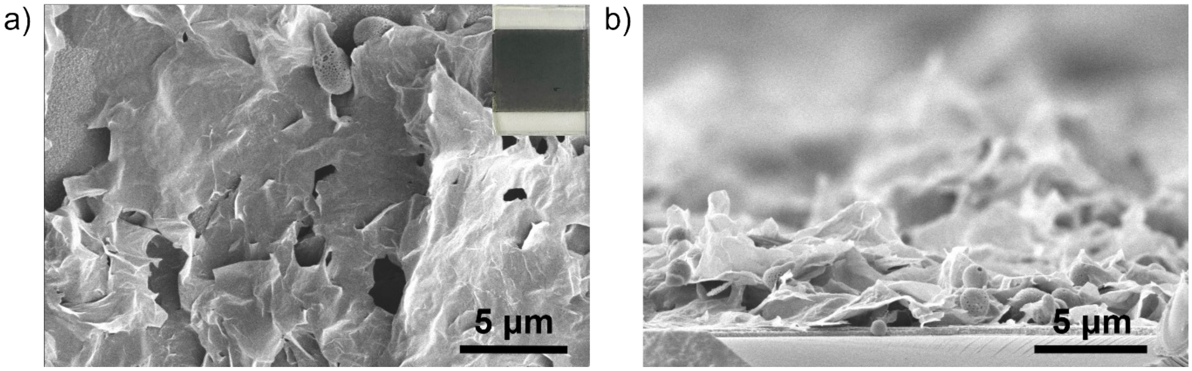


**Fig. S20. SEM characterization of CN electrodes after PEC oxidation of BnOH (48 h).** a) Top view (inset: digital image of the CN electrode after PEC), and b) cross section (thickness of CN layer above FTO ~5 µm).

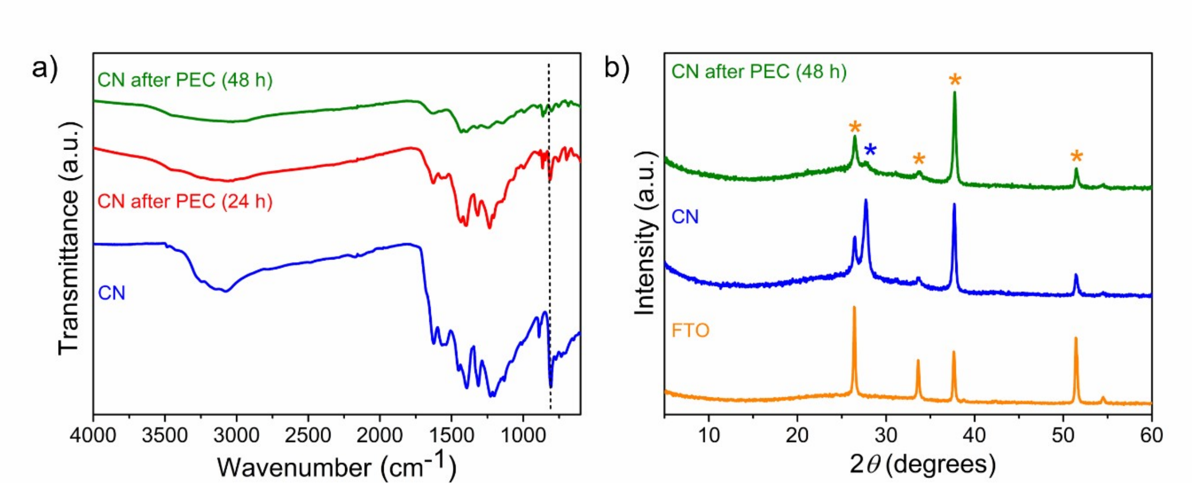


**Fig. S21. FTIR and XRD analysis of photoanodes before and after reaction.** a) FTIR spectra of CN films before and after PEC BnOH oxidation, and b) XRD patterns of CN films (over FTO) before and after PEC BnOH oxidation.

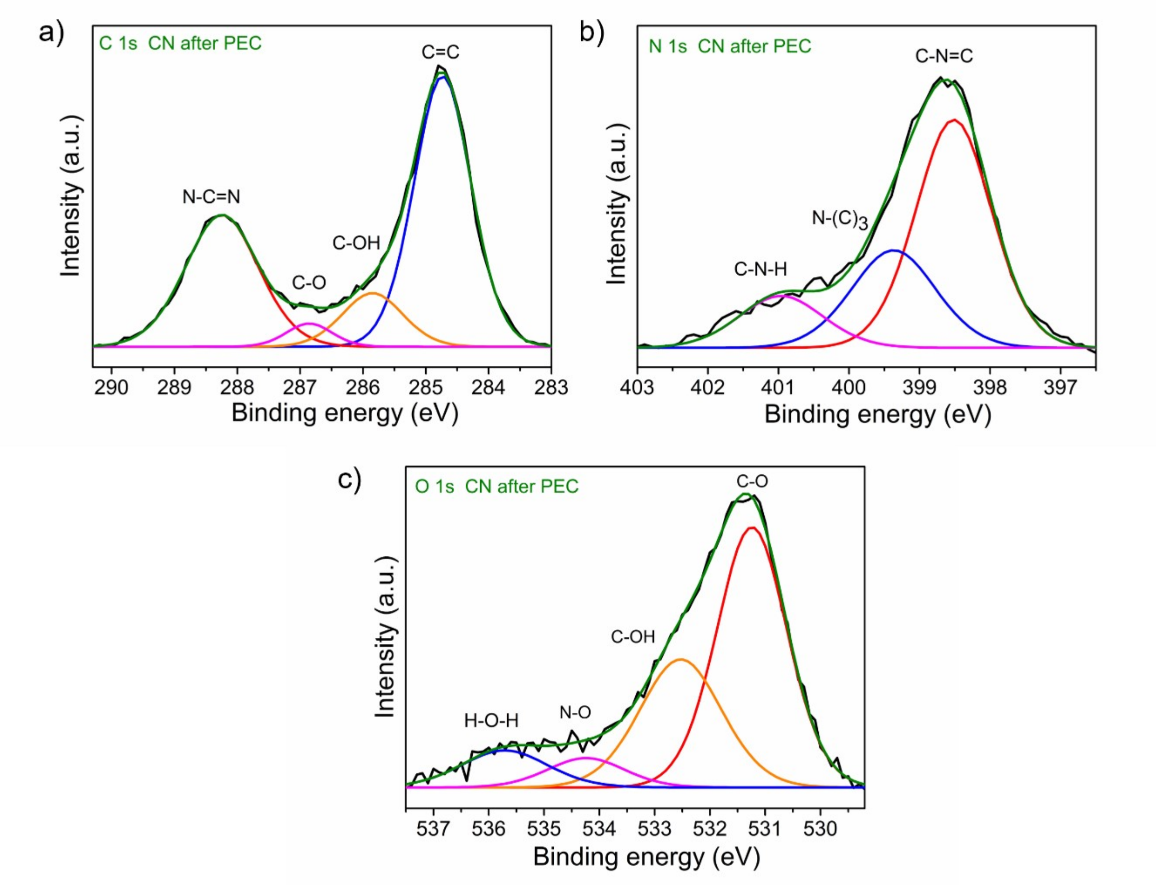


**Fig. S22. High-resolution XPS spectra of CN electrodes after a PEC oxidation reaction.** a) C 1s, b) N 1s, and c) O 1s. Experimental spectra are plotted in black; green lines represent the fitted spectra; magenta, blue, red, and orange are the individual deconvoluted fitted peaks.

**Table S6.** Elemental analysis of CN and after PEC organic transformations (48 h); obtained by XPS.

| Sample name | C (at.%) | N (at.%) | O (at.%) | C/N |
|---|---|---|---|---|
| CN | 45.83 (1s) | 52.89 (1s) | 1.28 (1s) | 0.87 |
| CN after PEC | 59.11(1s) | 27.80 (1s) | 13.10 (1s) | 2.13 |

**GC-MS spectra of the products**

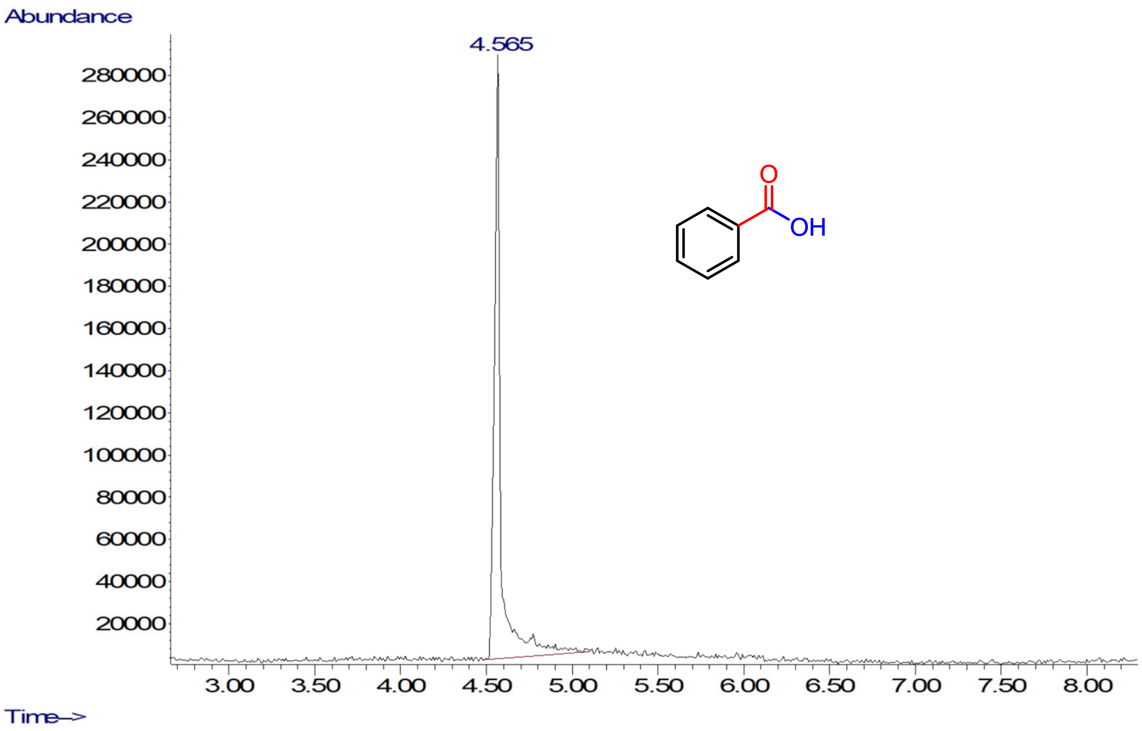


**Fig. S23a.** GC-MS trace of the reaction mixture after the oxidation of benzyl alcohol.

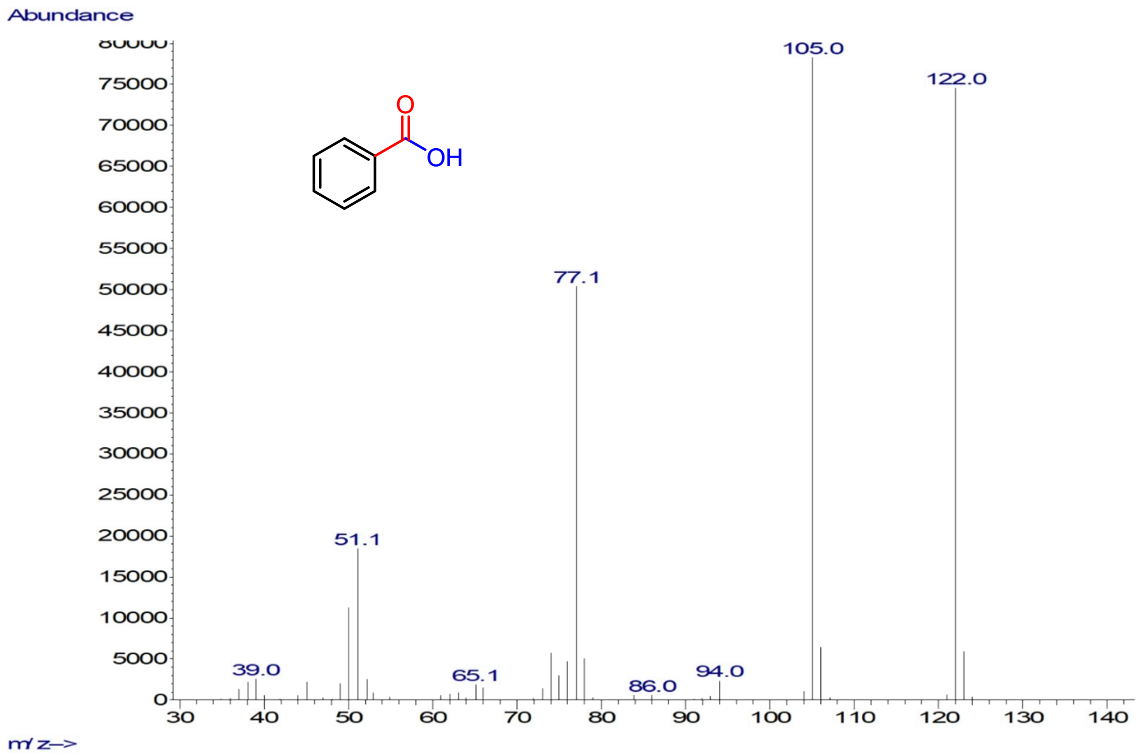


**Fig. S23b.** Mass spectra corresponding to the GC trace at 4.565 min.

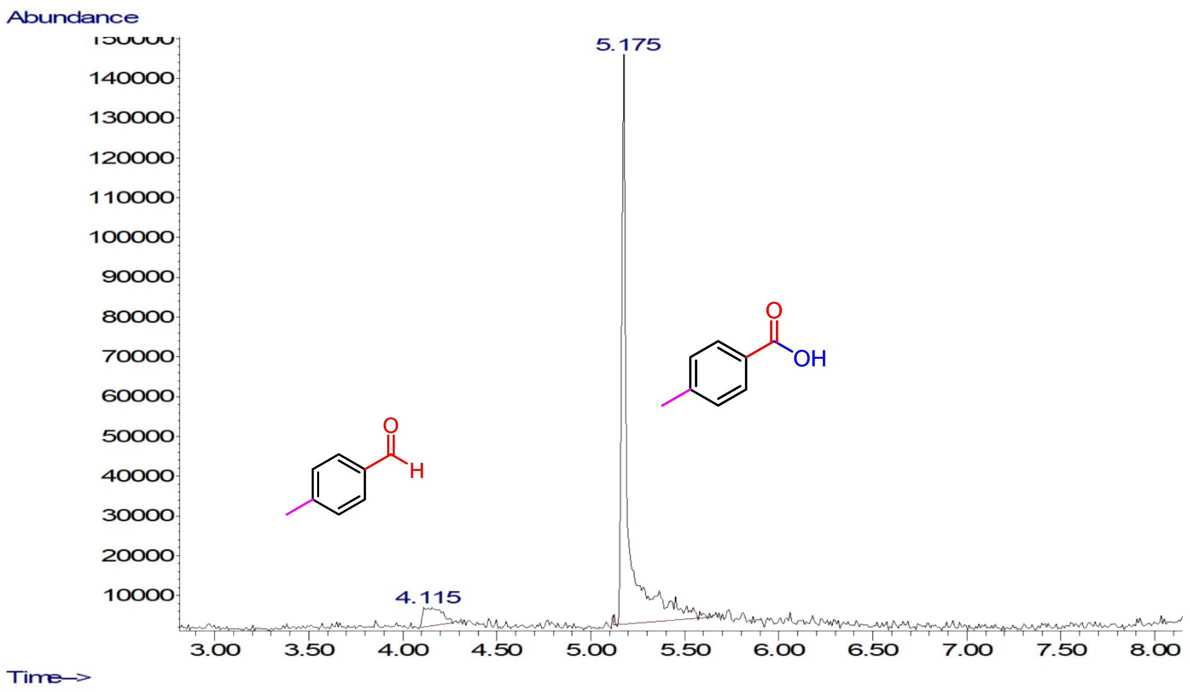


**Fig. S24a**. GC-MS trace of the reaction mixture after the oxidation of 4-methylbenzyl alcohol.

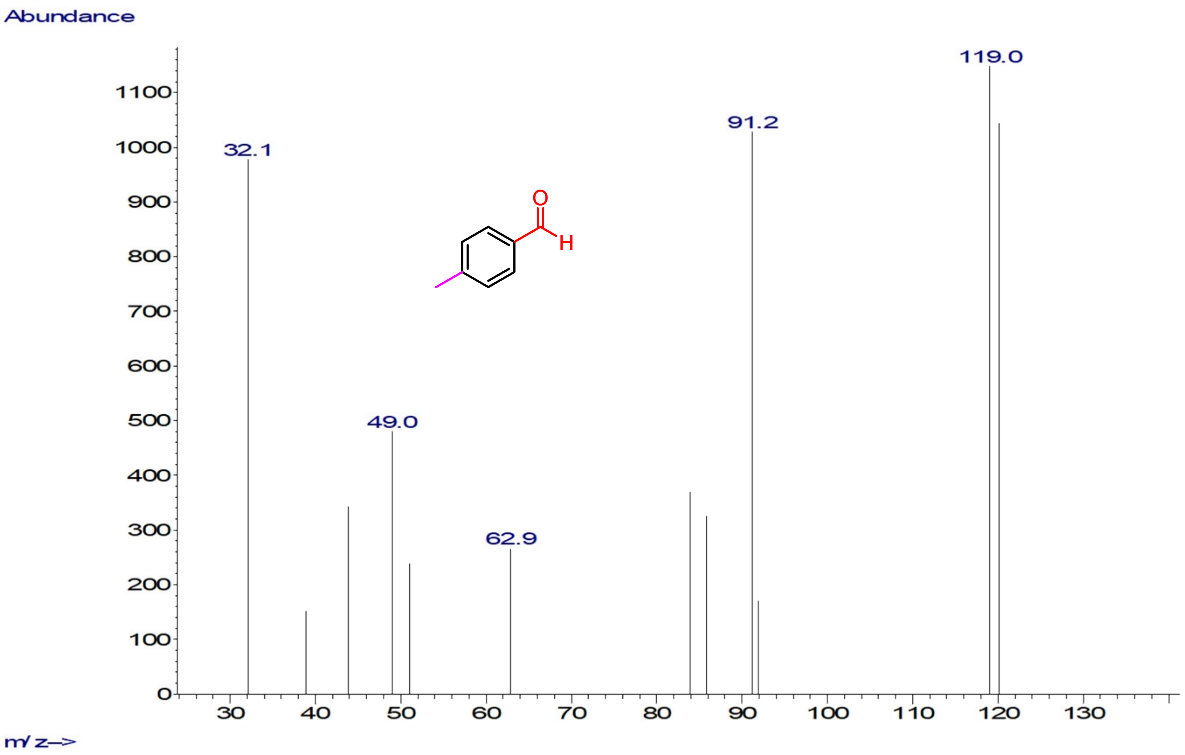


**Fig. S24b.** Mass spectra corresponding to the GC trace at 4.115 min.

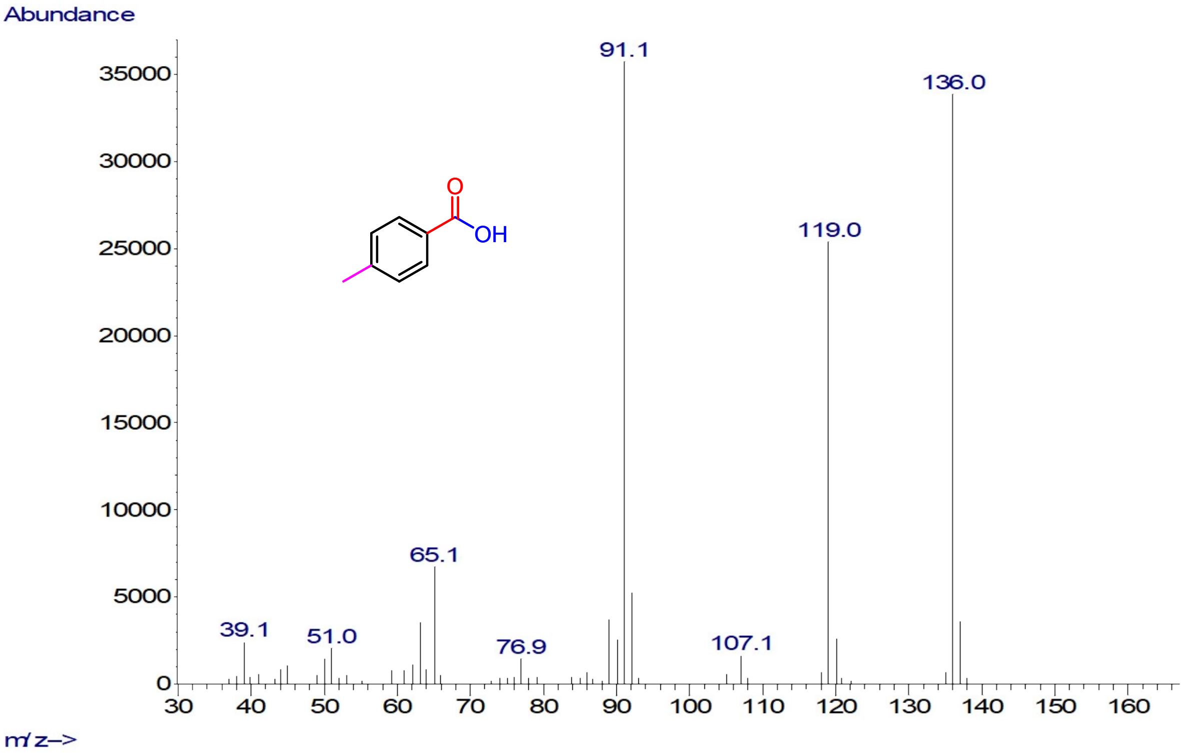


**Fig. S24c.** Mass spectra corresponding to the GC trace at 5.175 min.

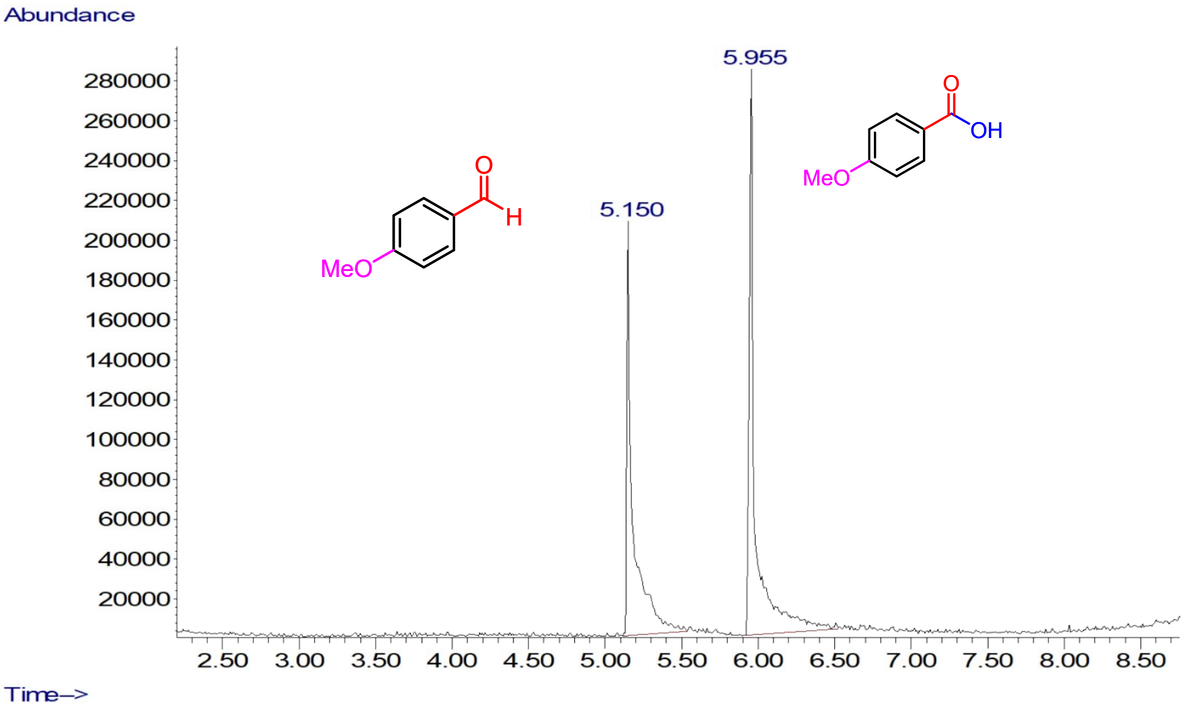


**Fig. S25a.** GC-MS trace of the reaction mixture after the oxidation of 4-methoxybenzyl alcohol.

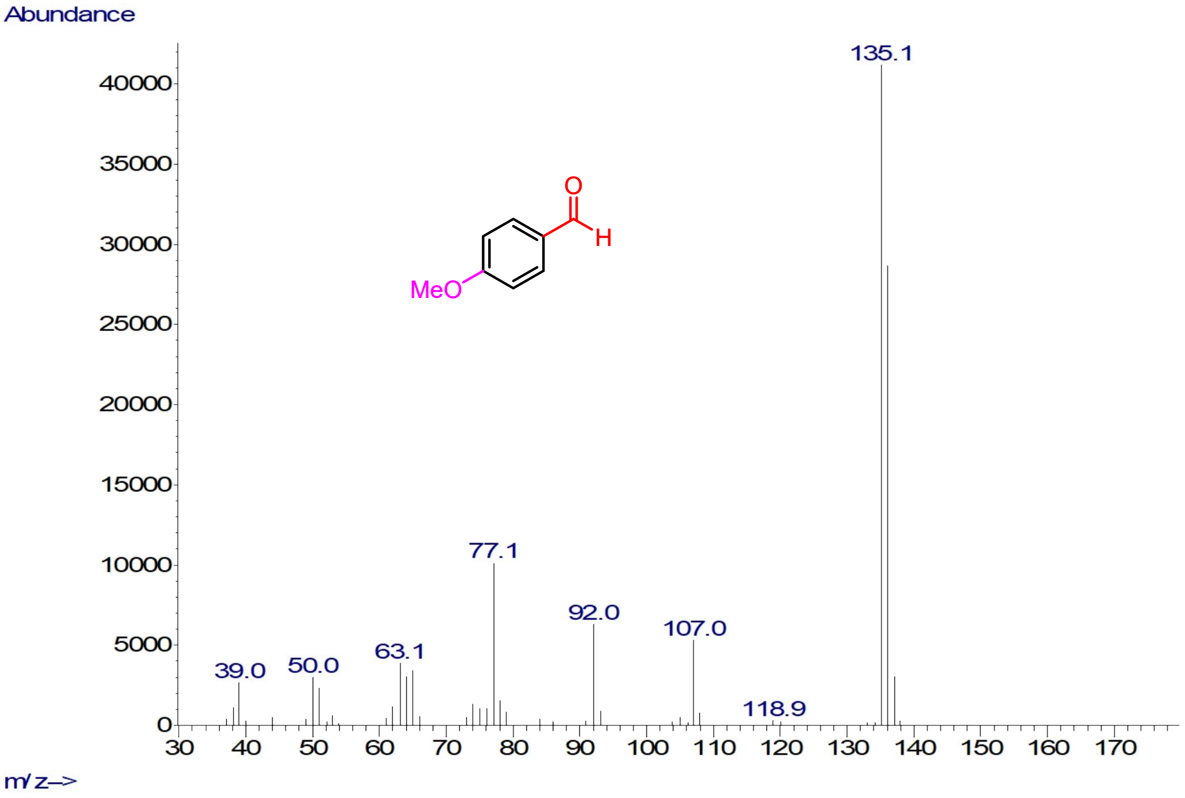


**Fig. S25b.** Mass spectra corresponding to the GC trace at 5.150 min.

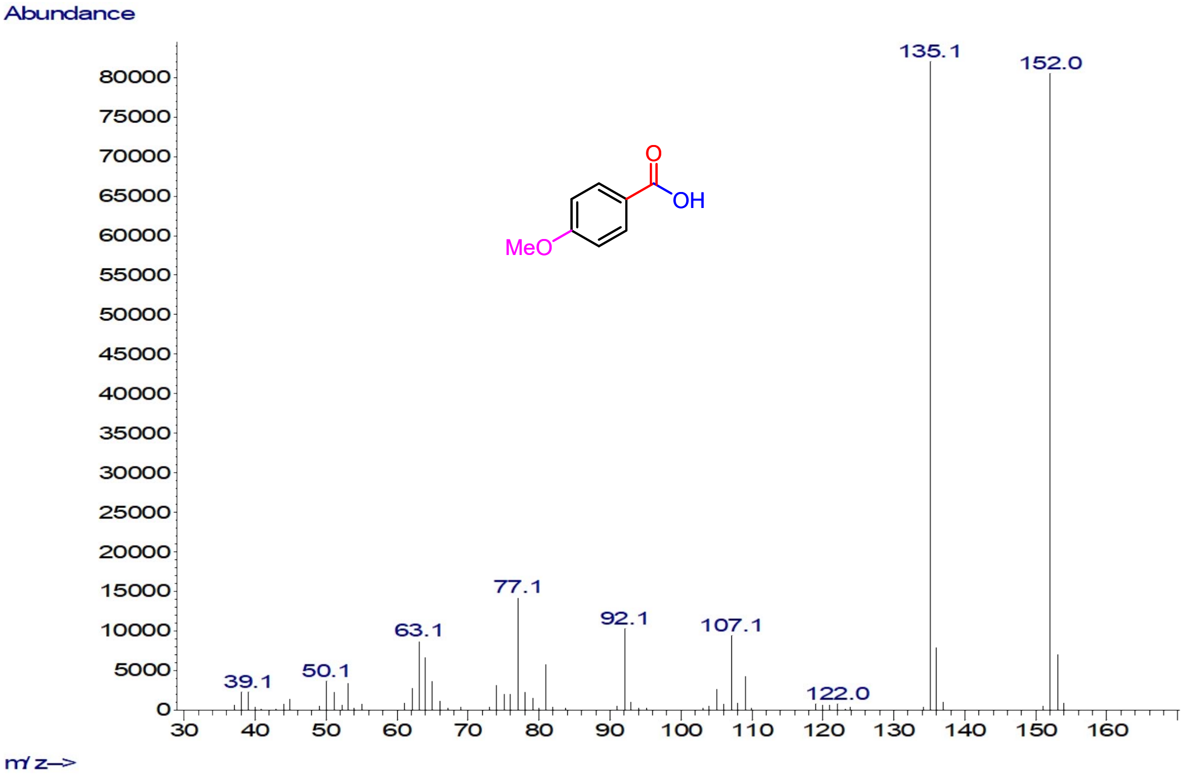


**Fig. S25c.** Mass spectra corresponding to the GC trace at 5.955 min.

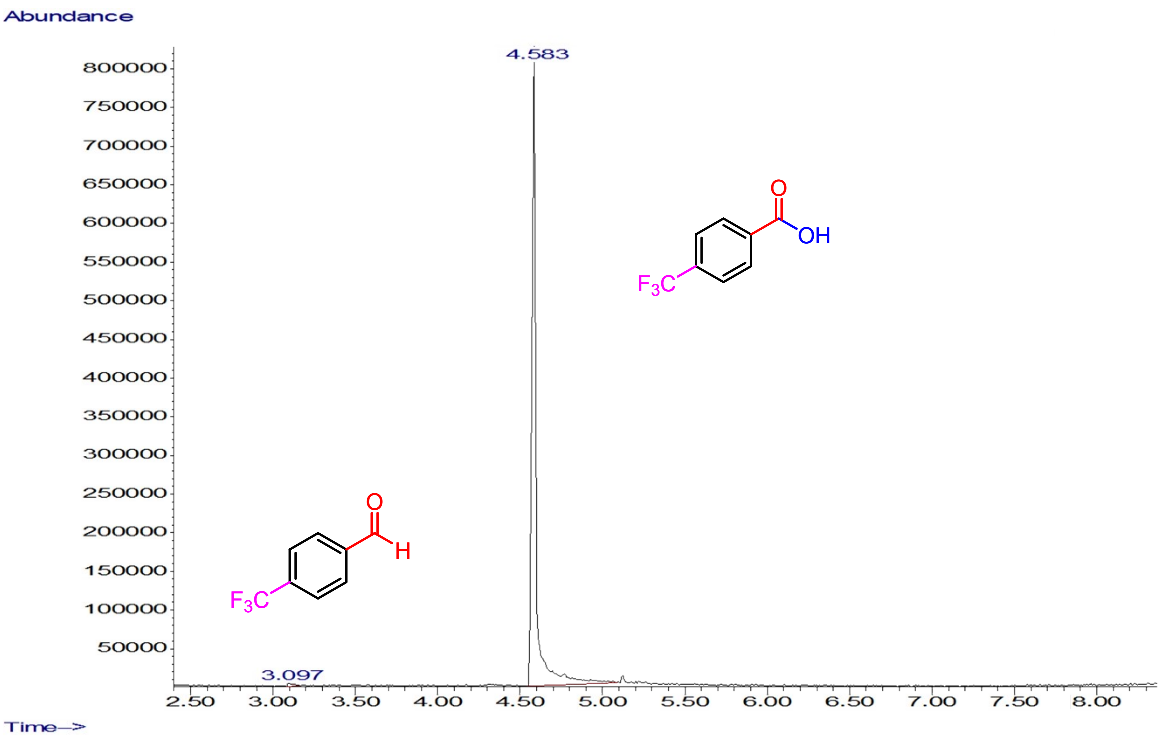


**Fig. S26a.** GC-MS trace of reaction mixture after the oxidation of 4-(trifluoromethyl)benzyl alcohol.

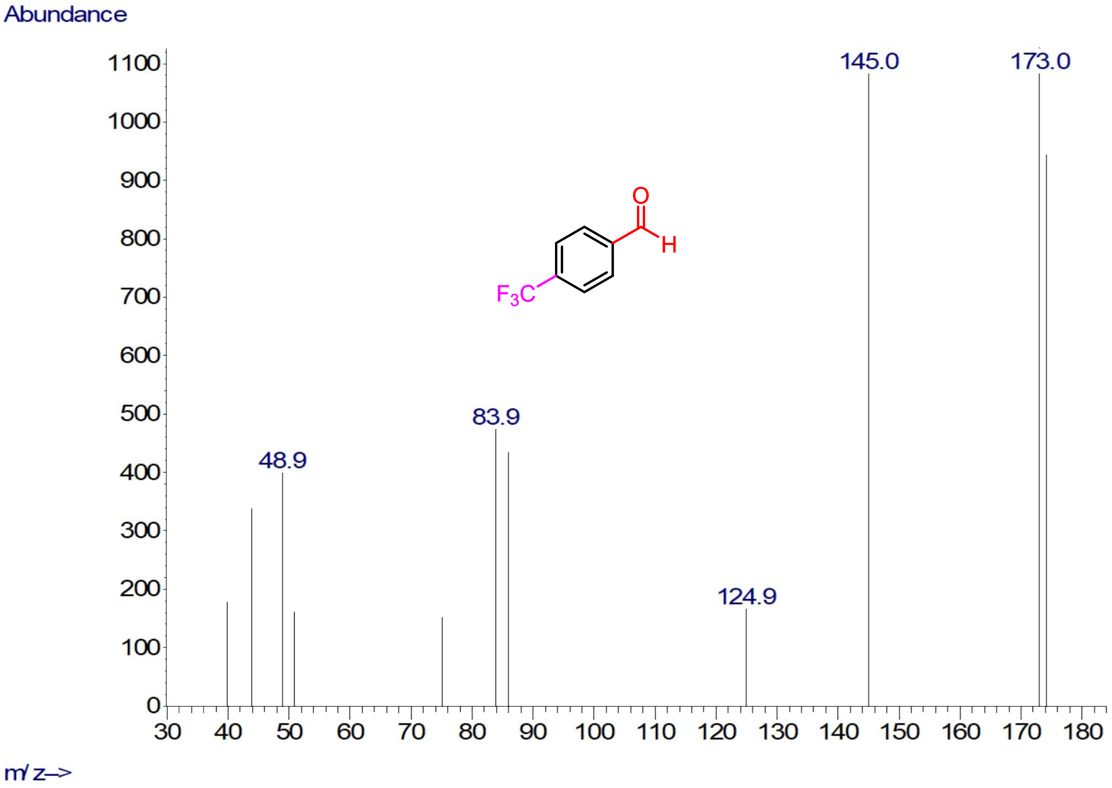


**Fig. S26b.** Mass spectra corresponding to the GC trace at 3.097 min.

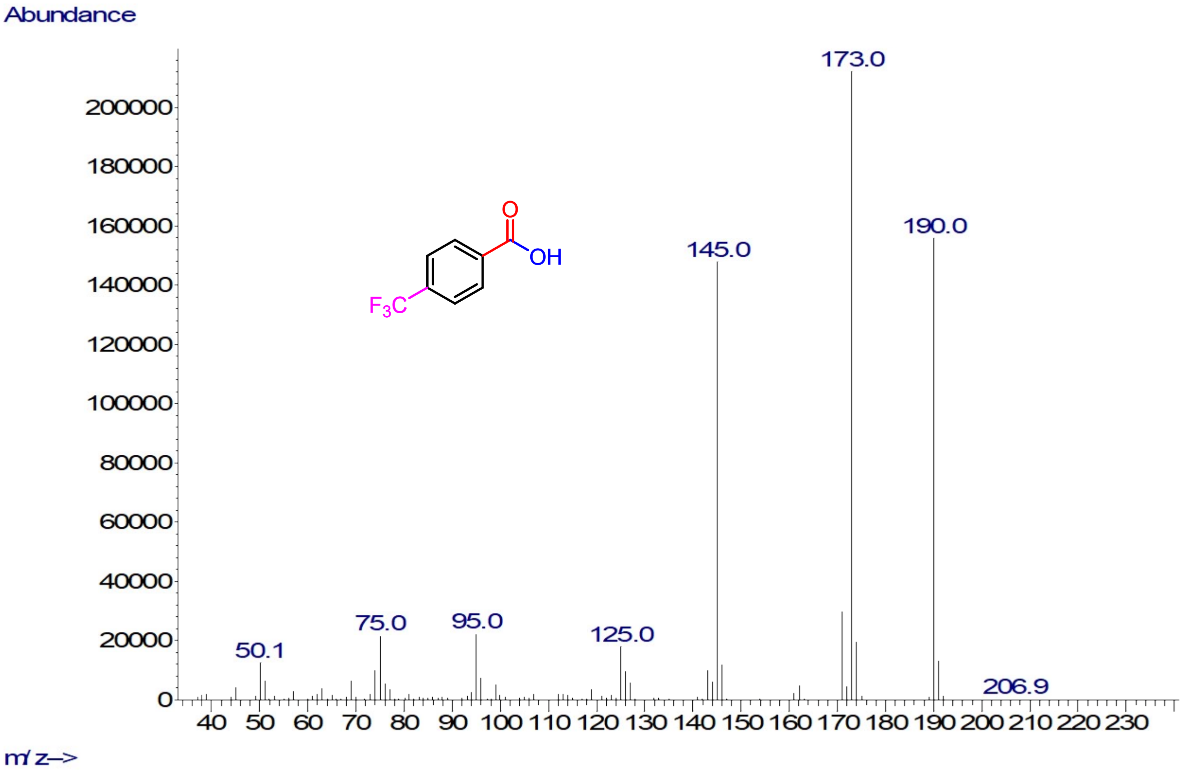


**Fig. S26c.** Mass spectra corresponding to the GC trace at 4.584 min.

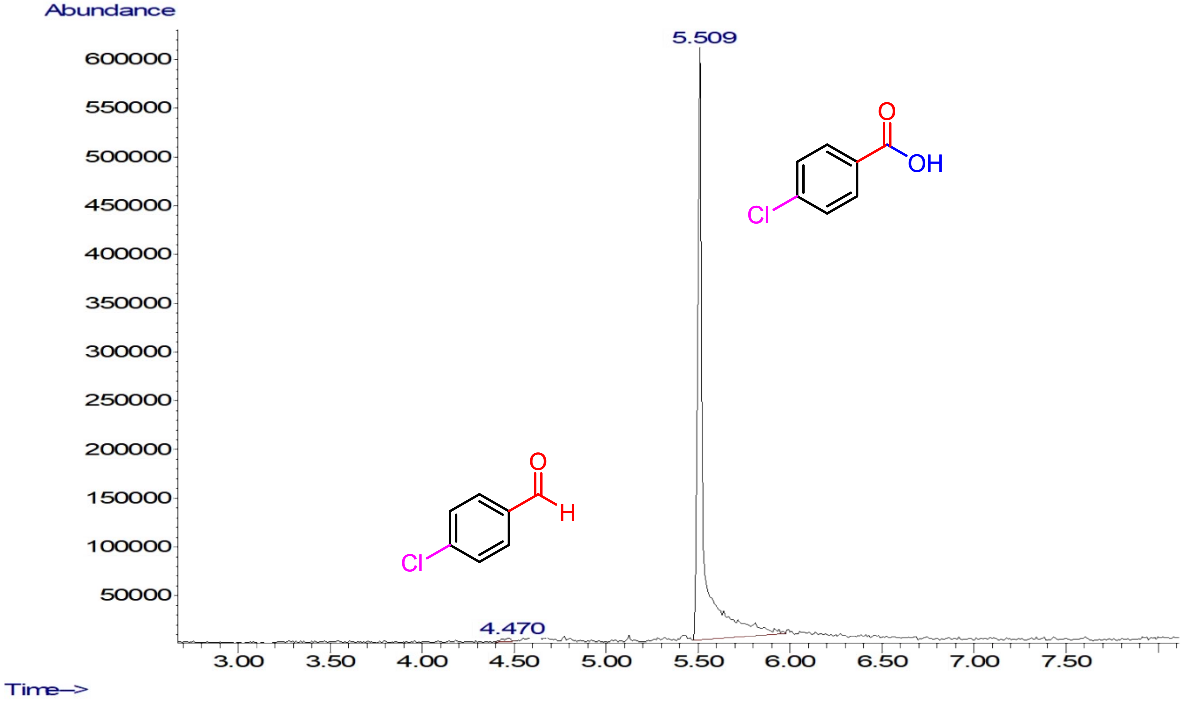


**Fig. S27a.** GC-MS trace of the reaction mixture after the oxidation of 4-chlorobenzyl alcohol.

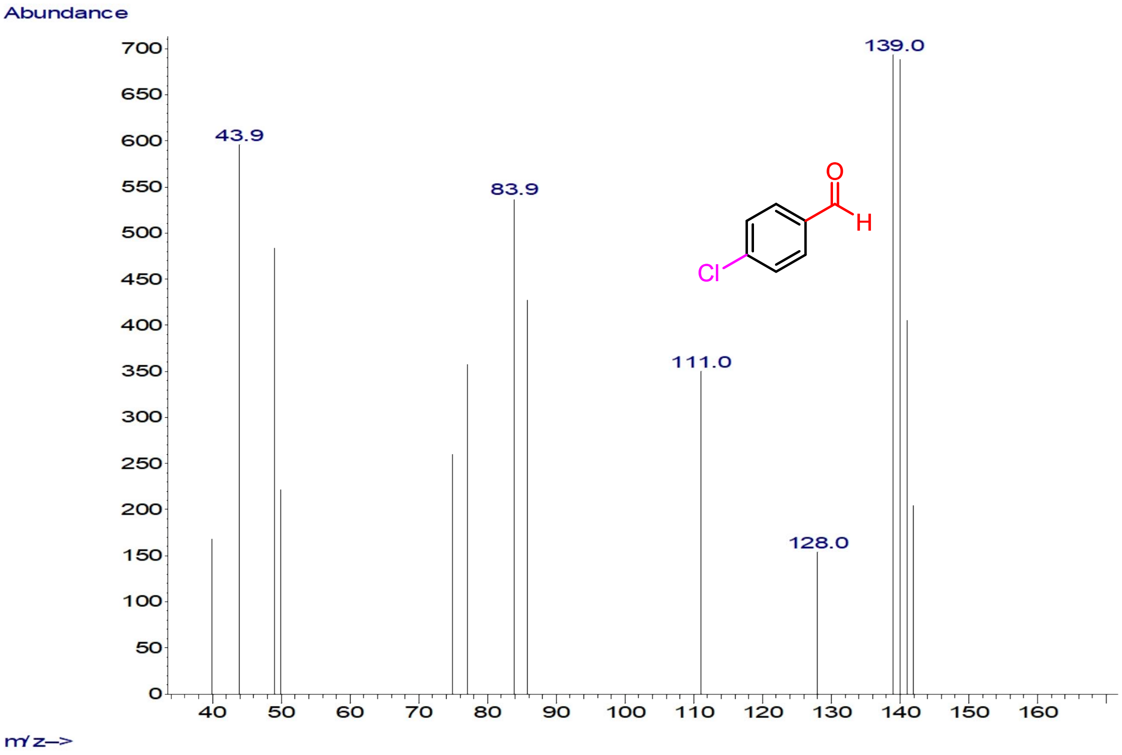


**Fig. S27b.** Mass spectra corresponding to the GC trace at 4.470 min.

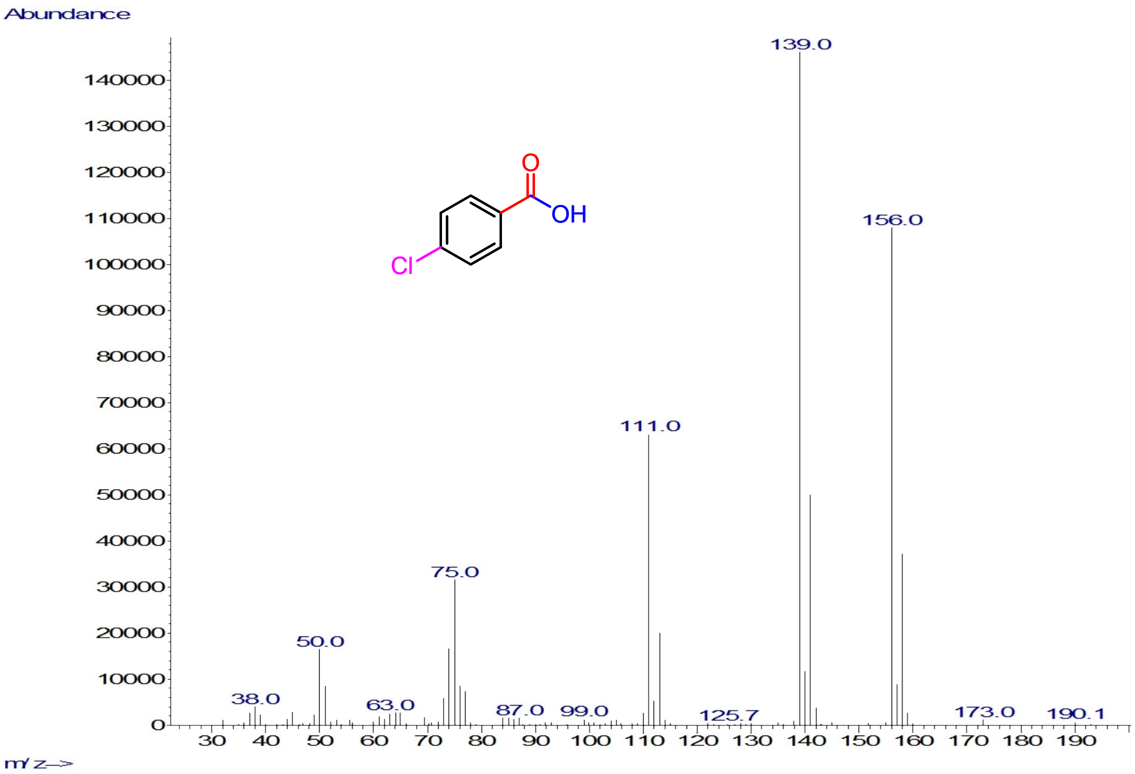


**Fig. S27c.** Mass spectra corresponding to the GC trace at 5.509 min.

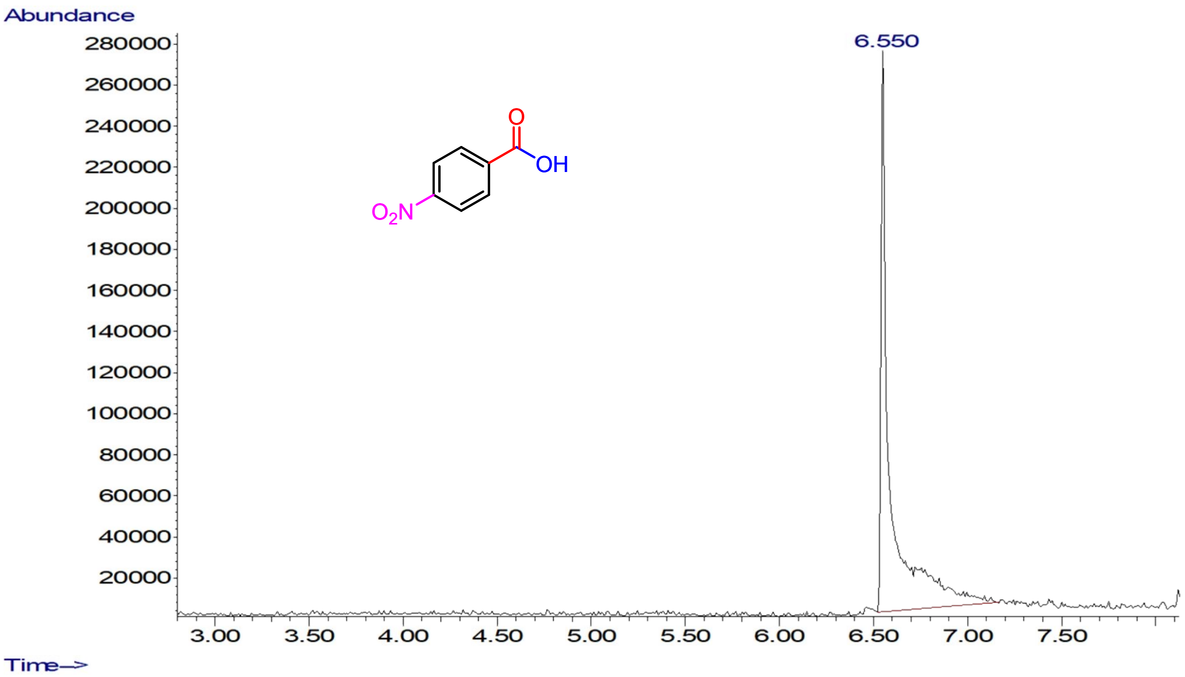


**Fig. S28a.** GC-MS trace of the reaction mixture after the oxidation of 4-nitrobenzyl alcohol.

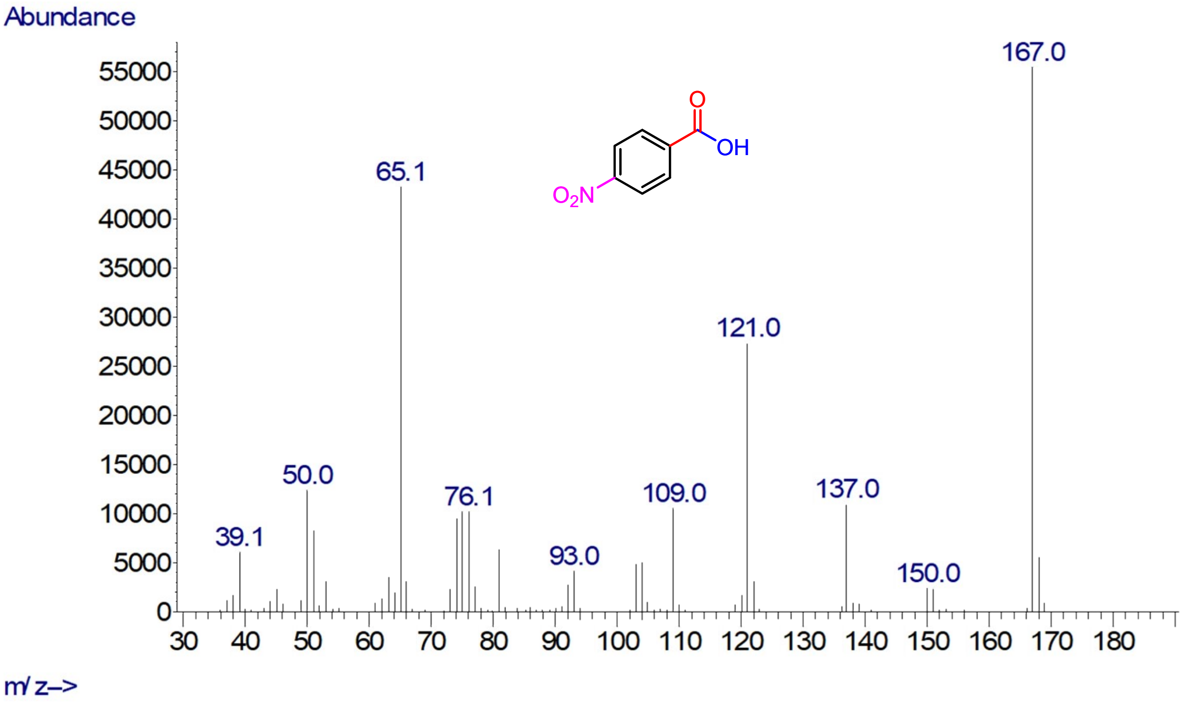


**Fig. S28b.** Mass spectra corresponding to the GC trace at 6.550 min.

**NMR spectra of the products**

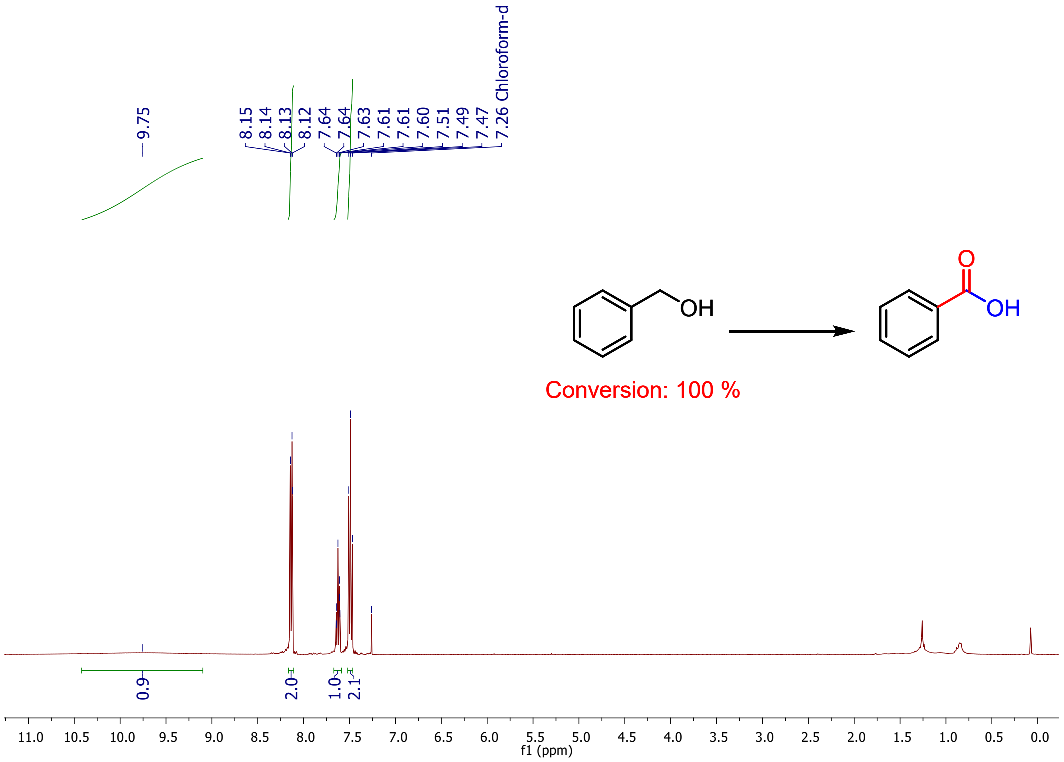


**Fig. S29.** $^{1}$H NMR spectrum of **3a** reaction mixture.

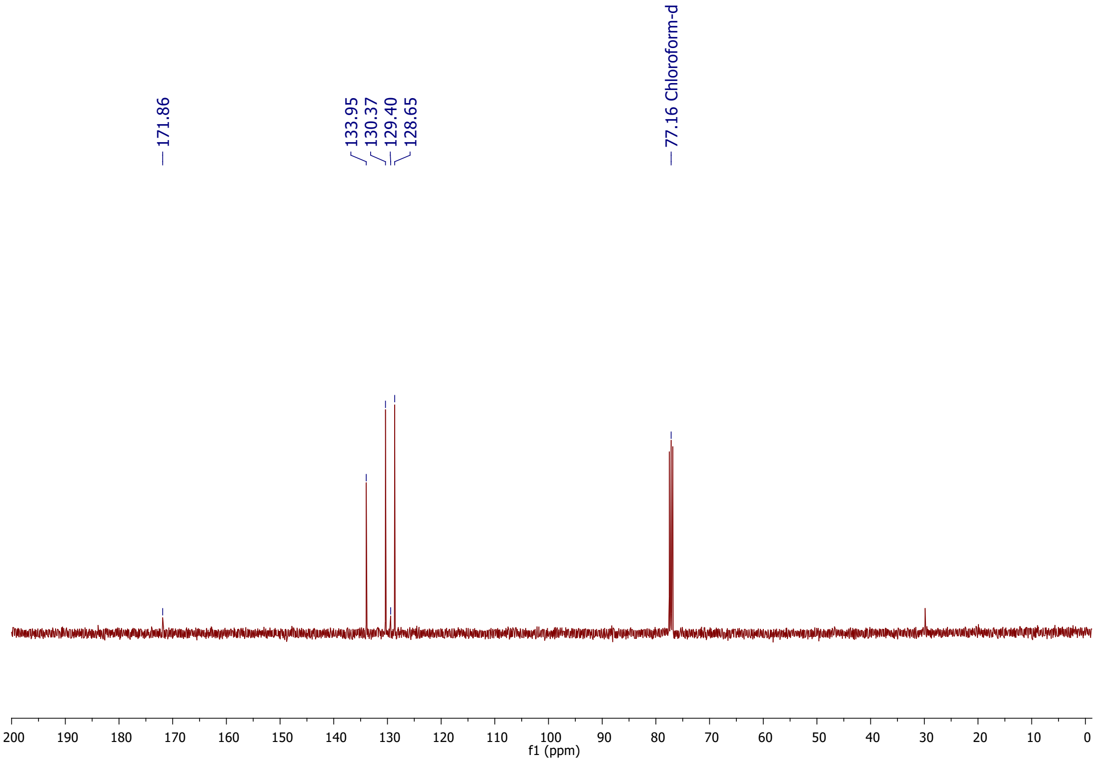


**Fig. S30.** $^{13}$C NMR spectrum of **3a** reaction mixture.

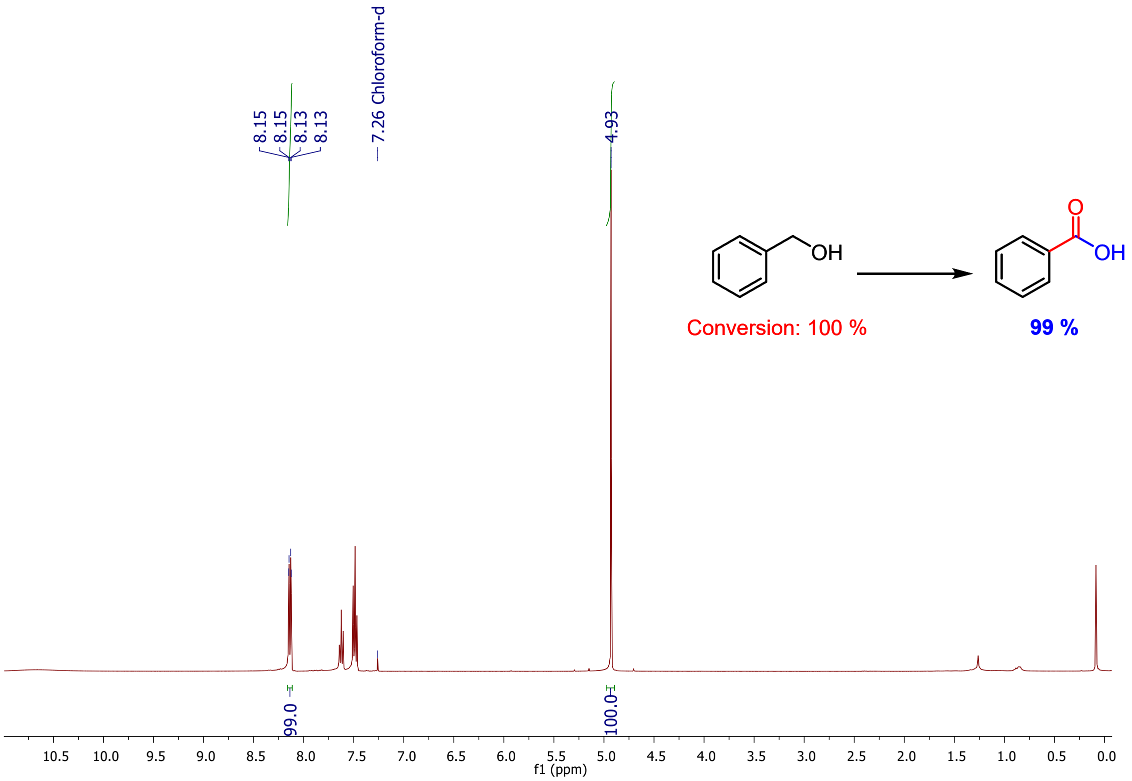


**Fig. S31.** $^{1}$H NMR spectrum of **3a** reaction mixture with an internal standard: $CH_2Br_2$.

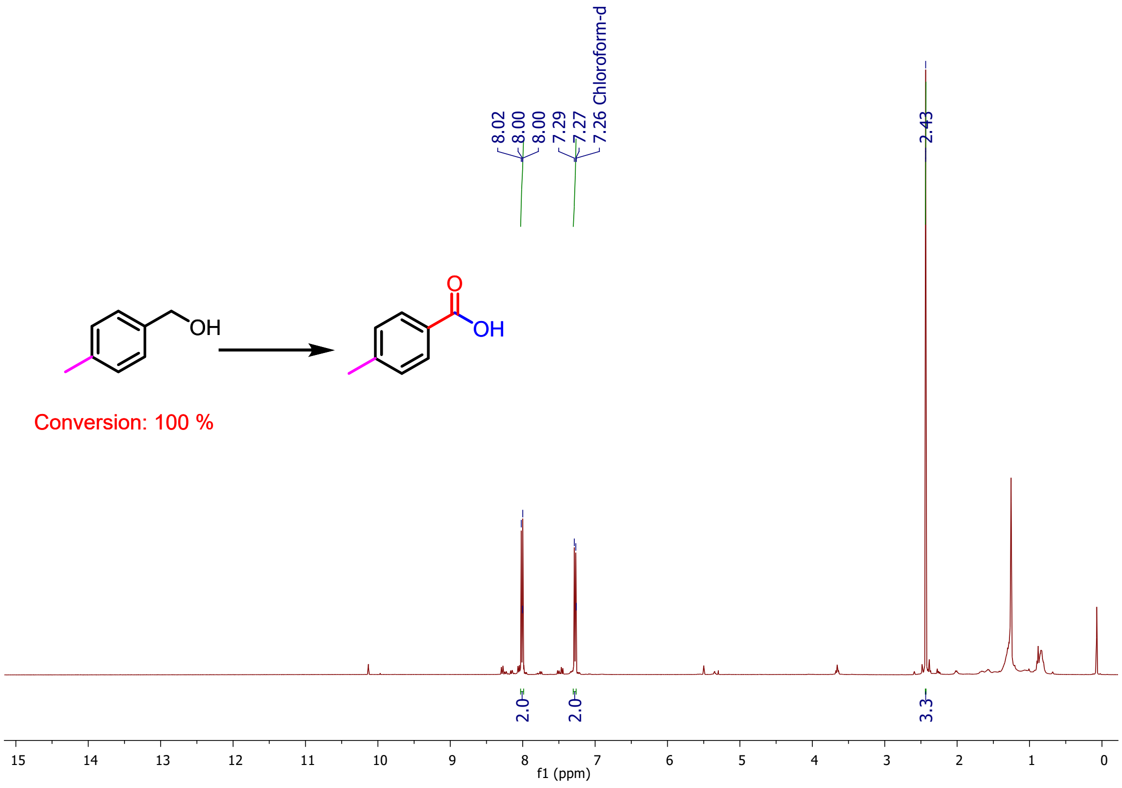


**Fig. S32.** $^{1}$H NMR spectrum of **3b** reaction mixture.

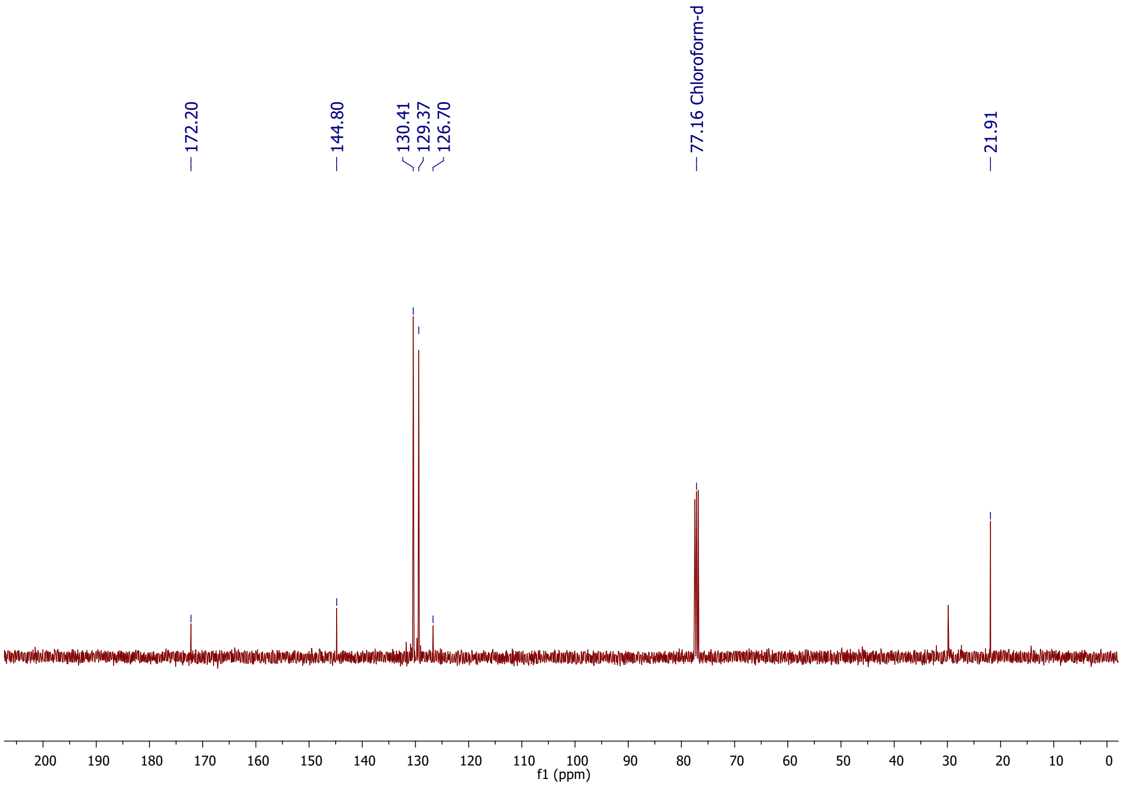


**Fig. S33.** $^{13}C$ NMR spectrum of **3b** reaction mixture.

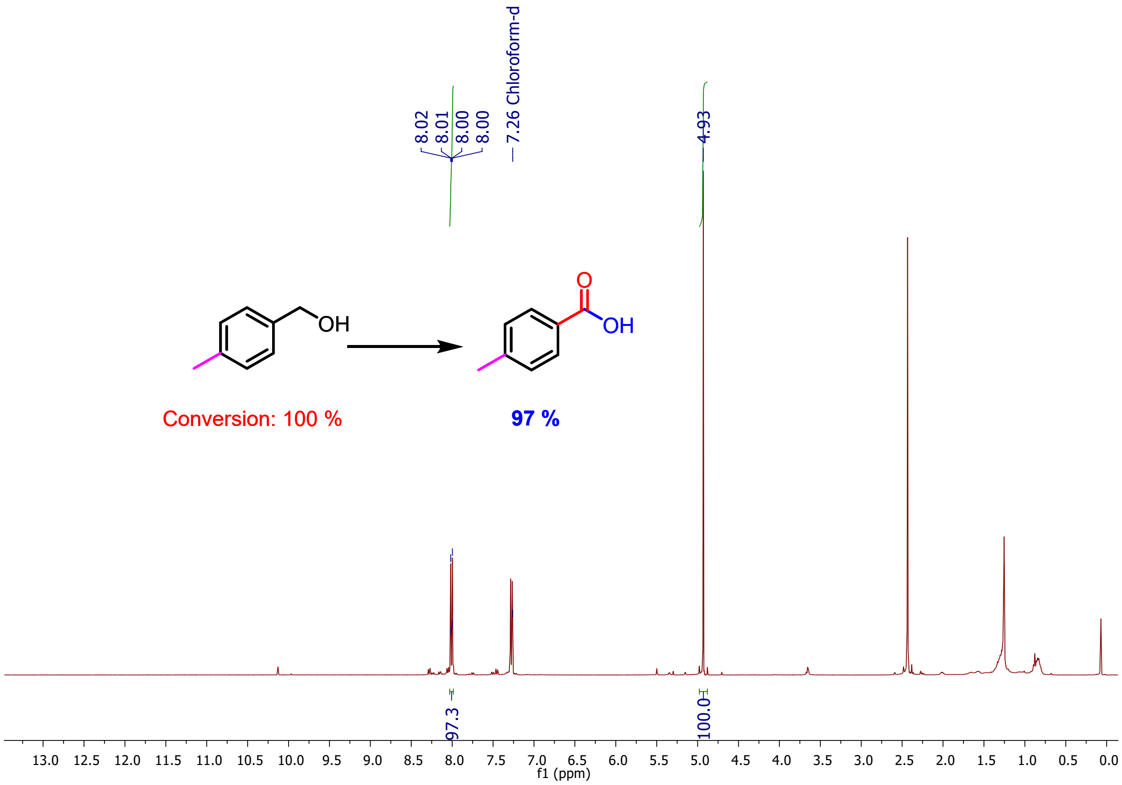


**Fig. S34.** $^{1}H$ NMR spectrum of **3b** reaction mixture with an internal standard: $CH_2Br_2$.

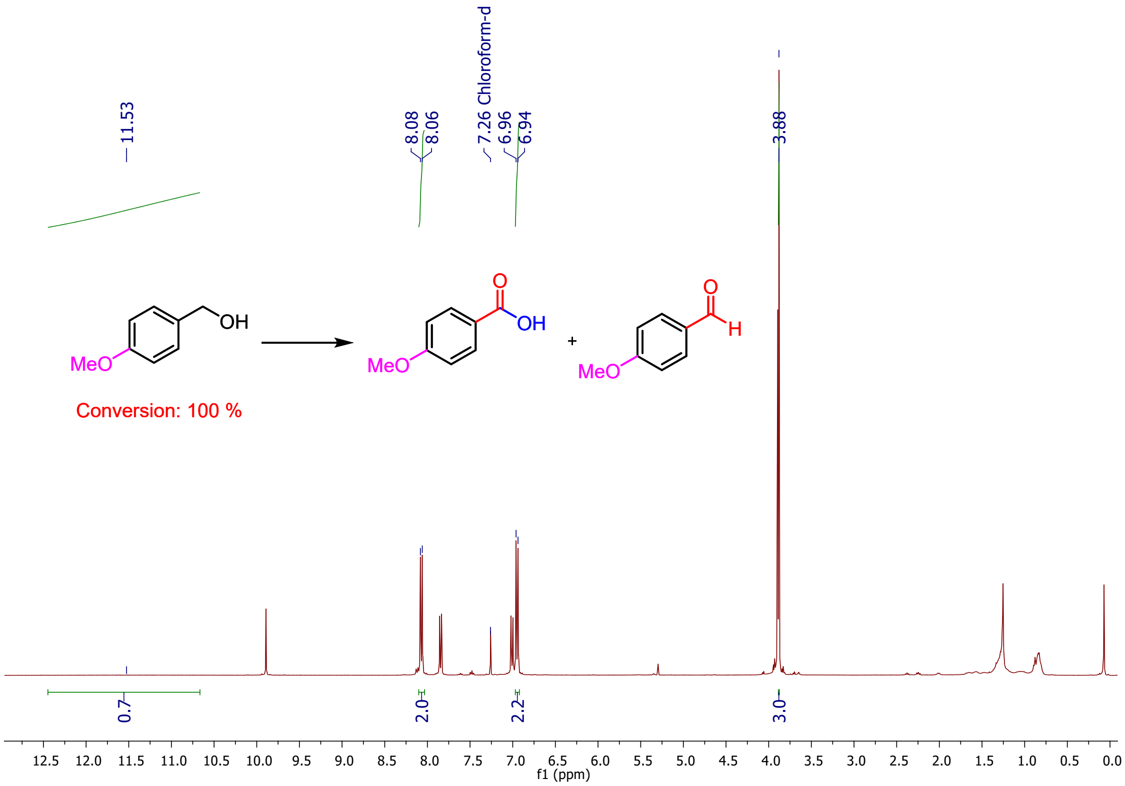


**Fig. S35.** $^{1}H$ NMR spectrum of **3c** reaction mixture.

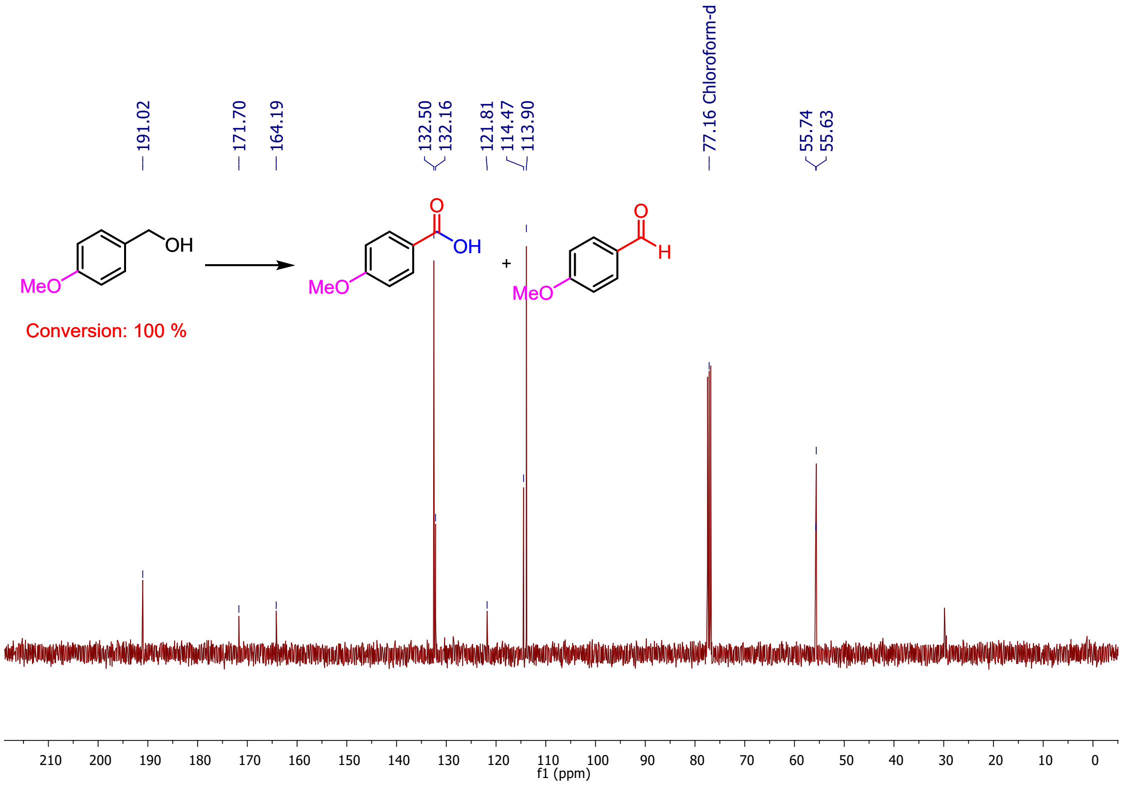


**Fig. S36.** $^{13}C$ NMR spectrum of **3c** reaction mixture.

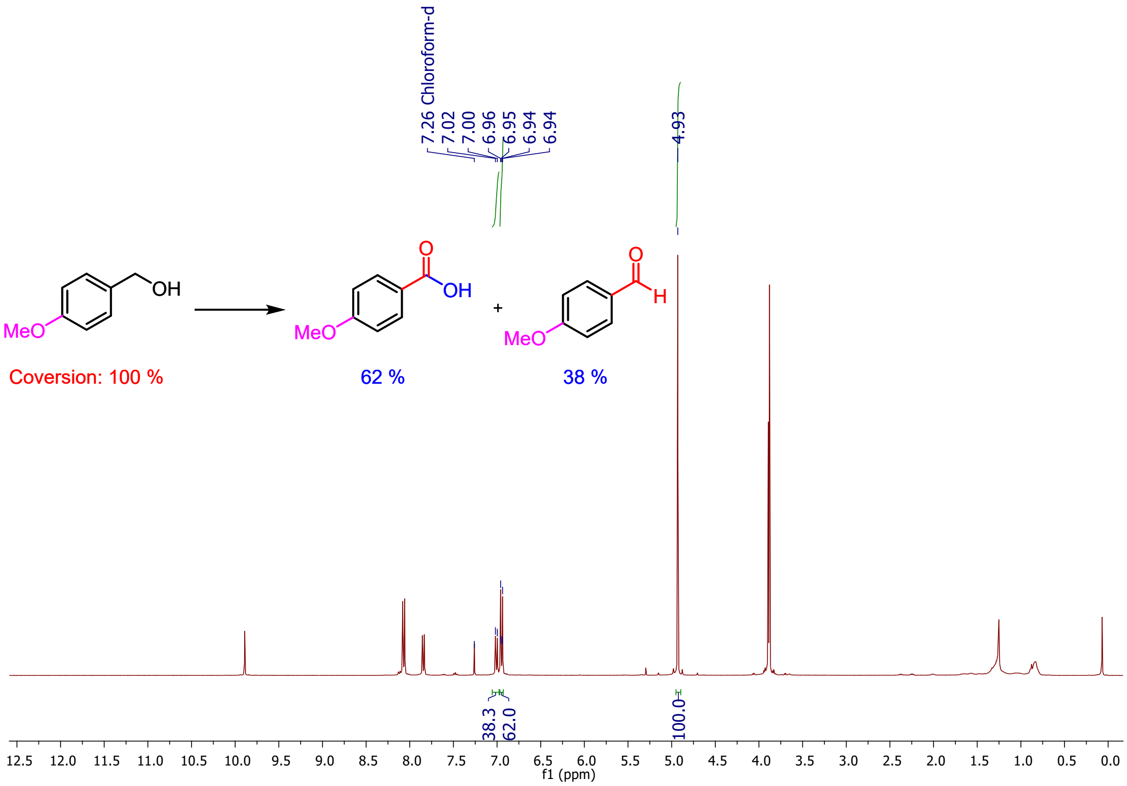


**Fig. S37.** $^{1}H$ NMR spectrum of **3c** reaction mixture with an internal standard: $CH_2Br_2$.

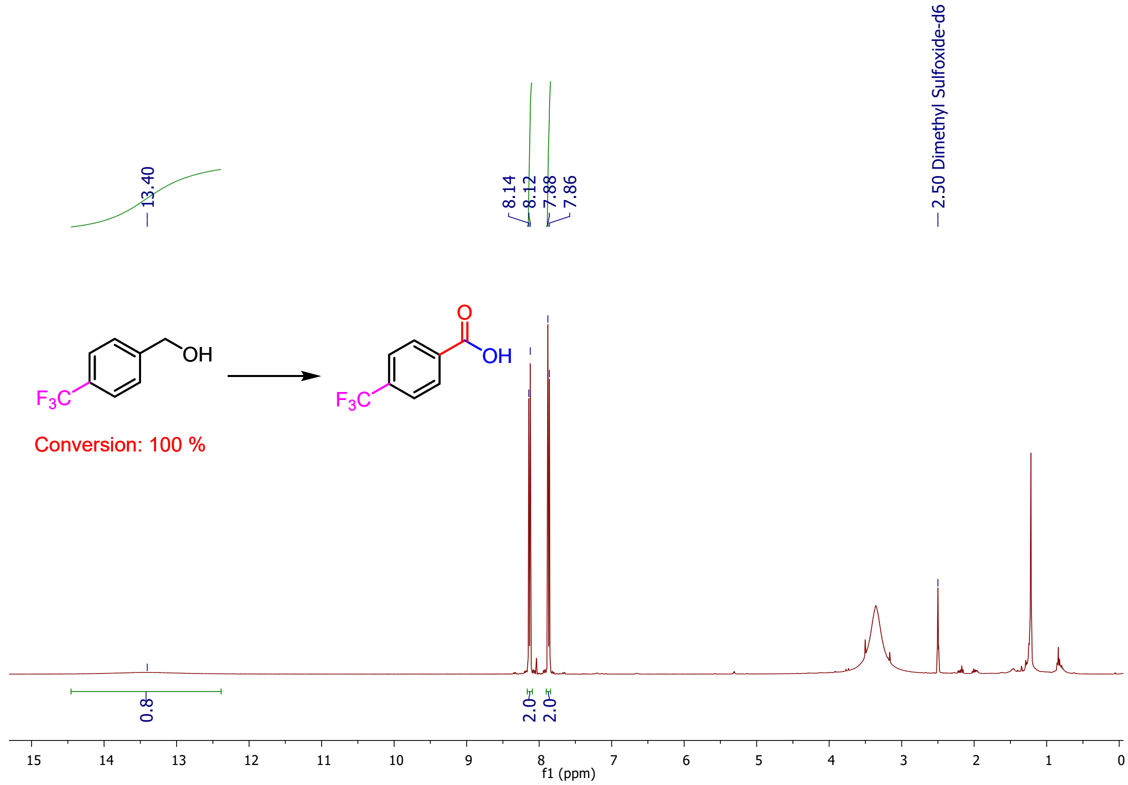


**Fig. S38.** $^{1}H$ NMR spectrum of **3d** reaction mixture.

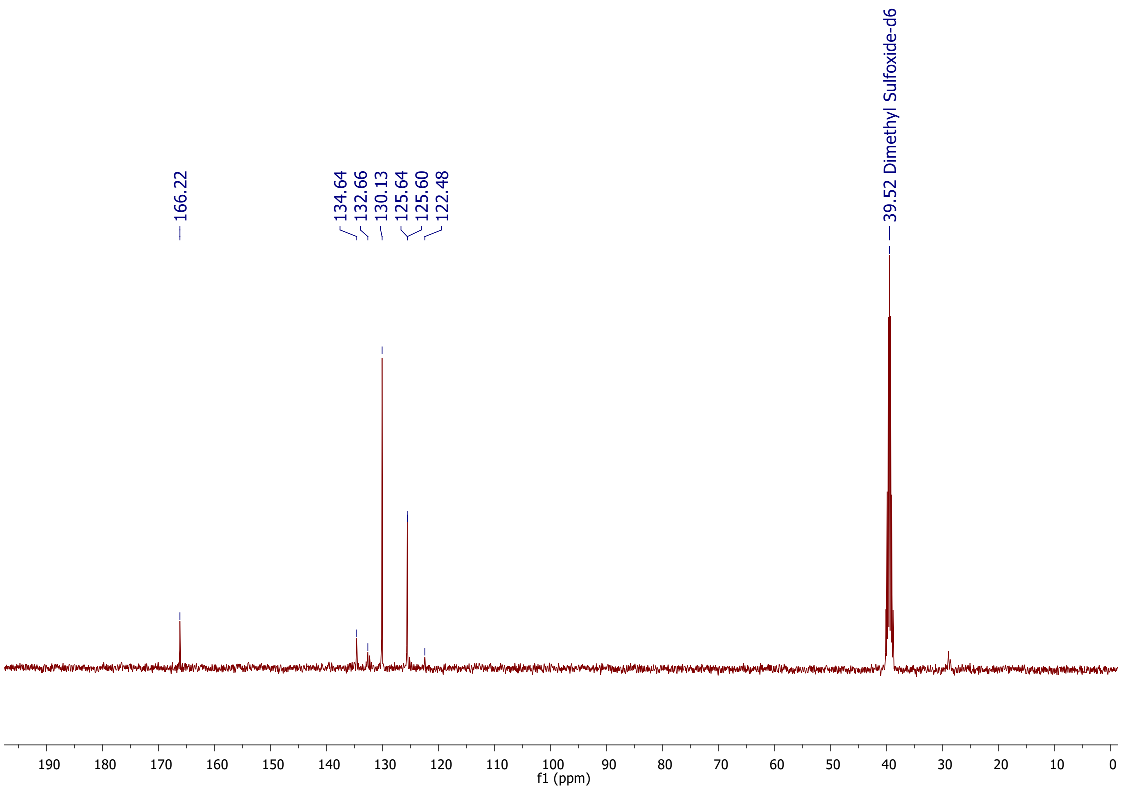


**Fig. S39.** $^{13}$C NMR spectrum of **3d** reaction mixture.

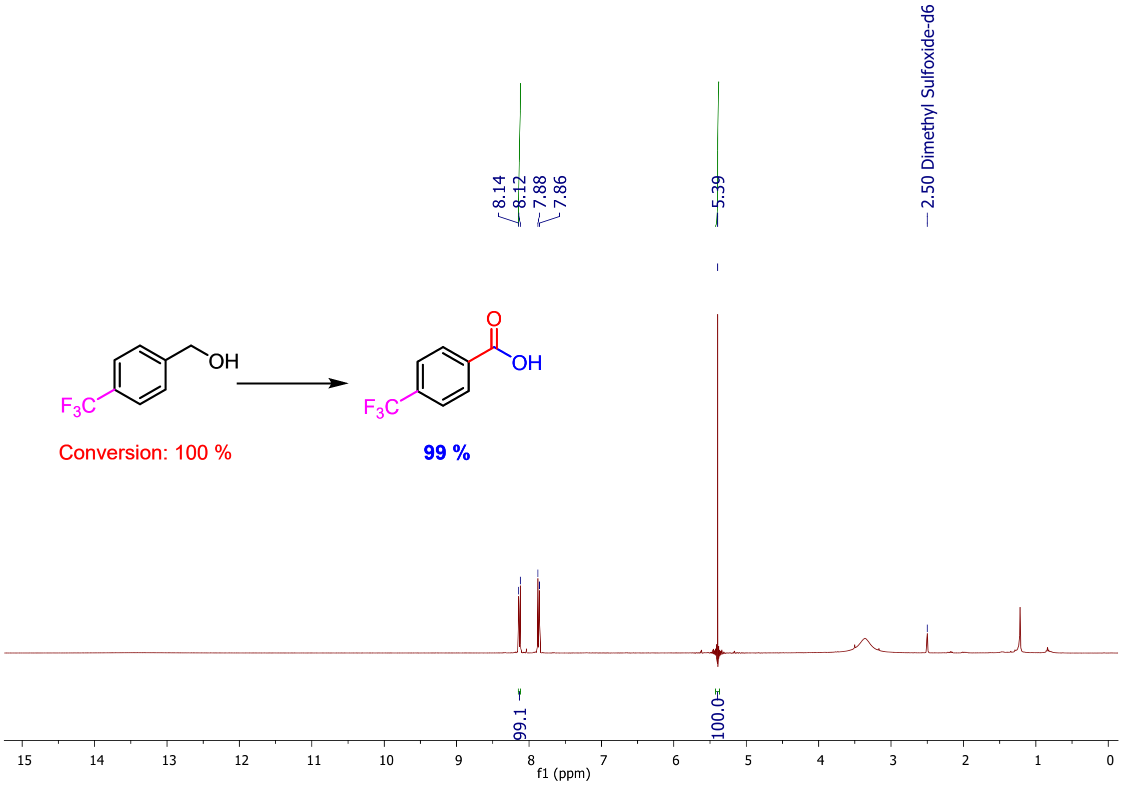


**Fig. S40.** $^{1}$H NMR spectrum of **3d** reaction mixture with an internal standard: $CH_2Br_2$.

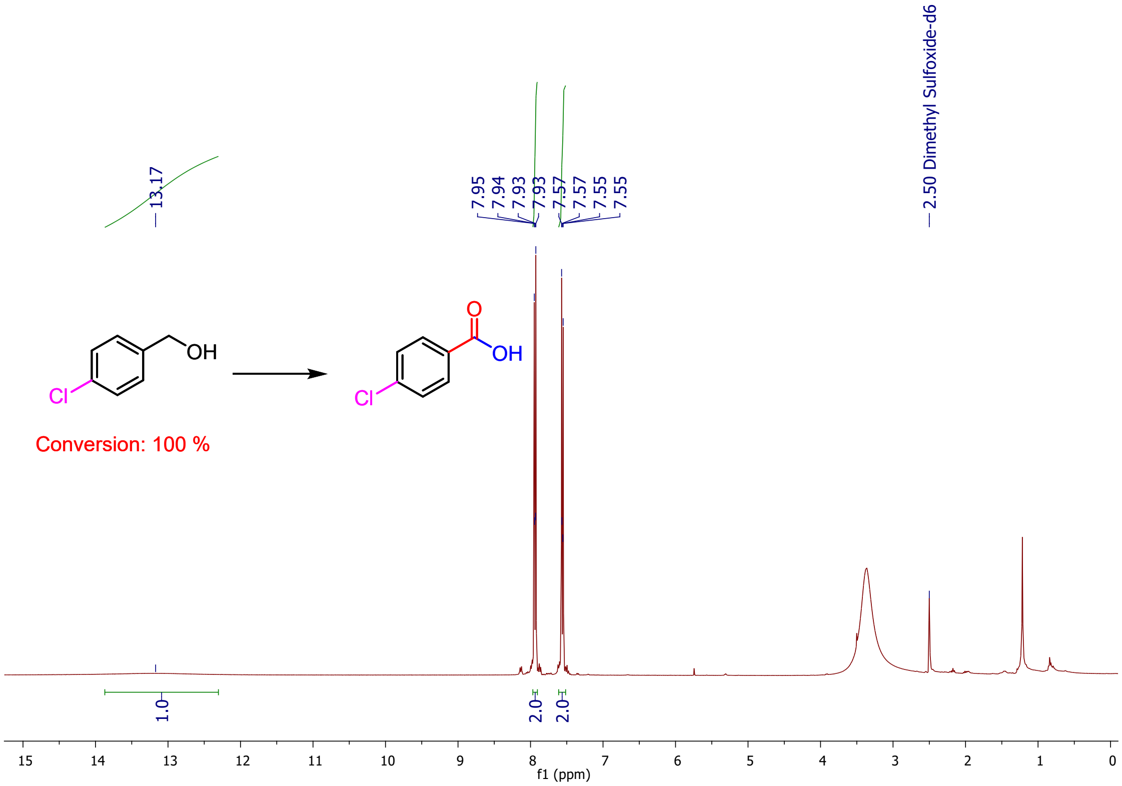


**Fig. S41.** $^{1}$H NMR spectrum of **3e** reaction mixture.

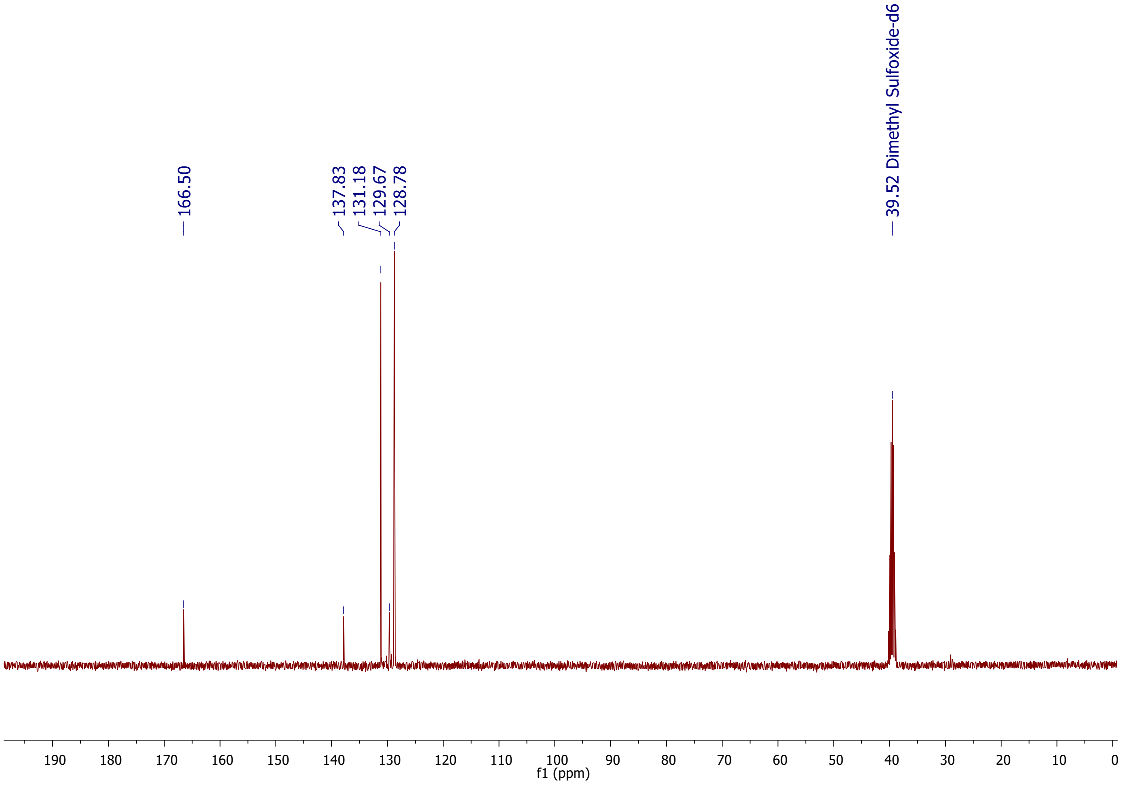


**Fig. S42.** $^{13}$C NMR spectrum of **3e** reaction mixture.

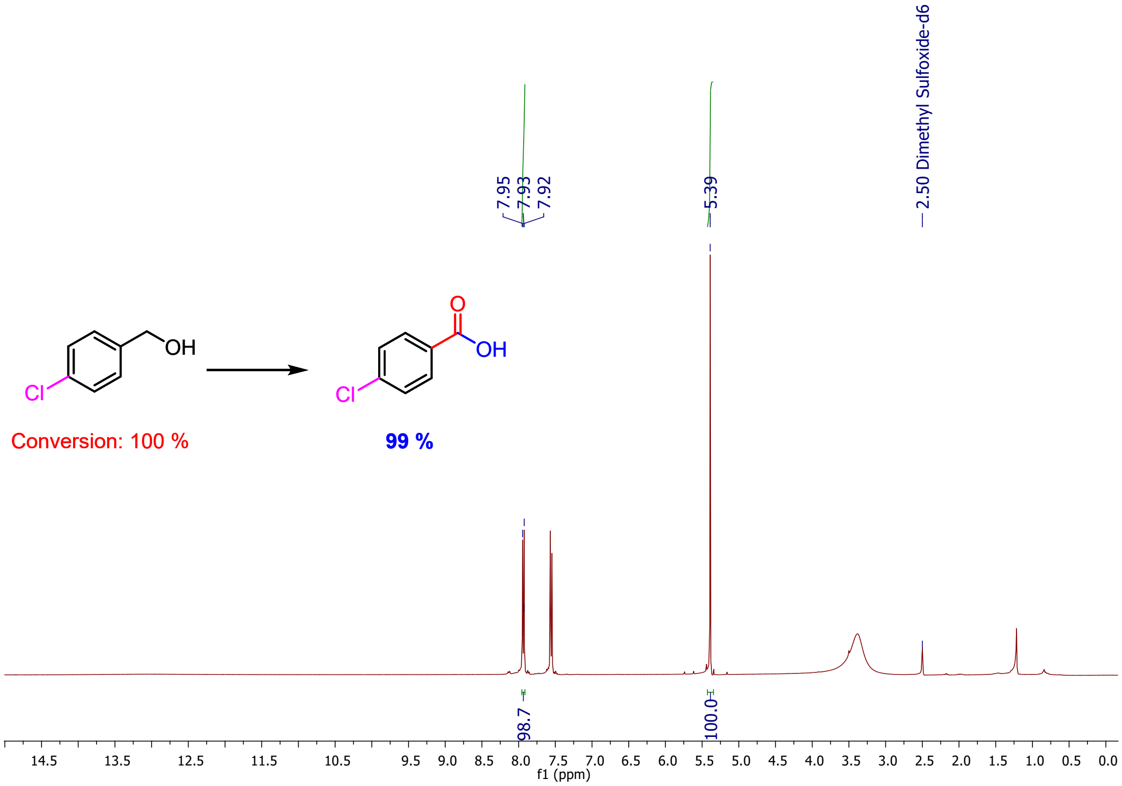


**Fig. S43.** $^{1}$H NMR spectrum of **3e** reaction mixture with an internal standard: $CH_2Br_2$.

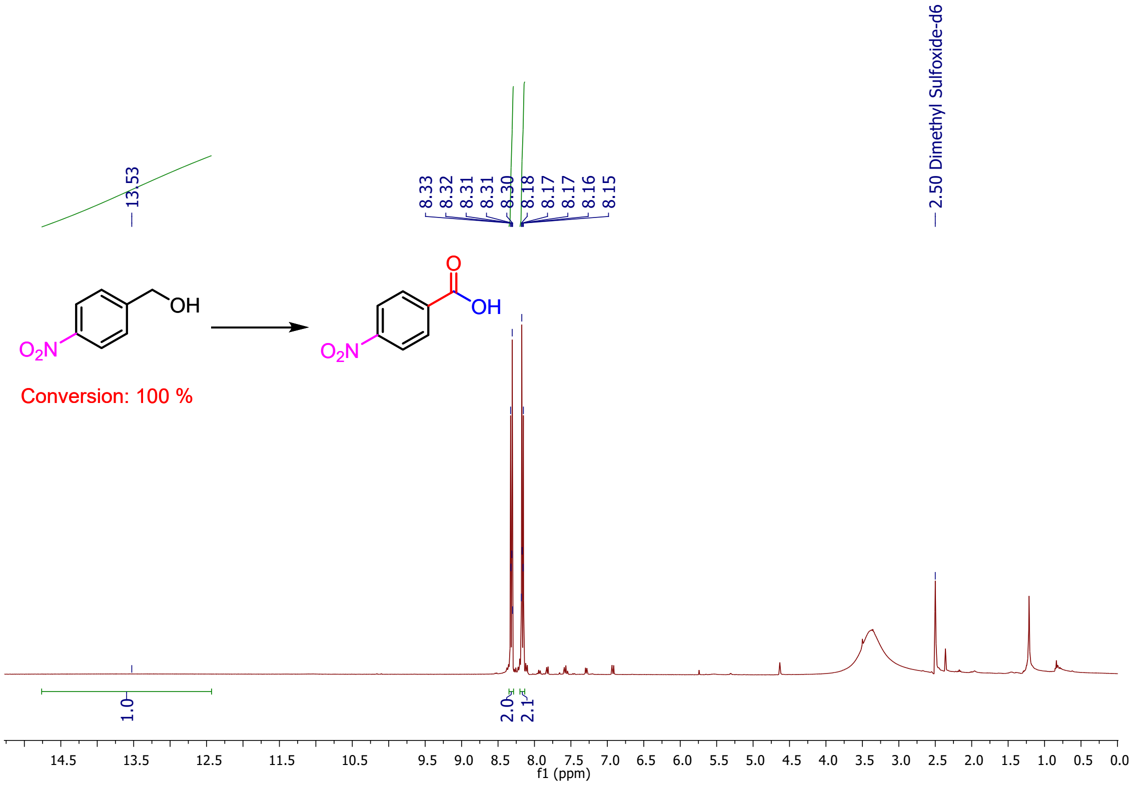


**Fig. S44.** $^{1}$H NMR spectrum of **3f** reaction mixture.

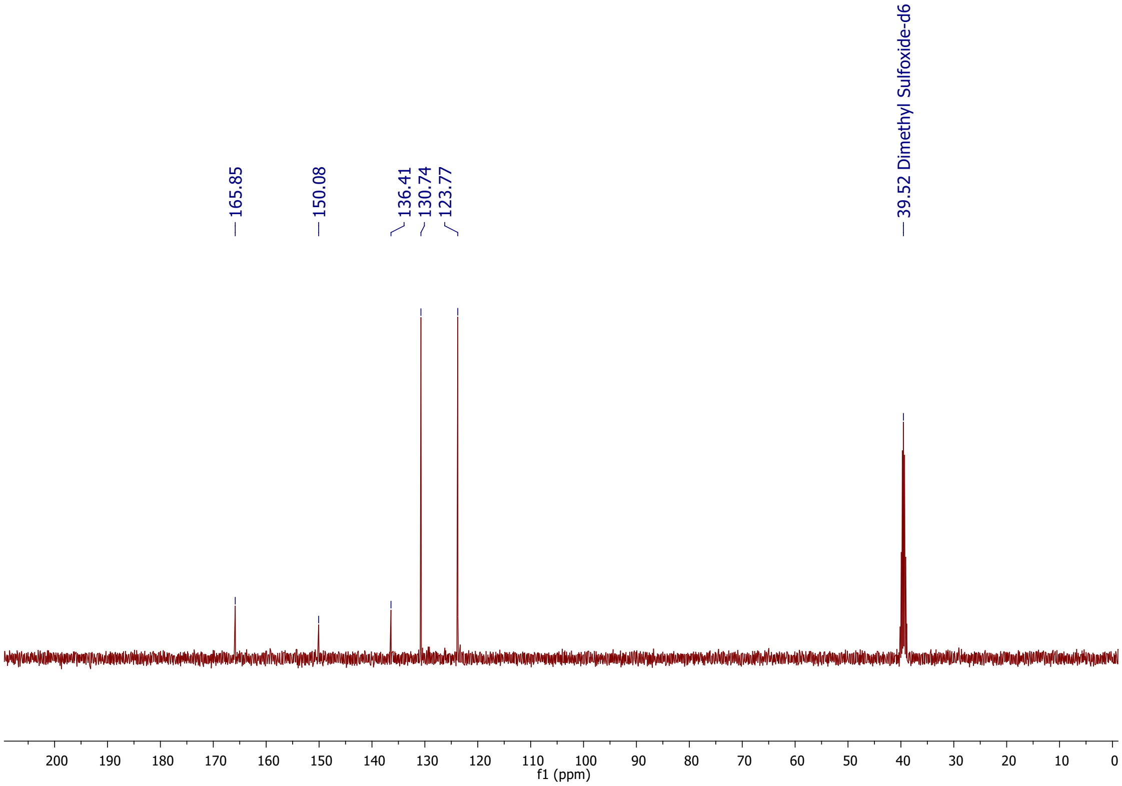


**Fig. S45.** $^{13}C$ NMR spectrum of **3f** reaction mixture.

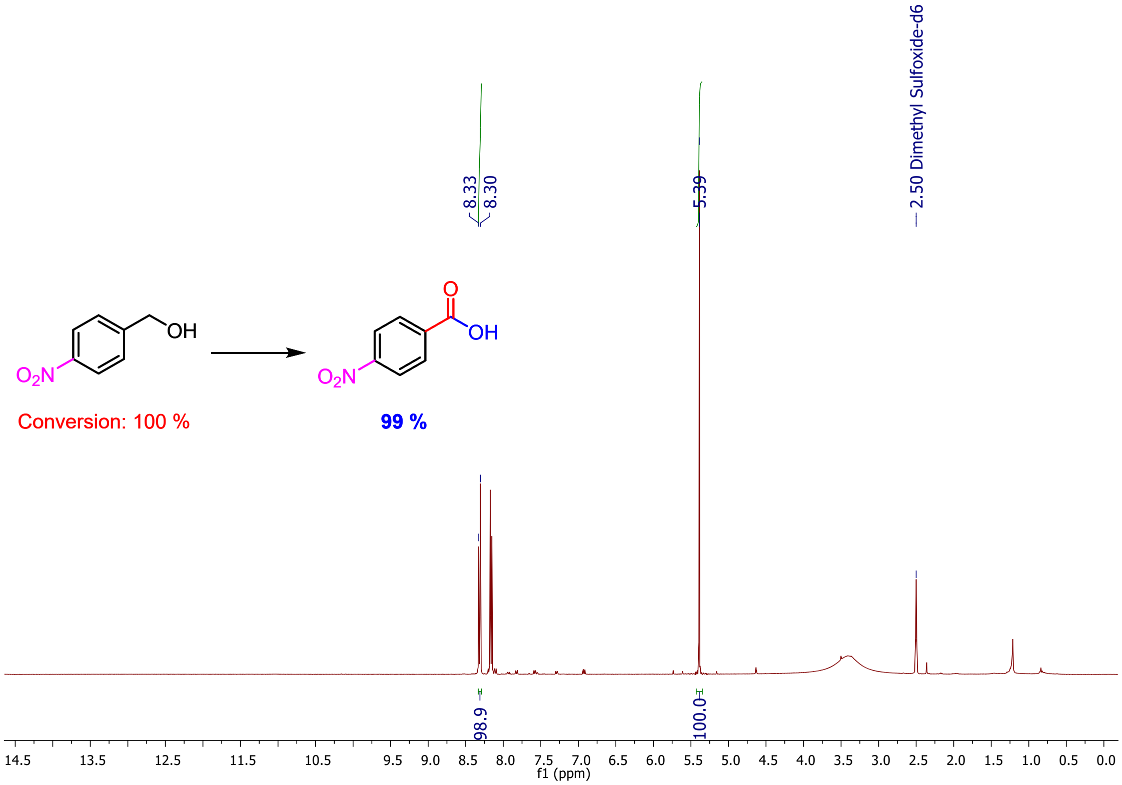


**Fig. S46.** $^{1}H$ NMR spectrum of **3f** reaction mixture with an internal standard: $CH_2Br_2$.

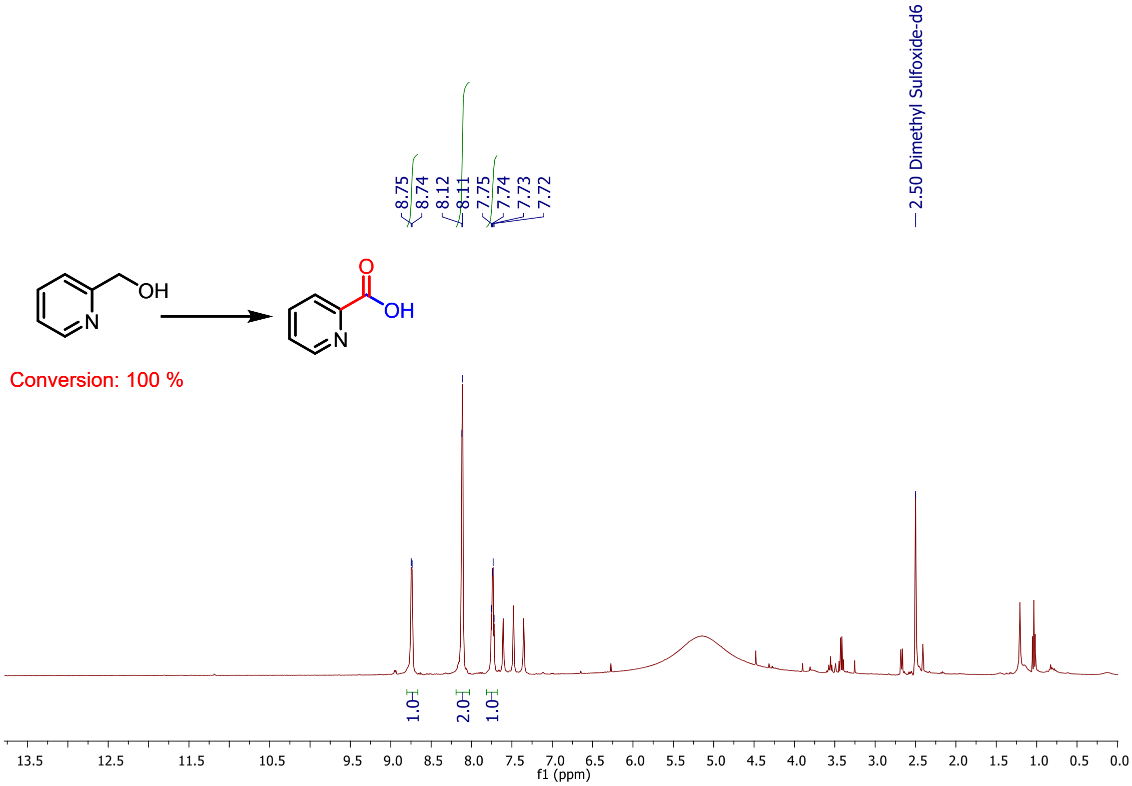


**Fig. S47.** $^{1}H$ NMR spectrum of **3g** reaction mixture.

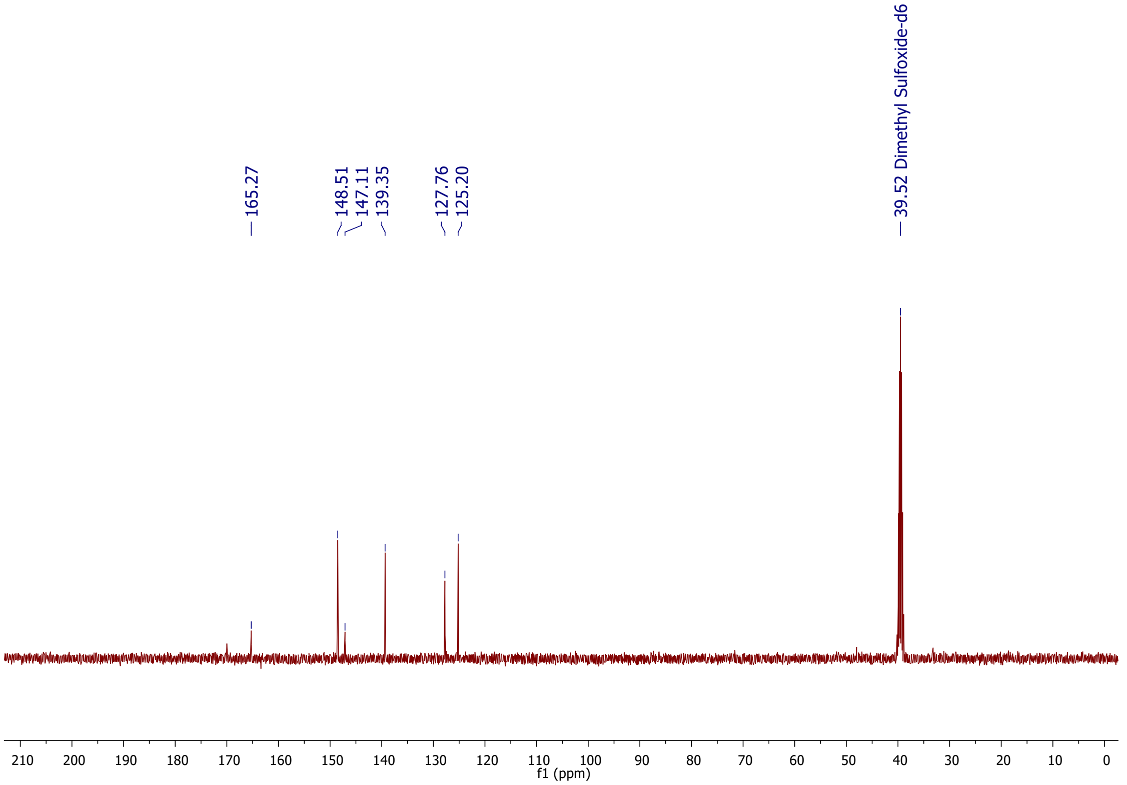


**Fig. S48.** $^{13}C$ NMR spectrum of **3g** reaction mixture.

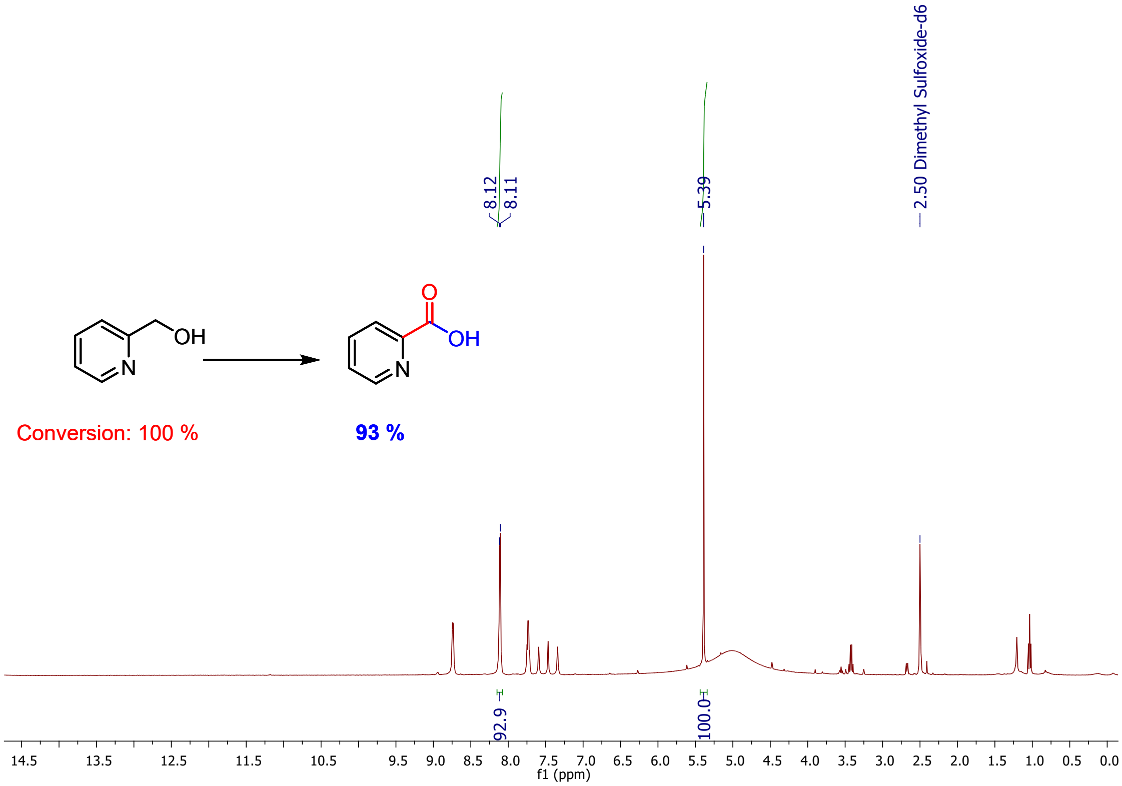


**Fig. S49.** ¹H NMR spectrum of **3g** reaction mixture with an internal standard: $CH_2Br_2$.

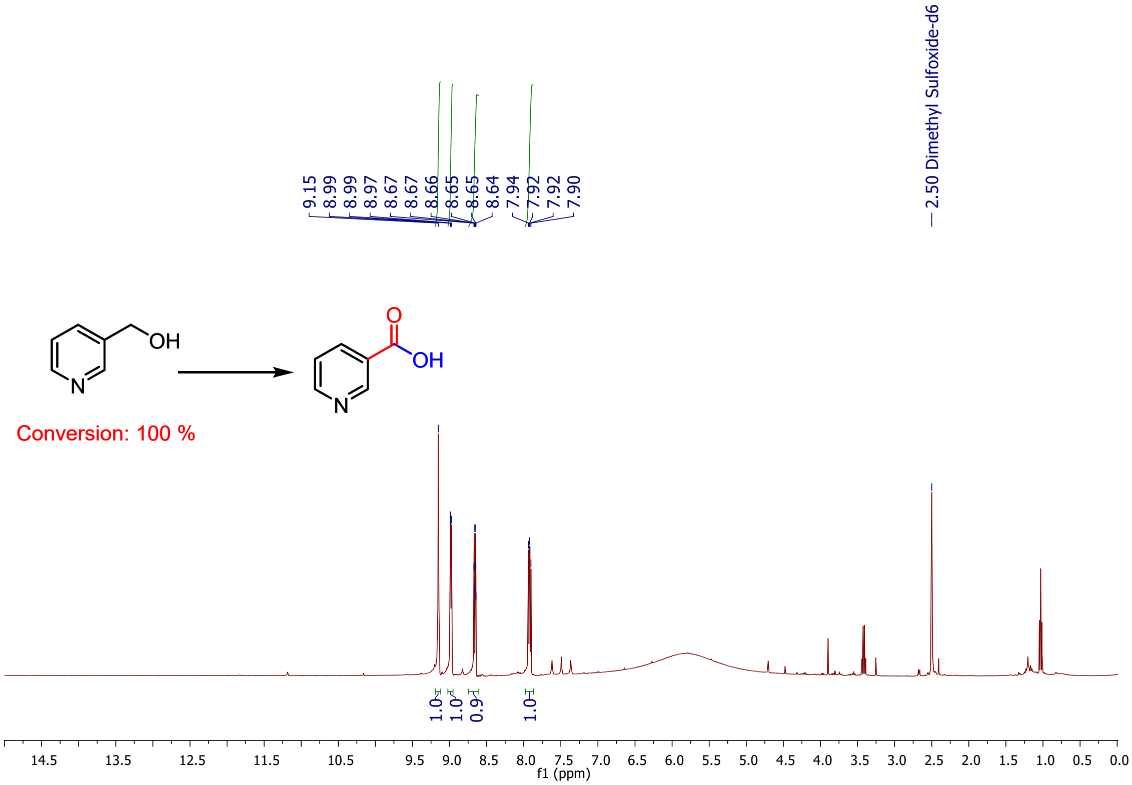


**Fig. S50.** ¹H NMR spectrum of **3h** reaction mixture.

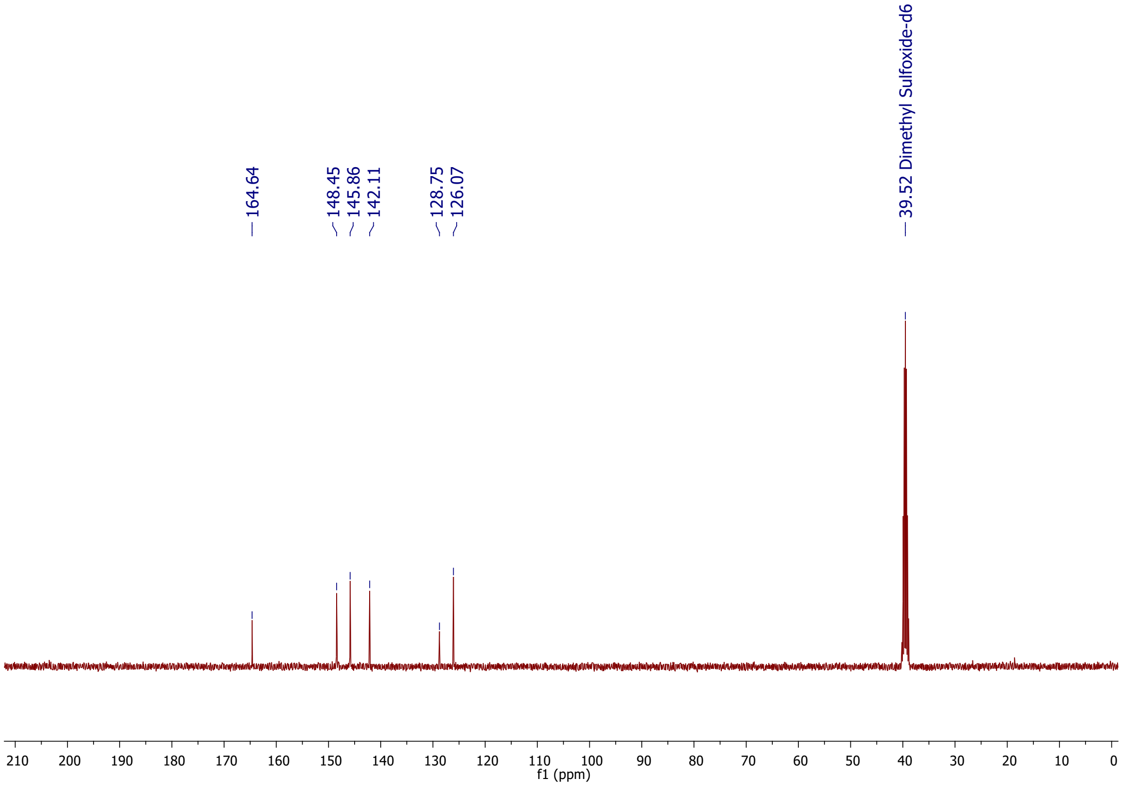


**Fig. S51.** $^{13}C$ NMR spectrum of **3h** reaction mixture.

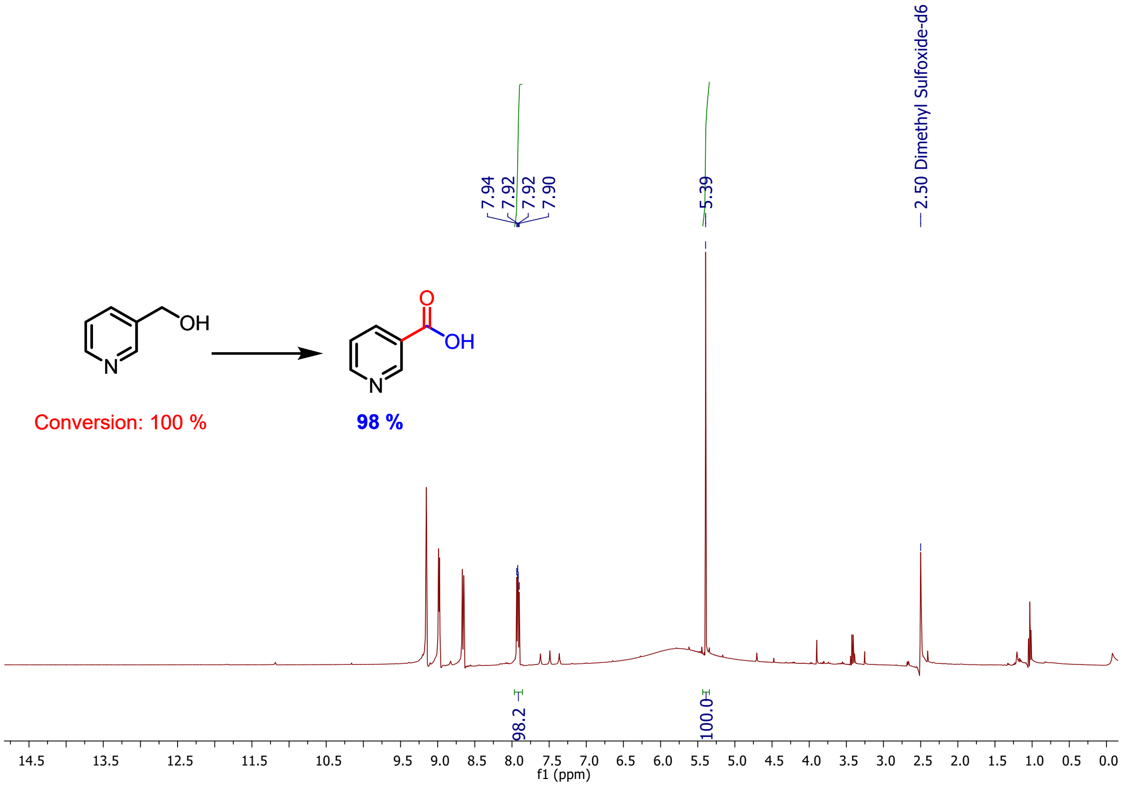


**Fig. S52.** $^{1}H$ NMR spectrum of **3h** reaction mixture with an internal standard: $CH_2Br_2$.

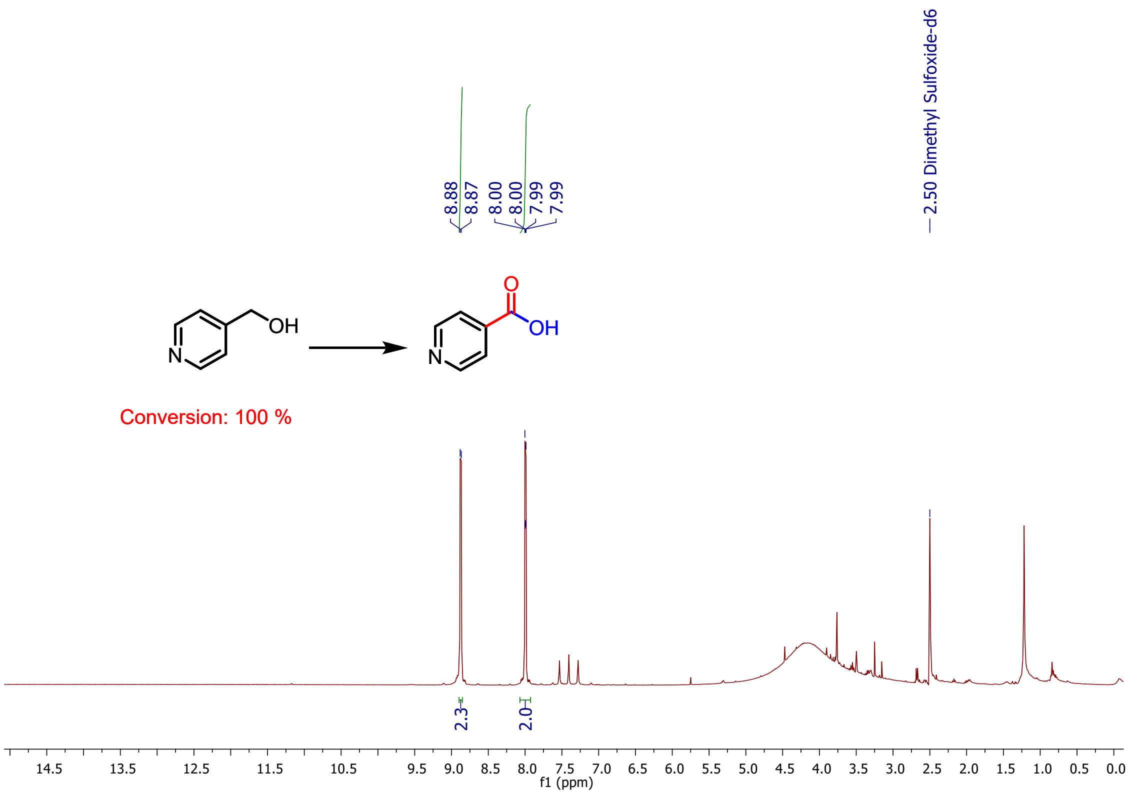


**Fig. S53.** $^{1}$H NMR spectrum of **3i** reaction mixture.

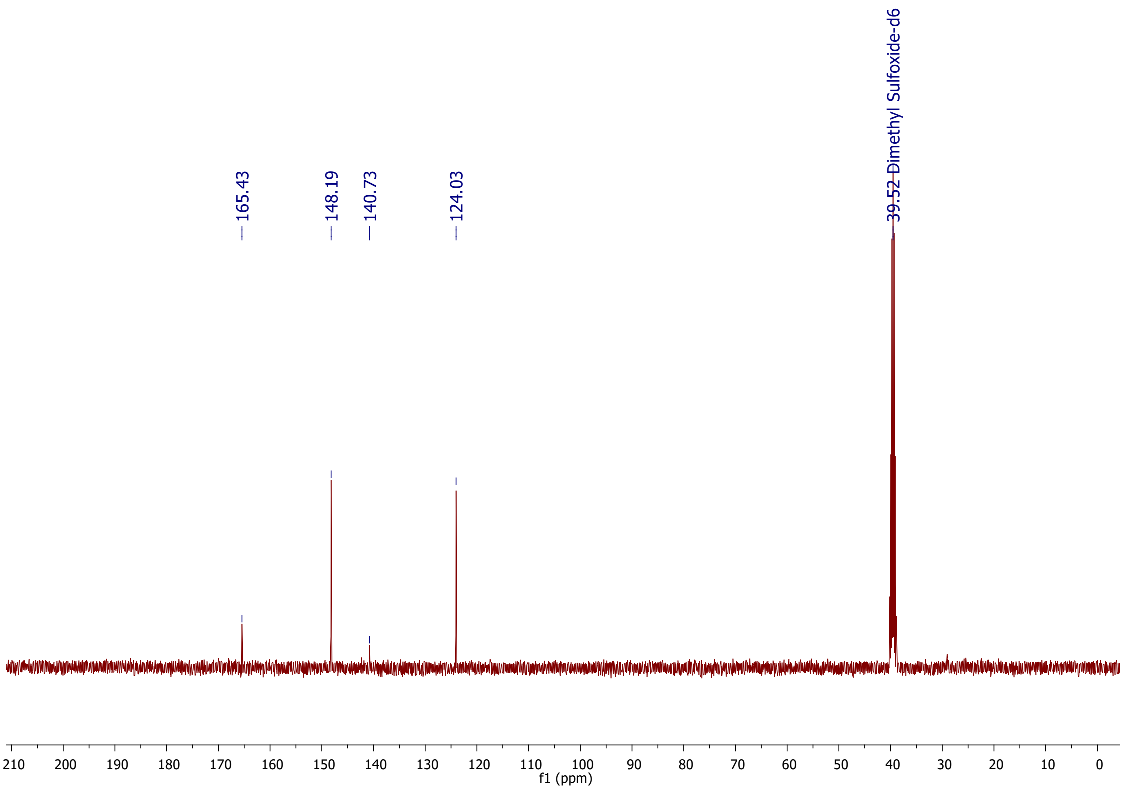


**Fig. S54.** $^{13}$C NMR spectrum of **3i** reaction mixture.

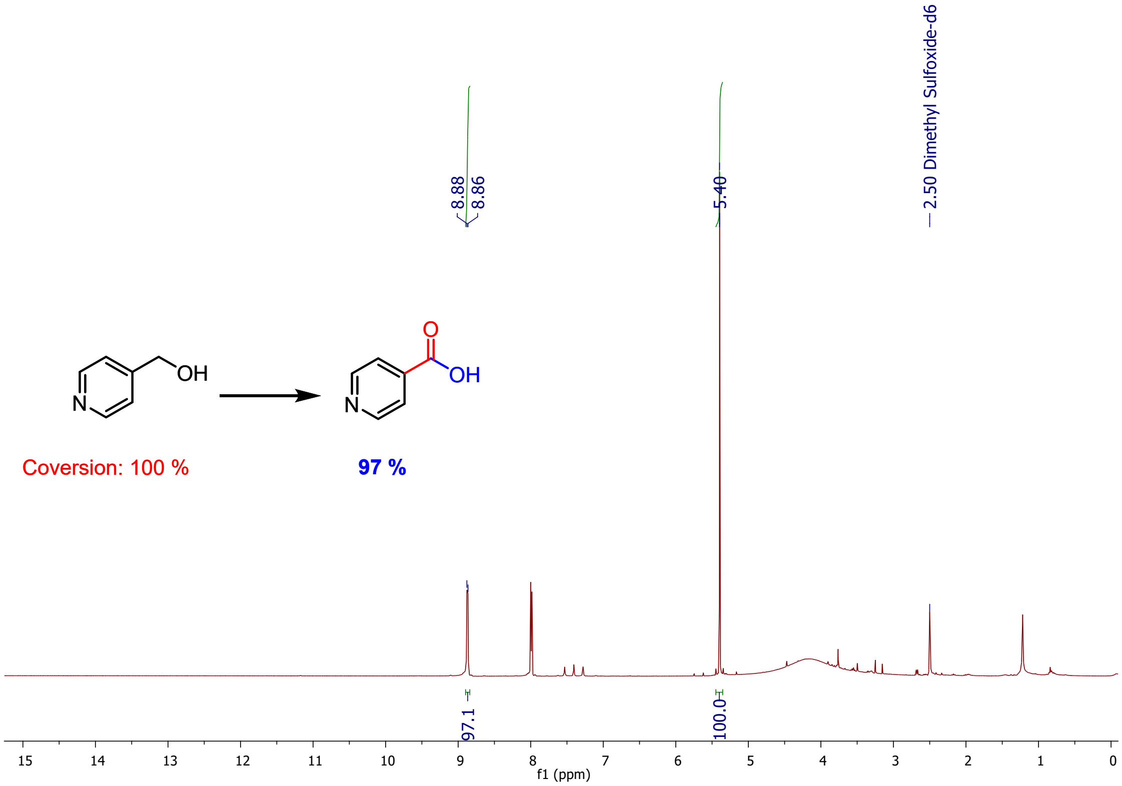


**Fig. S55.** $^1$H NMR spectrum of **3i** reaction mixture with an internal standard: $CH_2Br_2$.

## Electronic Supplementary Information References


(1) V. W. h. Lau and B. V. Lotsch, *Adv. Energy Mater.*, 2022, **12**, 2101078.

(2) F. Ichihara, F. Sieland, H. Pang, D. Philo, A. T. Duong, K. Chang, T. Kako, D. W. Bahnemann and J. Ye, *J. Phys. Chem. C*, 2020, **124**, 1292-1302.

(3) T. Yoshihara, R. Katoh, A. Furube, Y. Tamaki, M. Murai, K. Hara, S. Murata, H. Arakawa and M. Tachiya, *J. Phys. Chem. B*, 2004, **108**, 3817-3823.
